# Tuning the redox non-innocence of a phenalenyl ligand toward efficient nickel-assisted catalytic hydrosilylation†
†Electronic supplementary information (ESI) available: Detailed experimental procedures, spectra (NMR and HRMS), CV, crystallographic details, and coordinates of the computed structures. CCDC 1518117. For ESI and crystallographic data in CIF or other electronic format see DOI: 10.1039/c7sc04687a


**DOI:** 10.1039/c7sc04687a

**Published:** 2018-01-31

**Authors:** Gonela Vijaykumar, Anand Pariyar, Jasimuddin Ahmed, Bikash Kumar Shaw, Debashis Adhikari, Swadhin K. Mandal

**Affiliations:** a Department of Chemical Sciences , Indian Institute of Science Education and Research-Kolkata , Mohanpur-741246 , India . Email: swadhin.mandal@iiserkol.ac.in; b Department of Chemical Sciences , Indian Institute of Science Education and Research Mohali , SAS Nagar 140306 , India . Email: adhikari@iisermohali.ac.in

## Abstract

In this report, a ligand-redox assisted catalytic hydrosilylation has been investigated. A phenalenyl ligand coordinated nickel complex has been utilized as an electron reservoir to develop a base metal-assisted catalyst, which very efficiently hydrosilylates a wide variety of olefin substrates under ambient conditions. A mechanistic investigation revealed that a two-electron reduced phenalenyl based biradical nickel complex plays the key role in such catalysis. The electronic structure of the catalytically active biradical species has been interrogated using EPR spectroscopy, magnetic susceptibility measurements, and electronic structure calculations using a DFT method. Inhibition of the reaction by a radical quencher, as well as the mass spectrometric detection of two intermediates along the catalytic loop, suggest that a single electron transfer from the ligand backbone initiates the catalysis. The strategy of utilising the redox reservoir property of the ligand ensures that the nickel is not promoted to an unfavorable oxidation state, and the fine tuning between the ligand and metal redox orbitals elicits smooth catalysis.

## Abstract 2

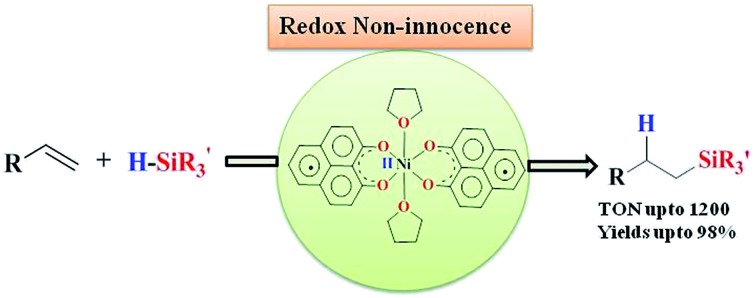
The hydrosilylation of olefins by a nickel(ii) catalyst assisted by a redox non-innocent phenalenyl (PLY) ligand is reported.

## Introduction

Recently, base metal catalysts have garnered tremendous attention since these can be ideal surrogates for scarce, expensive and often toxic 4d and 5d late transition metals.^1^ The current strong interest in developing base metal catalysts necessitates taming the metal to carry out two-electron chemistry where the metal is usually prone to perform one-electron redox reactions. The co-operative action of the ancillary ligand with the coordinated 3d metal might be an effective strategy to facilitate such a desirable multi-electron redox reaction with the metal.^2^ Achieving co-operative catalysis between the base metal and the ligand is probable if the latter behaves as a reservoir for the redox equivalent. An appropriate redox tuning between the ligands and the metal center can facilitate the redox chemistry without promoting the metal to an unusual and unfavorable oxidation state, which generally slows down the catalytic process. Seminal work from Chirik, Heyduk, Abu-Omar and Soper has showcased that metal-centric two electron chemistry is viable when redox non-innocent ligands are electronically tuned with the metals.^3^–^5^ As an alternative to a ligand assisted metal-centered two-electron redox process, it can also be conceived that the ligand’s potential to behave as an electron reservoir may trigger radical chemistry at its backbone.^6^

To design an efficient base metal-assisted catalyst, herein we have introduced an odd alternant hydrocarbon, phenalenyl (PLY) based ligand. PLY is a well-known building block for constructing organic radical based materials due to its ability to exist in three redox active states, such as cation, neutral radical and anion, by accepting the corresponding electrons into its nonbonding molecular orbital (NBMO).^7^ In this work, we take advantage of the NBMO in a metal coordinated PLY based ligand which readily stabilizes the radical state,^8^ making it a unique redox non-innocent ligand. We have recently established that metal coordination of a phenalenyl ligand is essential for the ready acceptance of electrons in the PLY ligand backbone.^9^ In this way, we have constructed a zinc-PLY based spin memory device,9*a* and designed a PLY based iron(iii) complex displaying an excellent electrocatalytic property as a cathode material for one compartment membraneless H_2_O_2_ fuel cells.9*b* Very recently, the development of metal coordinated PLY radicals and their use in diverse areas spanning from catalysis to spin-electronics have been reviewed.^10^ In this work, we hypothesize that electrons injected into the metal coordinated PLY system *via* chemical reduction can be stored and utilized during a catalytic process, which can avoid attaining an unfavorable oxidation state of the metal center. This report further discloses that the redox participation of the PLY ligand coordinated with Ni(ii) results in an excellent catalyst able to perform the regioselective anti-Markovnikov hydrosilylation^11^ of a wide variety of olefins. It might be worth noting that the base metal catalyzed hydrosilylation of alkenes is of great interest and has been pioneered by Chirik and coworkers.3*b*

## Results and discussion

**Scheme 1:**
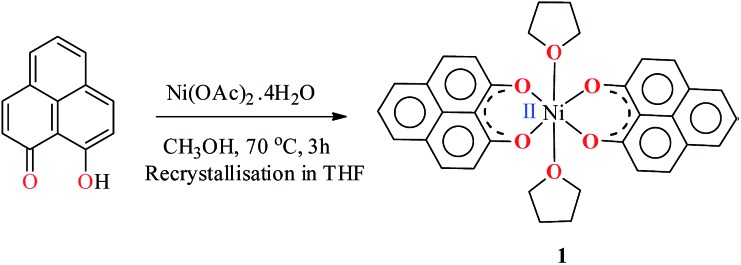
Synthesis of catalyst **1**.

**Fig. 1:**
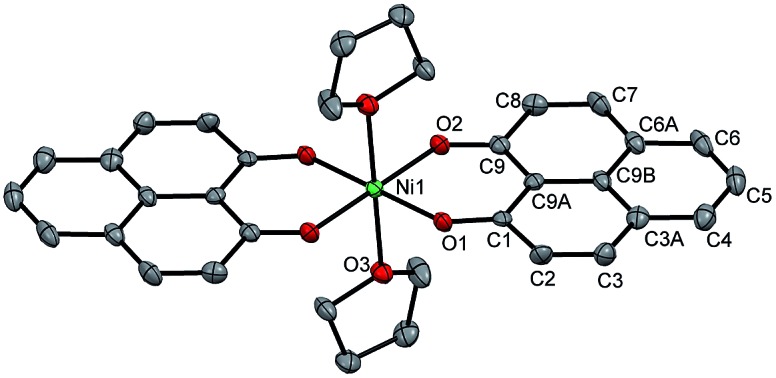
Perspective ORTEP view of the molecular structure (50% ellipsoid level) of **1**, where the hydrogen atoms are omitted for clarity.

**Fig. 2:**
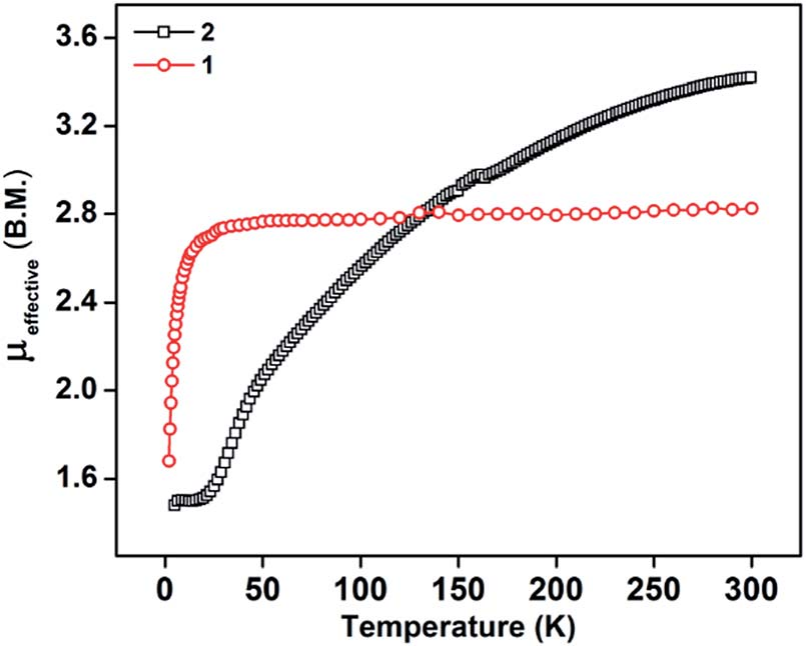
Variation of *μ*_eff_ values as a function of temperature for compound **1**: 2.82 *μ*_B_ at 300 K (before reduction, in red), and **2**: 3.42 *μ*_B_ at 300 K (after reduction, in black), revealing the presence of two unpaired spins in compound **1** and four unpaired spins in compound **2** at rt.

The PLY-nickel(ii) complex, **1** (Scheme 1), was prepared by treating Ni(OAc)_2_·4H_2_O with two equivalents of the ligand, 9-hydroxyphenalenone, in methanol at 60 °C to give a crystalline precipitate. Analytically pure **1** was obtained by recrystallization of the dried reaction mixture in dry THF at 5 °C, yielding dark orange colored crystals of **1** in nearly 90% yield. Complex **1** was paramagnetic, and was characterized by an array of analytical tools including elemental analysis, ESI-MS, IR, and single crystal X-ray diffraction studies. The molecular structure of **1** was determined by single crystal X-ray diffraction, and it shows a tetragonal ligand environment for a six coordinate nickel(ii) ion. The ORTEP diagram (Fig. 1) displays that the Ni^II^ ion is coordinated to four O-donor atoms of the two phenalenone ligands (O1 and O2 atoms as coordination sites), and the axial positions are occupied by two THF ligands. The solid state magnetic susceptibility of **1** was measured revealing a magnetic moment of 2.82 *μ*_B_, fully corroborating with the octahedral Ni^II^ picture (Fig. 2, see later).

**Scheme 2:**
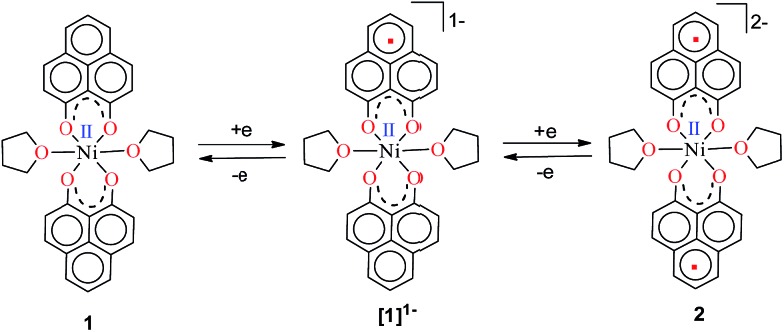
Two successive one electron reductions showing the formation of the phenalenyl-centered anionic radical and di-anionic species, respectively.

Anticipating that PLY may act as a redox storage motif, the electrochemical reduction of **1** was performed using cyclic voltammetry, which revealed two quasi-reversible one-electron waves at –1.26 and –1.52 V (*vs.* SCE, Fig. S1, ESI†). This is indicative of sequential one-electron reductions at the PLY ligand backbone, as inferred previously from the study on a similar Fe(PLY)_3_ system.9*b* This reduction process generates a PLY-based radical and a bi-radical species (Scheme 2), which were also observed in a series of spiro-bis-phenalenyl compounds, reported previously.^12^ Also, the difference between the first reduction potential *E*_1/2_^1^ and the 2^nd^ reduction potential *E*_1/2_^2^ (Δ*E*^2–1^ = 0.26 V) matches very well with earlier electrochemical results.8*a* It may be noted that Δ*E*^2–1^ is considered as the disproportionation potential which is directly correlated with the on-site Coulomb correlation energy (U).8*a* The Δ*E*^2–1^ value is also in close agreement to those of bis-phenalenyl-boron complexes bearing the same *O*,*O*-phenalenyl ligand (Δ*E*^2–1^ values are –0.29 V and –0.28 V), strongly suggesting clear successive two step one-electron reductions into the phenalenyl moiety.8*a* After observing this result, two electrons were chemically injected into **1** by reduction with potassium, yielding a green paramagnetic complex **2** (*vide infra* for its magnetism and electronic structure).

This observation encouraged us to check if such a two-electron reduced species such as **2** can initiate any single electron transfer (SET) process to activate the Si–H bond of silane. Notably, we have very recently used a reduced phenalenyl radical for a SET process in designing transition metal free catalysis for C–H functionalization.^13^ Accordingly, an equimolar mixture of Ph_2_SiH_2_ and 1-octene was examined in the presence of 0.25 mol% of complex **1** and three equiv. of potassium in THF solution under an anaerobic atmosphere. To our great delight, a quantitative conversion to the anti-Markovnikov hydrosilylated product^14^ was observed after 30 minutes at rt (Table 1, entry 2). The hydrosilylation under our chosen condition is very clean and devoid of any other by-product formation resulting from the isomerization, dimerization or hydrogenation of olefins.^11^,^14^ Gratifyingly, we also observed the nearly complete conversion when PhSiH_3_ and Ph_3_SiH were used as silanes (Table 1, entries 1 and 3). However, attempting the reaction with other silanes such as Ph_2_MeSiH, (EtO)_3_SiH, (MeO)_3_SiH or Et_3_SiH led to much lower yields (up to 45%, Table 1, entries 4–7). The control experiments using Ph_2_SiH_2_ as the representative silane clearly established that without catalyst the reaction did not proceed in otherwise identical conditions (Table 1, entry 8). Furthermore, the addition of potassium (sodium also works as a reductant, but yields 70% product) is necessary for this catalytic reaction to proceed (Table 1, entry 9). However, the organic amine tetrakis(dimethylamino)ethylene (TDAE), which is a much milder reducing agent than K or Na, was unable to deliver any product (Table 1, entry 10). It was finally concluded that both the catalyst **1** and a suitable reducing agent were necessary for this catalysis (Table 1, entry 11) since the PLY-based biradical is generated *in situ*. Several earlier reports on nickel catalyzed hydrosilylation also involve the *in situ* generation of the catalytically active species by various external reductants, such as LiAlH_4_, EtMgBr, NaBEt_3_H, *etc.*^15^ A recent report also discloses that nickel catalyzed silane activation and hydrosilylation can be performed across a Ni–Ni bond, utilizing the redox non-innocence of the naphthyridine-diimine ligand.^16^

**Table 1:** Standardization of the catalytic activity with different silanes for the hydrosilylation of 1-octene^*a*^

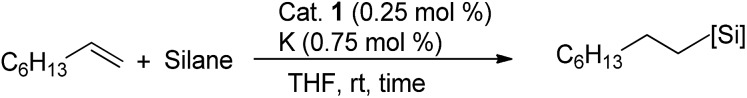				
Entry	Catalyst	Silane	Time (h)	Yield (%)^*b*^
1	**1**	PhSiH_3_	1	99
2	**1**	Ph_2_SiH_2_	0.5	99
3	**1**	Ph_3_SiH	1	95
4	**1**	Ph_2_MeSiH	3	45
5	**1**	(EtO)_3_SiH	3	55
6	**1**	(MeO)_3_SiH	3	20
7	**1**	Et_3_SiH	10	<5
8^*c*^	—	Ph_2_SiH_2_	10	—
9^*d*^	**1**	Ph_2_SiH_2_	10	—
10^*e*^	**1**	Ph_2_SiH_2_	0.5	—
11^*f*^	—	Ph_2_SiH_2_	10	—
12^*d*^	NiCl_2_	Ph_2_SiH_2_	0.5	—
13	NiCl_2_	Ph_2_SiH_2_	0.5	—
14	Ni(acac)_2_	Ph_2_SiH_2_	0.5	<5
15	Ni(acac)_2_(MeOH)_2_	Ph_2_SiH_2_	0.5	<5
16^*g*^	**1**	Ph_2_SiH_2_	0.5	60

^*a*^Typical conditions: catalyst **1** (0.25 mol%), K (0.75 mol%), alkene (1.00 mmol), 1 equiv. silane, THF (1 mL), rt.

^*b*^The reported yields are based on ^1^H NMR spectroscopic measurements.

^*c*^No catalyst **1**.

^*d*^No K.

^*e*^Reaction with TDAE.

^*f*^In the absence of catalyst **1** and K.

^*g*^Catalyst **1** used in 0.05 mol%.

**Chart 1:**
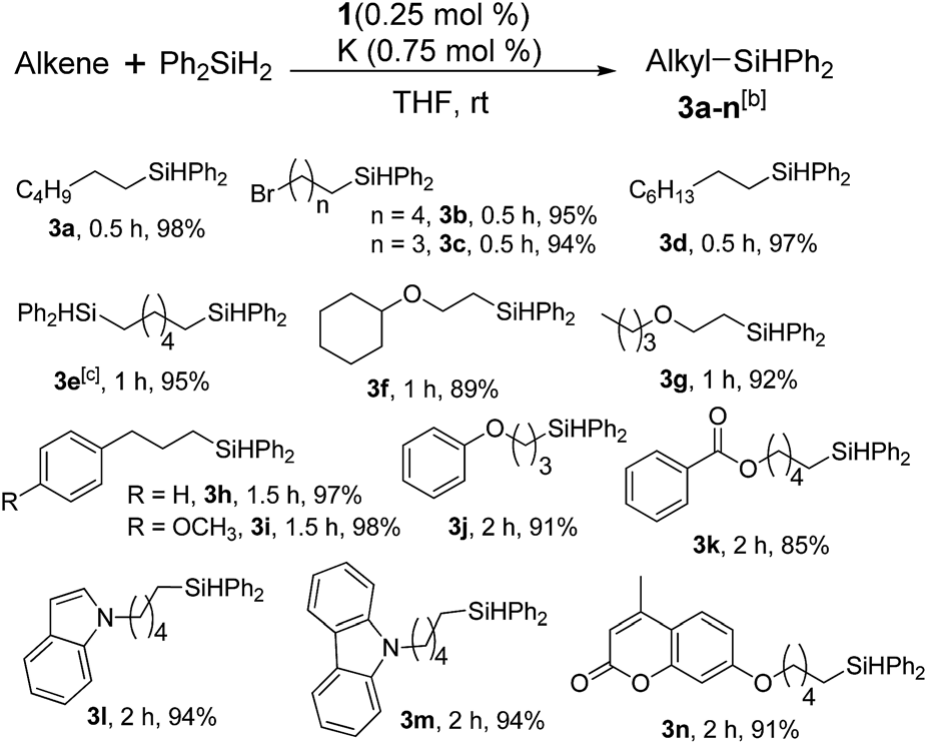
Nickel-catalyzed hydrosilylation of terminal alkenes^a^. ^a^Reaction conditions: alkene (0.5 mmol), Ph_2_SiH_2_ (1 equiv.), **1** (0.25 mol%), K (0.75 mol%), THF (1 mL), rt. ^b^Yields are reported after purification by column chromatography. ^c^2 equiv. of Ph_2_SiH_2_ added.

Furthermore, to establish that the PLY-based nickel complex is essential for the reaction, we performed catalysis with only NiCl_2_ as well as a NiCl_2_/K combination, both of which did not result in any hydrosilylated product (Table 1, entries 12 and 13) under identical conditions to those used for catalyst **1**. To substantiate the crucial role of the PLY ligand as a redox reservoir during the catalysis, we further conducted another control experiment with the Ni(acac)_2_ or Ni(acac)_2_(MeOH)_2_ (acac = acetylacetonate) complexes,^17^ assuming that the coordination environment of Ni in these complexes closely mimics the coordination environment of catalyst **1**. The hydrosilylation performed with a Ni(acac)_2_/K or Ni(acac)_2_(MeOH)_2_/K combination resulted in an extremely small amount of product (below 5%, Table 1, entries 14 and 15) under identical conditions (opposed to 99% for the **1**/K combination, Table 1, entry 2). This result provides significant support to the hypothesis that the redox active PLY based ligand plays an important role as an electron reservoir, which facilitates redox chemistry upon reduction with K metal. Moreover, the efficacy of our catalyst is impressive, since it can even operate at a minuscule loading (0.05 mol%, Table 1, entry 16), translating into a turnover number of 1200. With this initial result in hand, we investigated the substrate scope further for functionalized olefins using Ph_2_SiH_2_ as the silane source. As summarized in Chart 1, 1-hexene, 5-bromo-1-pentene and 4-bromo-1-butene show essentially complete conversion to the corresponding organosilanes (**3a**, **3b**, and **3c**, respectively) within 30 min at rt. The formation of the anti-Markovnikov product was observed exclusively in all cases. Interestingly, the hydrosilylation of an unstrained internal olefin, 2-octene, also took place smoothly to deliver only the terminally silylated product (**3d**) in 97% yield, most likely *via* successive olefin isomerization and hydrosilylation. Moreover, the hydrosilylation of 1,5-hexadiene with 2 equiv. of Ph_2_SiH_2_ successfully resulted in the disilylated product 1,6-bis(diphenylsilyl)hexane (**3e**). This is a clear improvement, since the hydrosilylation of dialkenes such as 1,5-hexadiene has earlier been very problematic.^18^ The versatility of the substrate scope was further evidenced by the successful silylation of vinyl cyclohexyl ether, vinyl butyl ether and phenyl allyl ether, which were quantitatively converted to the corresponding anti-Markovnikov products (**3f**, **3g** and **3j**, respectively) within two hours. Excellent functional group tolerance was also achieved with anti-Markovnikov selectivity, as tested with a group of various aromatic substrates. Allylbenzene and 4-methoxy allylbenzene can be completely converted to the corresponding linear products (**3h** and **3i**) within 1.5 hours. Further experiments exhibited that the presence of esters and tertiary amines is well tolerated, providing the expected chemoselective hydrosilylated products (**3k–3n**) in excellent isolated yields.

Inspired by these results, we became interested in further exploring the scope of this methodology for the double hydrosilylation method. Usually, the double alkylation of a silane to produce a dialkylated product by adding one more equivalent of alkene in the same reaction mixture is potentially problematic.^19^ In our case, the hydrosilylation of a 2 : 1 molar ratio of alkenes with RSiH_3_ (R = Ph, Bu) yielded the corresponding dialkyl silylated products R(alkyl)_2_SiH exclusively (Table 2, entries 1–4). However, the dialkylated silane product remained inactive toward further hydrosilylation. We have also studied the double alkylation of hydrosilanes with one equiv. RSiH_3_ and the stepwise addition of two different alkenes (Table 2, entries 5–10). For example, (5-bromopentyl)(octyl)(phenyl)silane was obtained in 89% yield by the sequential addition of 1-octene and 5-bromopentene (Table 2, entry 5) in a single pot. We believe that such smooth silylation reactions under very mild conditions likely result from the redox tuning between the PLY and Ni^II^ orbitals.

**Table 2:** Double alkylation reaction of alkenes with RSiH_3_ (R = Ph, Bu)^*a*^

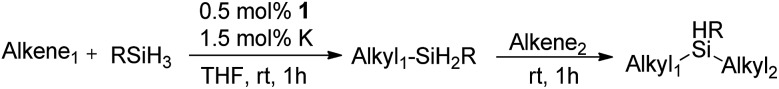					
Entry	Alkene_1_	R	Alkene_2_	Product	Yield (%)^*b*^
1	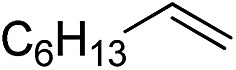	Ph	—	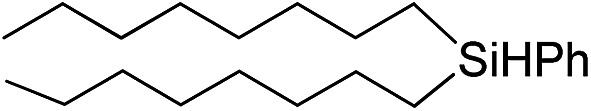	96
2	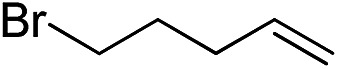	Ph	—	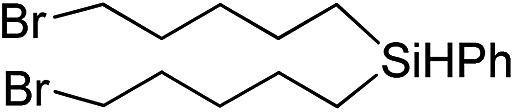	95
3^*c*^	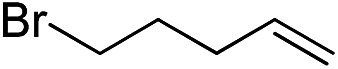	Bu	—	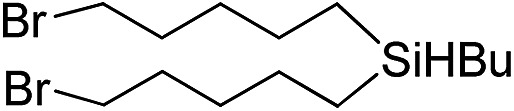	97
4^*c*^	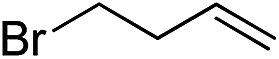	Bu	—	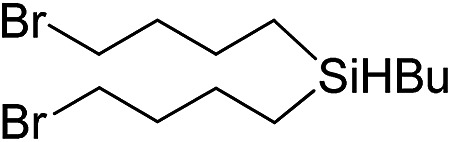	97
5	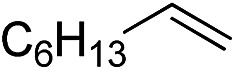	Ph	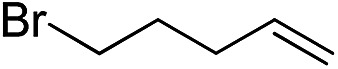	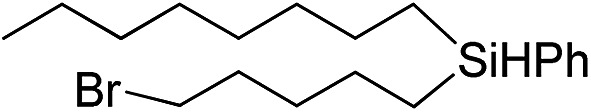	89
6	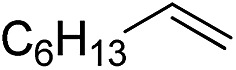	Ph	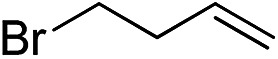	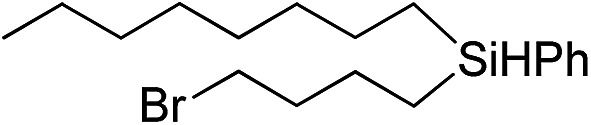	92
7^*c*^	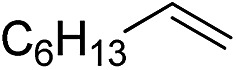	Bu	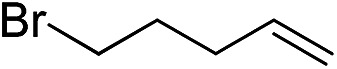	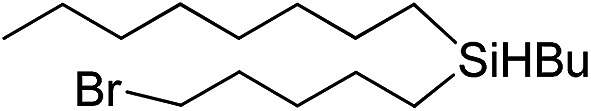	93
8^*c*^	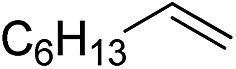	Bu	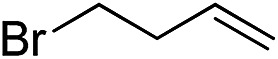	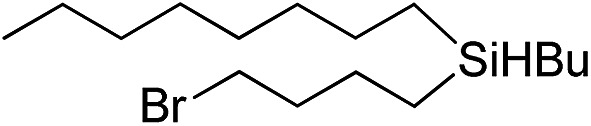	91
9	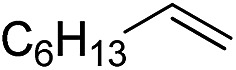	Ph	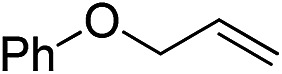	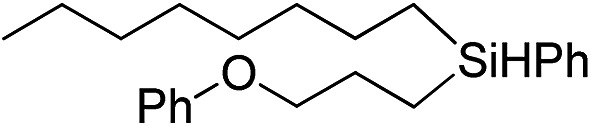	85
10	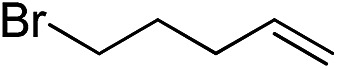	Ph	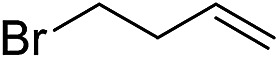	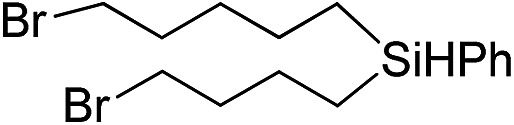	92

^*a*^Reaction conditions: **1** (0.5 mol%), K (1.5 mol%), alkene_1_ (0.5 mmol), RSiH_3_ (1 equiv.), alkene_2_ (0.5 mmol), THF (1 mL), rt.

^*b*^Isolated yields after purification by column chromatography.

^*c*^ *t* = 6 h.

**Scheme 3:**
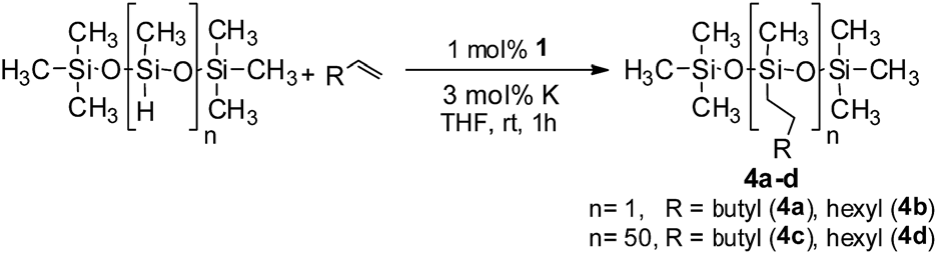
The hydrosilylation of olefins by siloxanes.

In the context of synthesizing silicones, the alkylation of polymethylhydrosiloxane (PMHS) is one of the most common chemical methods. A large variety of catalysts, mostly comprising expensive platinum, have been studied for the modification of siloxanes.^20^ To the best of our knowledge, there is no efficient nickel based catalyst for the hydrosilylation of alkenes with PMHS as a precursor silane. This fact prompted us to examine the competency of **1** towards hydrosilylation using PMHS. The model hydrosilylations of different alkenes (1-octene and 1-hexene) using heptamethyltrisiloxane yielded the anti-Markovnikov addition products hexyl-1,1,1,3,5,5,5- and octyl-1,1,1,3,5,5,5-heptamethyltrisiloxane (**4a** and **4b**, respectively) in 79% and 84% isolated yields (Scheme 3). It may be noted that the product **4a** is a commercially available agricultural adjuvant as well as a sensory and performance enhancer in cosmetic formulations.11*b* Next, we carried out the hydrosilylation of 1-octene and 1-hexene with PMHS containing approximately 50 Si–H units, resulting in the corresponding hydrosilylated products (**4c** and **4d**). These are important intermediates towards the functionalization of silicones,^14^,^20^ and were obtained in nearly quantitative yield.

To understand the mechanistic pathway for this fascinating reaction, we first carried out the catalysis by adding 1 equiv. K with respect to **1**, which resulted in the reaction not proceeding at all. Then, we checked whether **2** (the two electron reduced product of **1**, see the drawing in Scheme 2) could act as an active catalyst for the hydrosilylation reaction. We isolated the radical **2** as a green solid in bulk by performing the reduction of **1** with K metal in dry THF strictly under a dry nitrogen atmosphere. After isolation, **2** was dried and stored in a nitrogen filled glovebox. The reaction of 1-octene or allylbenzene with Ph_2_SiH_2_ in the presence of a catalytic amount of complex **2** (0.25 mol%) in THF afforded the corresponding anti-Markovnikov products in 98% and 95% yields, almost identical to the yields *via* the *in situ* catalytic protocol (99%, Table 1, entry 2, and 97%, Chart 1, **3h**). The bulk isolation and storage of compound **2** ensures that the catalysis can be accomplished without involving K metal in every catalytic run.

**Scheme 4:**
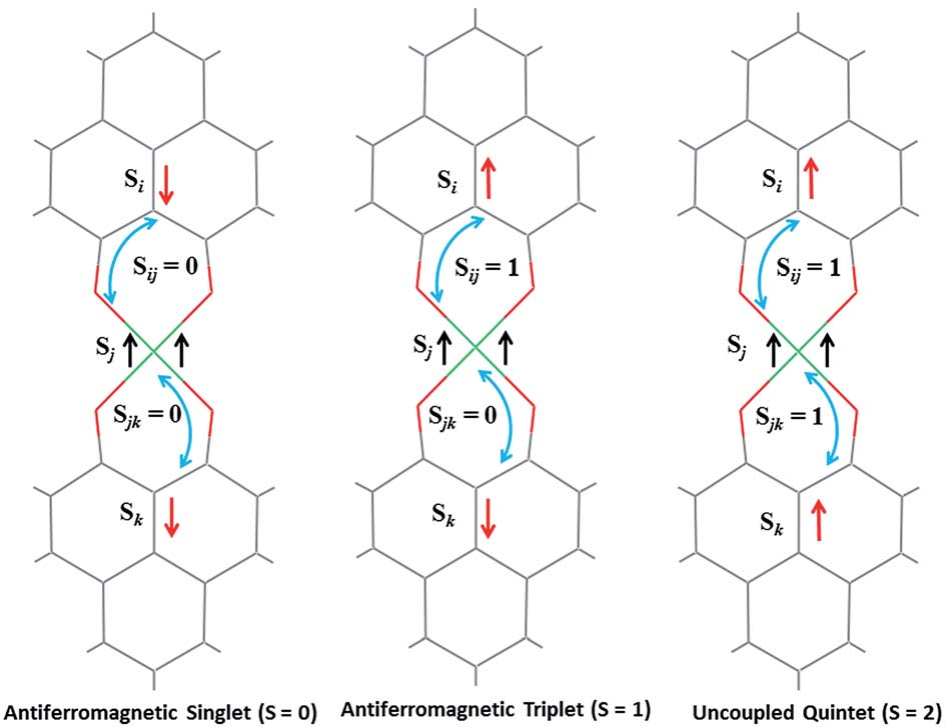
Schematic representation of possible electronic spin states of compound **2**, where two ligand based electrons are interacting with the high spin Ni^II^.

The direct role of **2** in the hydrosilylation catalysis prompted us to investigate the electronic structure of **2** in the light of the redox non-innocence of the PLY backbone. Since our electrochemical experiments revealed that the reductions of **1** are predominantly ligand based (*vide supra* and references therein),^9^,^10^ three spin states may be envisaged for **2** (Scheme 4). Upon two electron reduction to **1**, it may either generate a fully AF-coupled singlet (diamagnetic), or an AF-coupled triplet or a quintet state (Scheme 4). Among these choices, the first one is easily discarded since **2** is paramagnetic (magnetic data shown in Fig. 2). To investigate this spin ground state of **2** with respect to the second and third choices, we analyzed a solid state magnetometry data using SQUID, varying the temperature from 300 to 2 K (Fig. 2). This analysis discloses that the magnetic moment of **2** is 3.42 *μ*_B_ at rt, and decreases smoothly to reach a value of 1.52 *μ*_B_ at 20 K. This sharp drop of the magnetic moment with the lowering of the temperature is a clear signature of AF-coupling.^21^ The *μ*_eff_ at rt is smaller than that expected for a *S* = 2 system, however it is very close to 3.44 *μ*_B_, a spin-only value anticipated for a ground state consisting of isoenergetic *S* = 1 and *S* = 2 states. To arrive at this value, the general formula of *μ*_total_ = [(*μ*_A_)^2^ + (*μ*_B_)^2^]^1/2^ is used when two isolated spin ground states A and B are degenerate.^22^ In our case, the calculated magnetic moment becomes [3/8 × (2.83)^2^ + 5/8 × (3.74)^2^]^1/2^ = 3.44 *μ*_B_, considering the spin only values for the states (*S* = 1 and *S* = 2) and factoring their respective degeneracies. The experimentally observed value is very close to the theoretically projected value, which strongly supports the near degeneracy of the two spin states. Furthermore, as will be reported below, DFT calculations suggest that the AF-coupled triplet and the quintet states are indeed iso-energetic. Our conclusion from the SQUID experiment is that **2** possesses an AF-coupled triplet (*vide infra*) as the ground state, although a quintet state is highly accessible over a wide temperature range. The paramagnetic **2** is EPR silent (X-band) at both room and low temperature, which is hardly surprising for a non-Kramers *S* = 1 spin system.^23^ To find a definite spectroscopic signature for the proposed AF-coupled triplet ground state of **2**, we attempted to find the spin forbidden (Δ*S* = ±2) transition at the half-field strength of the applied magnetic field, by performing parallel mode EPR spectroscopy in dry DMSO at 5 K. Unfortunately, the signature of such a half-field signal was not observed, which may be attributed to the high zero field splitting associated with the Ni(ii) ion.^24^ Both the CV and SQUID magnetometry data clearly preclude the possibility of the metal-centered reduction leading to diamagnetic Ni^0^ (*vide infra* for the electronic structure of **2**). We scrutinized this fact further by using X-ray photoelectron spectroscopy (XPS) for both **1** and **2** (Fig. S2, ESI†). The XPS data displays that for **2**, the 2P_3/2_ transition occurs at 854.7 eV along with a characteristic satellite peak at 860.2 eV. This peak corresponds to Ni(ii), and remains the same in both **1** and **2**.^25^ The identical peak position for the complexes before and after reduction supports that the oxidation state of nickel is completely retained upon reduction.

**Fig. 3:**
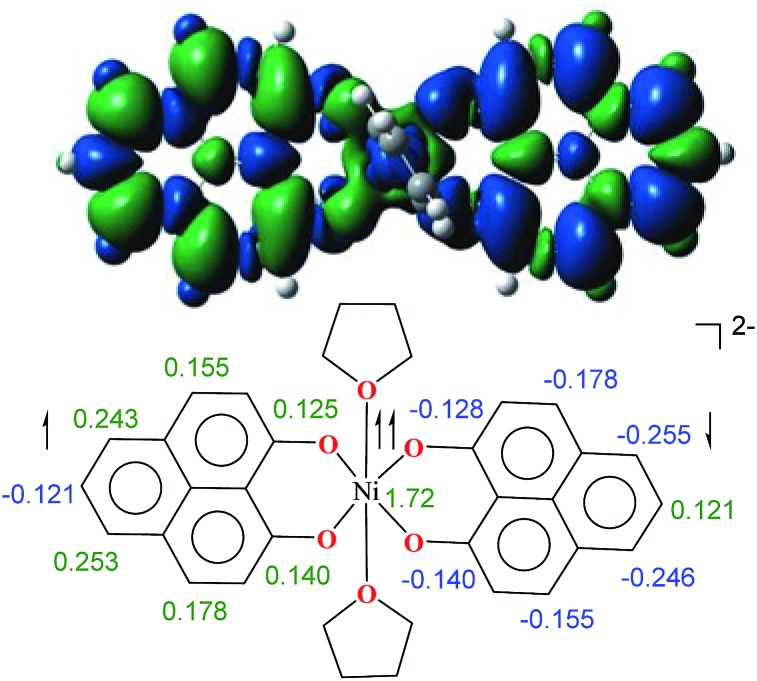
The spin density plot for **2**, where the colour coding refers to green = excess α spin and blue = excess β spin. The contour values were set to ±0.0004 (e bohr^–3^)^1/2^ (above). The major numerical values of excess spin densities are shown (below).

To further shed light on the details of the electronic structure of **2** we resorted to high-level DFT calculations. The analysis of the molecular orbitals exposes that the LUMOs for both **1** (Fig. S3a, ESI†) and its one-electron reduced product (Fig. S3b, ESI†) are completely ligand localized. This fact strongly suggests that the PLY ligand can accept and hold an electron (Fig. S5, ESI† for the spin density in the single-electron reduced product) so that the oxidation state of nickel remains constant as Ni^II^ during the reduction processes. Indeed, at the B3LYP/6-31+G* (lanl2dz pseudopotential for Ni) level of calculations, we found that the two-electron reduced product **2** possesses an antiferromagnetically (AF) coupled triplet as the ground state with a very low-lying excited quintet state. The <*S*^2^> value obtained for the AF-coupled triplet electronic structure was 2.11, which reflects a slight amount of spin contamination and thus lends credence to the ground state energy value. As we have discussed earlier, this electronic structure is fully consistent with the SQUID results. The broken symmetry solution^26^ for an AF-coupled triplet is clearly depicted by the spin density plot of **2** (Fig. 3). This proves that the electrons donated during the reductions are housed by the two PLY frameworks. One of the ligand-based electrons is likely AF-coupled with a nickel-centric electron, which results in two unpaired electrons distributed over the nickel and other PLY ligand, overall resulting in a triplet ground state. This proposition does not preclude other weak exchange coupling schemes to arrive at the AF-coupled singlet state though. Additionally, electron donation to the ligand-centric low lying LUMO can easily be traced by the respective C–C bond length changes of the reduced ligand backbone (Fig. S4, ESI†). Moreover, the natural population analysis performed on the symmetry broken densities of **2** exposes that the 3d orbital population is 8.2, which further proves the presence of Ni^II^.^27^

**Scheme 5:**
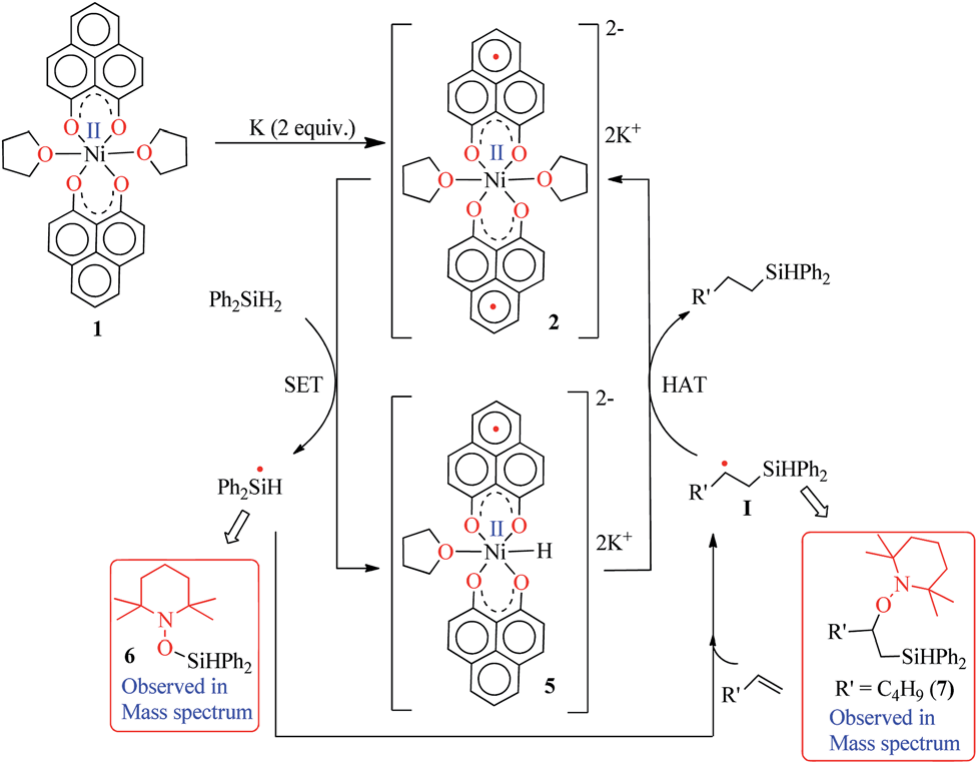
A plausible mechanism for the catalytic hydrosilylation of olefin by **1** through PLY based radical initiation.

Keeping this electronic picture of **2** in mind, we anticipated that the olefin hydrosilylation may follow a radical pathway. To check this assertion further, the radical scavenger TEMPO was added (two runs with 1 and 2 equiv.) to the reaction mixture, which drastically reduced the yield of the hydrosilylated product to 43% and 15%, respectively (see Scheme S1, ESI†). The same radical quenching was also performed using the galvinoxyl radical (using two equiv.), which fully restricted the reaction (see Scheme S2, ESI†). Based on these preliminary findings, a plausible reaction mechanism has been proposed involving a radical pathway (Scheme 5). A single electron transfer (SET) occurs from **2** to the silane to generate a silyl radical which subsequently adds to the olefin, resulting in the formation of a silylated alkyl radical (**I**). The generation of a silyl radical during this hydrosilylation has been unambiguously authenticated by trapping the TEMPO-adduct of the putative radical, **6**. The radical mechanism is also in agreement with our recent report, where a K-complex of PLY can participate in the catalytic SET process to accomplish transition metal free C–C coupling catalysis.^13^ Upon SET, **2** transforms into an anionic monoradical nickel hydride containing species, **5**. The silylated alkyl radical **I** subsequently reacts with a hydrogen atom which originates from the nickel hydride species **5**. This hydrogen atom transfer (HAT) from the metal hydride to result in a silylated alkane closely resembles many transition metal hydride promoted hydrogenations of olefins where a radical is involved. Some classic studies by Halpern and Norton have shown that the hydrogenation of anthracene and styrenes using HMn(CO)_5_ generates a carbon based radical *via* HAT.^28^ Encouragingly, the intermediacy of **I** has also been proven by trapping the TEMPO-adduct, **7**, as observed using mass spectrometry. The anti-Markovnikov selectivity in our hydrosilylation reaction is fully anticipated, since the silyl radical addition to the olefin will only engender a stable secondary carbon radical in contrast to a primary one. During the HAT to **I** which furnishes the final product, one electron returns to the catalyst and is housed in the ligand backbone, thus regenerating **2**. Notably, this type of silyl radical mediated hydrosilylation of an alkene has been proposed in several earlier reports.^29^ Moreover, the ligand-based redox promoting homolytic cleavage of an X–H bond has recently been reported, which provides further support to our mechanistic postulate.^30^ Our proposed mechanistic hypothesis is clearly different from the conventional Chalk–Harrod^31^ or modified Chalk–Harrod mechanisms^32^ of hydrosilylation, which are often operative in precious metal based catalysis. It also differs from Chirik’s recent report, where the oxidative addition of (EtO)_3_Si–H happens at the nickel center which is supported by the presence of a redox non-innocent aryl-substituted α-diimine ligand.^33^

## Conclusions

In summary, using the capability of PLY ligand to store redox equivalent, we have been able to synthesize a very effective nickel-assisted catalytic system to carry out olefin hydrosilylation under mild conditions. The ligand centered radicals upon reduction of **1** made this catalysis feasible. It may be noted that although PLY centered radicals have been well-known for over five decades, their use in electron transfer catalysis is an evolving area, and this study establishes that there is an enormously promising future in the design of base metal-assisted PLY based radicals for the development of catalysts for numerous organic transformations. Our current focus resides on developing more base metal catalysts using ligand based electrons and understanding the fascinating electronic structure of the exchange-coupled systems which are responsible for delivering catalysis.

## Experimental section

### General considerations

All manipulations were performed under a dry and oxygen free atmosphere (argon) using standard Schlenk techniques or inside a glovebox maintained below a 0.1 ppm concentration of O_2_ and H_2_O. Prior to use, all glassware were oven-dried (130 °C) and evacuated while hot. All solvents were distilled from Na/benzophenone prior to use. All other chemicals were purchased from Sigma–Aldrich and used as received. The HRMS data were obtained using a Finnigan MAT 8230 instrument. The ESI-MS data were obtained using a maXis impact™ mass spectrometer. Elemental analysis was carried out using a PerkinElmer 2400 CHN analyzer, and sample was prepared by keeping them under reduced pressure (10^–2^ mbar) overnight. The melting points were measured in a sealed glass tube on a Büchi B-540 melting point apparatus. Analytical TLC was performed on a Merck 60 F254 silica gel plate (0.25 mm thickness). ^1^H, ^13^C and ^29^Si NMR spectra were recorded on a JEOL ECS400 MHz spectrometer and on a Bruker Avance III 500 MHz spectrometer. EPR spectra were recorded on a JEOL JES-FA200 X-band spectrometer. All chemical shifts were reported in ppm using tetramethylsilane as a reference. Electrochemical analysis was performed using a three-electrode system on a Princeton Applied Research 263A electrochemical workstation. CV measurements were carried out in THF using a Pt working electrode, Pt-wire as the counter electrode and a SCE as the reference electrode. TBAPF_6_ (0.1 M in dry THF) was utilized as the supporting electrolyte. Electrochemical analysis was carried out in an inert atmosphere. Phenalenyl (PLY) ligand was prepared according to the literature procedure.^34^

### Procedure for the synthesis of **1**

In a 100 mL round bottom flask, PLY (0.98 g, 5 mmol) was dissolved in 60 mL MeOH upon heating at 60 °C. Separately, a 100 mL conical flask was taken and Ni(OAc)_2_·4H_2_O (0.62 g, 2.5 mmol) was dissolved in 30 mL MeOH. To the hot methanolic solution of the PLY ligand, the solution of Ni(OAc)_2_ dissolved in methanol was added dropwise. The yellow solution turned darker and slowly a bright orange precipitate started forming. The reaction mixture was stirred vigorously with heating at 60 °C for another 3 h, and then the reaction mixture was allowed to cool at rt. Subsequently the precipitate was filtered. The precipitate was washed repeatedly with MeOH to remove any unreacted ligand and metal salt. The title compound was recrystallized from THF. Crystals suitable for SCXRD were grown by dissolving the recrystallized material in dry THF and keeping it at 4 °C in a Schlenk flask for 1–2 weeks. Yield 90%. UV-visible (THF) *λ* max per nm (*ε* in M^–1^ cm^–1^): 288 (10 931), 351 (38 000), 413 (9220), 436 (17 542), 463 (16 867). FT-IR (thin film) *ν*(cm^–1^): 3695.15, 2693.20, 1627.97, 1518.88, 1433.84, 1345.45, 1260.30, 1095.77, 901.58, 749.65. ESI-MS: *m*/*z* calc. for C_34_H_31_NiO_6_ [M + H]^+^ 593.1474, found 593.1468. Elemental analysis: anal. calcd for C_34_H_30_NiO_6_: C, 68.83; H, 5.10. Found: C, 68.88; H, 5.17.

### General procedure for the hydrosilylation of functionalized alkenes with catalyst **1**

To a stirred solution of **1** (1.5 mg. 0.0024 mmol) and K (0.30 mg, 0.0074 mmol) in THF (1 mL), the silane (1 mmol) and alkene (1.00 mmol) were added at room temperature. The solution was stirred at room temperature, and the progress of the reaction was monitored using ^1^H NMR spectroscopy. After completion of the reaction, hexamethyl benzene (120 mg, 1.00 mmol) was added as an internal standard to the reaction mixture. The ^1^H NMR analysis of the resulting solution revealed the formation of the product. The solution was concentrated under vacuum, and the residue was purified by column chromatography using hexane as an eluent. The final product was characterized using ^1^H, ^13^C and ^29^Si NMR spectroscopic studies.

### General procedures for double alkylation of alkenes with PhSiH_3_

To a stirred solution of **1** (1.5 mg, 0.0024 mmol) and K (0.3 mg, 0.0075 mmol) in THF (1 mL), silane (0.5 mmol) and alkene (0.5 mmol) were added at room temperature and the solution was stirred at room temperature. After 1 h, another 0.5 mmol of alkene was added to the reaction mixture and stirred at room temperature for 1 h. The progress of the reaction was monitored using ^1^H NMR spectroscopy. After completion of the reaction, hexamethyl benzene (60 mg, 0.50 mmol) was added as an internal standard to the reaction mixture. The ^1^H NMR spectroscopic analysis of the resulting solution revealed the formation of the product. The solution was concentrated under vacuum, and the residue was purified by column chromatography using hexane as an eluent. The final product was characterized using ^1^H, ^13^C and ^29^Si NMR spectroscopy.

### Procedure for the hydrosilylation of alkenes with 1,1,1,3,5,5,5-heptamethyltrisiloxane (HMTS)

To a stirred solution of **1** (2.96 mg, 0.0049 mmol) and K (0.6 mg, 0.015 mmol) in THF (1 mL), HMTS (0.5 mmol) and alkene (0.5 mmol) were added at room temperature. The solution was stirred at room temperature. The progress of the reaction was monitored using ^1^H NMR spectroscopy. After completion of the reaction, hexamethyl benzene (0.50 mmol) was added as an internal standard to the reaction mixture. The ^1^H NMR spectroscopic analysis of the resulting solution revealed the formation of the product. The solution was concentrated under vacuum, and the residue was purified by column chromatography using hexane as an eluent. The final product was characterized using ^1^H, ^13^C and ^29^Si NMR spectroscopic studies.

### Procedure for the hydrosilylation of alkenes with polymethyl hydrosiloxane (PMHS)

To a stirred solution of **1** (2.96 mg, 0.0049 mmol) and K (0.6 mg, 0.015 mmol) in THF (1 mL), PMHS (0.5 mmol) and alkene (50 equiv.) were added at room temperature. The solution was stirred at room temperature. The progress of the reaction was monitored using ^1^H NMR spectroscopy. After completion of the reaction, hexamethyl benzene (0.50 mmol) was added as an internal standard to the reaction mixture. The ^1^H NMR spectroscopic analysis of the resulting solution revealed the formation of the product. The solution was concentrated under vacuum, and the residue was purified by column chromatography using hexane as an eluent. The final product was characterized using ^1^H and ^13^C NMR spectroscopic studies.

### Procedure for the preparation of compound **2**

**1** and potassium were mixed in a 1 : 3 ratio using dry THF as the solvent. The resulting mixture was allowed to stir for 10 min at room temperature, when a green precipitate was formed. The solvent was evaporated under high vacuum when **2** was obtained as a green solid. The spin state of **2** was characterized using SQUID, nickel oxidation state was scrutinized by X-ray photoelectron spectroscopy, and further electronic structural detail was calculated using DFT. All our attempts to collect elemental analysis data or mass spectroscopic data of the radical **2** did not succeed, which may be attributed to its high sensitivity towards air and moisture.

### Computational details

All calculations were carried out using Density Functional Theory as implemented in the Gaussian 09 35 quantum chemistry programs. The geometries of the stationary points were optimized with the generalized gradient approximation (GGA) by means of the Becke exchange functional along with Lee, Yang, and Parr correlation functional (LYP). We used a double-ζ basis set with the relativistic effective core potential of Hay and Wadt (LANL2DZ) for the nickel atom and 6-31+G(d) basis set for the other elements (H, C, and O). The geometries were optimized without any symmetry constraints. For the optimization, a full model was chosen with furan as the weakly coordinating ligand. The symmetry broken DFT solution was detected using the Gaussian keyword stable = opt. Harmonic force constants were computed at the optimized geometries to characterize the stationary points as minima. The molecular orbitals were visualized and the spin density was plotted using Gaussview.

### X-ray crystallographic details

A single crystal of compound **1** was mounted on a glass tip. Intensity data were collected on a SuperNova, Dual, Mo at zero, Eos diffractometer. The crystal was kept at 100 K during the data collection. The atomic coordinates, the isotropic and anisotropic displacement parameters of all the non-hydrogen atoms were refined using Olex2,^36^ and the structure was solved with the Superflip^37^ structure solution program using Charge Flipping and refined with the ShelXL^38^ refinement package using Least Squares minimization.

### SQUID details

A SQUID magnetometer (Quantum Design MPMS) was used to investigate the magnetic properties (magnetic susceptibility) of the compounds. We have used a lightweight homogeneous quartz tube as a sample holder for the magnetic measurements in SQUID MPMS-XL5 to minimize the background noise and stray field effects. The magnetic data were corrected for the diamagnetic contribution from the sample holder by measuring the magnetic moment of the sample holder with an air gap corresponding to the sample length. The intrinsic diamagnetism of the samples was corrected by the standard literature using Pascal’s constants.

## Conflicts of interest

There are no conflicts to declare.

## Supplementary Material

Supplementary information

Crystal structure data
