# Supplementary material for: Tuning the redox non-innocence of a phenalenyl ligand toward efficient nickel-assisted catalytic hydrosilylation
Source: Chem Sci. 2018 Jan 31;9(10):2817–25. doi: 10.1039/c7sc04687a (PMC5914464; doi:10.1039/c7sc04687a)
Supplement: Supplementary file 1 [file SC-009-C7SC04687A-s001.pdf]

Electronic Supplementary Material (ESI) for Chemical Science.
This journal is © The Royal Society of Chemistry 2018

Electronic Supplementary Information

Tuning redox non-innocence of phenalenyl ligand toward efficient nickel-assisted catalytic hydrosilylation

Gonela Vijaykumar,[a] Anand Pariyar,[a] Jasimuddin Ahmed,[a] Bikash Kumar Shaw,[a] Debashis Adhikari,[b] Swadhin K. Mandal*[a]*

[a]Department of Chemical Sciences, Indian Institute of Science Education and Research Kolkata, Mohanpur 741246, India
[b]Department of Chemical Sciences, Indian Institute of Science Education and Research Mohali, SAS Nagar 140306, India

E-mail: adhikari@iisermohali.ac.in, swadhin.mandal@iiserkol.ac.in

Contents:

1. ^{1}H, ^{13}C and ^{29}Si NMR spectroscopic data of hydrosilylation products with Ph_2SiH_2......................

2. ^{1}H, ^{13}C and ^{29}Si NMR spectroscopic data of double hydrosilylation products with $PhSiH_3$………....

3. ^{1}H, ^{13}C and ^{29}Si NMR spectroscopic data of hydrosilylation products with HMTS and PMHS.........

4. Procedure for reaction inhibition in presence of radical scavenger TEMPO……………………..…..

5. Procedure for reaction inhibition in presence of galvinoxyl radical………………………………………

6. Cyclic Voltammogram of complex **1**………………………………………..………..…………...…

7. Magneto-structural Correlation……………………………………………………………………

8. X-ray photoelectron spectroscopy (XPS)……………………………………………………………..

9. Computational results……………………………………..…………………..………..…

10. ^{1}H, ^{13}C and ^{29}Si NMR spectra……………………………………………………..……

11. Radical trapping by TEMPO and detection by mass spectroscopy…………………..……..….

12. X-ray crystallographic details……………………………………………………………………

13. Computational details……………………………………………………………………………

14. References……………………………………………………………………………...………..

1. Spectroscopic data of products in catalytic hydrosilylation with Ph_2SiH_2.

Octylphenylsilane[1]

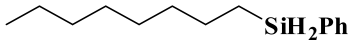

^{1}H NMR (500 MHz, $CDCl_3$, 25 °C): δ 7.57-7.59 (m, 2H), 7.35-7.42 (m, 3H), 4.30 (t, 2H, J = 3.4 Hz), 1.27-1.50 (m, 12H), 0.88-0.98 (m, 5H) ppm. ^{13}C NMR (100 MHz, $CDCl_3$, 25 °C): δ 135.3, 132.9, 129.6, 128.1, 33.0, 32.0, 29.4, 29.3, 25.2, 22.8, 14.3, 10.2, ppm. ^{29}Si NMR (79.3 MHz, $CDCl_3$, 25 °C): δ -30.8 ppm.

Octyldiphenylsilane[1]

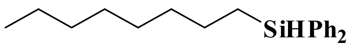

^{1}H NMR (500 MHz, $CDCl_3$, 25 °C): δ 7.58 (dt, J = 9.1, 3.4 Hz, 4H), 7.43 – 7.34 (m, 6H), 4.89 (t, J = 3.7 Hz, 1H), 1.52 – 1.45 (m, 2H), 1.42 – 1.35 (m, 2H), 1.34 – 1.24 (m, 8H), 1.21 – 1.14 (m, 2H), 0.90 (t, J = 7.0 Hz, 3H) ppm. ^{13}C NMR (100 MHz, $CDCl_3$, 25 °C): δ 135.1, 134.7, 129.4, 127.9, 33.2, 31.9, 29.2, 29.2, 24.4, 22.6, 14.1, 12.1 ppm. ^{29}Si NMR (79.3 MHz, $CDCl_3$, 25 °C): δ -13.8 ppm.

Hexyldiphenylsilane[1]

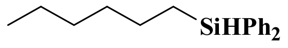

^{1}H NMR (500 MHz, $CDCl_3$, 25 °C): δ 7.59 – 7.56 (m, 4H), 7.41 – 7.35 (m, 6H), 4.87 (t, J = 3.7 Hz, 1H), 1.51 – 1.43 (m, 2H), 1.39 (dq, J = 14.0, 7.0 Hz, 2H), 1.31 – 1.25 (m, 4H), 1.19 – 1.14 (m, 2H), 0.88 (t, J = 6.5 Hz, 3H) ppm. ^{13}C NMR (100 MHz, $CDCl_3$, 25 °C): δ 135.1, 134.7, 129.4, 129.9, 32.8, 31.4, 24.4, 22.5, 14.1, 12.1 ppm. ^{29}Si NMR (79.3 MHz, $CDCl_3$, 25 °C): δ -13.8 ppm.

(5-Bromopentyl)diphenylsilane

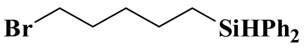

^{1}H NMR (500 MHz, $CDCl_3$, 25 °C): δ 7.58 (ddd, J = 5.2, 3.6, 2.0 Hz, 4H), 7.42 – 7.36 (m, 6H), 4.89 (t, J = 3.7 Hz, 1H), 3.38 (t, J = 6.9 Hz, 2H), 1.90 – 1.83 (m, 2H), 1.56 – 1.48 (m, 4H), 1.21 – 1.14 (m, 2H) ppm. ^{13}C NMR (100 MHz, $CDCl_3$, 25 °C): δ 135.1, 134.3, 129.6, 128.0, 33.7, 32.4, 31.6, 23.6, 12.0 ppm. ^{29}Si NMR (79.3 MHz, $CDCl_3$, 25 °C): δ -13.9 ppm. ESI-MS: m/z calc. for $C_{17}H_{22}BrSi$ $[M+H]^+$ 333.0674, found 333.0678.

(4-Bromobutyl)diphenylsilane

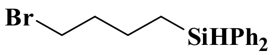

^{1}H NMR (500 MHz, $CDCl_3$, 25 °C): δ 7.59 (dt, J = 6.1, 2.1 Hz, 4H), 7.44 – 7.37 (m, 6H), 4.91 (t, J = 3.7 Hz, 1H), 3.42 (t, J = 6.8 Hz, 2H), 2.00 – 1.91 (m, 2H), 1.69 – 1.59 (m, 2H), 1.23 – 1.15 (m, 2H) ppm. ^{13}C NMR (100 MHz, $CDCl_3$, 25 °C): δ 135.1, 134.1, 129.6, 128.0, 35.8, 33.2, 23.0, 11.3 ppm. ^{29}Si NMR (79.3 MHz, $CDCl_3$, 25 °C): δ -14.1 ppm. ESI-MS: m/z calc. for $C_{16}H_{20}BrSi$ $[M+H]^+$ 319.0517, found 319.0513.

1,6-Bis(diphenylsilyl)hexane

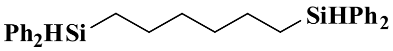

^{1}H NMR (500 MHz, $CDCl_3$, 25 °C): δ 7.62 – 7.51 (m, 8H), 7.43 – 7.32 (m, 12H), 4.85 (t, J = 3.7 Hz, 2H), 1.44 (dd, J = 8.2, 5.6 Hz, 4H), 1.36 (dd, J = 6.6, 3.6 Hz, 4H), 1.16 – 1.08 (m, 4H) ppm. ^{13}C NMR (100 MHz, $CDCl_3$, 25 °C): δ 135.1, 134.7, 129.4, 127.9, 32.6, 24.2, 12.1 ppm. ^{29}Si NMR (79.3 MHz, $CDCl_3$, 25 °C): δ -14.7 ppm. ESI-MS: m/z calc. for $C_{30}H_{35}Si_2$ $[M+H]^+$ 451.2277, found 451.2283.

(2-Butoxyethyl)diphenylsilane[2]

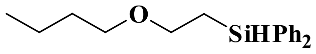

^{1}H NMR (500 MHz, $CDCl_3$, 25 °C): δ 7.63 (dd, J = 12.5, 11.3 Hz, 4H), 7.46 – 7.35 (m, 6H), 4.98 (t, J = 3.5 Hz, 1H), 3.66 (t, J = 8.8 Hz, 2H), 3.41 (t, J = 6.6 Hz, 2H), 1.64 (ddd, J = 18.7, 11.2, 7.2 Hz, 2H), 1.61 – 1.52 (m, 2H), 1.40 (dq, J = 14.6, 7.4 Hz, 2H), 0.96 (t, J = 7.4 Hz, 3H) ppm. ^{13}C NMR (100 MHz, $CDCl_3$, 25 °C): δ 135.1, 134.3, 129.6, 127.9, 70.2, 67.3, 31.8, 19.3, 14.2, 13.9 ppm. ^{29}Si NMR (79.3 MHz, $CDCl_3$, 25 °C): δ -16.8 ppm.

(3-Phenylpropyl)diphenylsiIane[3]

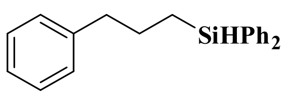

^{1}H NMR (500 MHz, $CDCl_3$, 25 °C): δ 7.60 – 7.57 (m, 4H), 7.44 – 7.38 (m, 6H), 7.33 – 7.28 (m, 2H), 7.24 – 7.16 (m, 3H), 4.93 (t, J = 3.7 Hz, 1H), 2.72 (t, J = 7.6 Hz, 2H), 1.88 – 1.81 (m, 2H), 1.26 – 1.21 (m, 2H) ppm. ^{13}C NMR (100 MHz, $CDCl_3$, 25 °C): δ 142.1, 135.1, 134.3, 129.5, 128.5, 128.2, 128.0, 125.7, 39.2, 26.3, 11.8 ppm. ^{29}Si NMR (79.3 MHz, $CDCl_3$, 25 °C): δ -13.9 ppm.

(3-(4-Methoxyphenyl)propyl)diphenylsilane

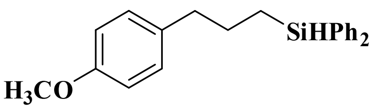

^{1}H NMR (500 MHz, $CDCl_3$, 25 °C): δ 7.58 – 7.53 (m, 4H), 7.42 – 7.34 (m, 6H), 7.08 – 7.04 (m, 2H), 6.84 – 6.81 (m, 2H), 4.89 (t, J = 3.7 Hz, 1H), 3.80 (s, 3H), 2.63 (t, J = 7.5 Hz, 2H), 1.81 – 1.72 (m, 2H), 1.22 – 1.14 (m, 2H) ppm. ^{13}C NMR (100 MHz, $CDCl_3$, 25 °C): δ 157.7, 135.1, 134.4, 134.3, 129.5, 129.4, 127.9, 113.7, 55.2, 38.3, 26.5, 11.7 ppm. ^{29}Si NMR (79.3 MHz, $CDCl_3$, 25 °C): δ -13.9 ppm. ESI-MS: m/z calc. for $C_{22}H_{25}OSi$ $[M+H]^+$ 333.1674, found 333.1679.

(2-(Cyclohexyloxy)ethyl)diphenylsilane

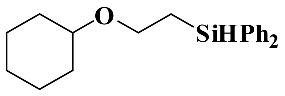

^{1}H NMR (500 MHz, $CDCl_3$, 25 °C): δ 7.60 – 7.55 (m, 4H), 7.41 – 7.34 (m, 6H), 4.91 (t, *J* = 3.6 Hz, 1H), 3.62 (t, *J* = 8.8 Hz, 2H), 3.17 (td, *J* = 9.1, 3.9 Hz, 1H), 1.89 – 1.81 (m, 2H), 1.60 – 1.54 (m, 2H), 1.54 – 1.49 (m, 2H), 1.21 (ddd, *J* = 16.9, 9.9, 7.5 Hz, 6H) ppm. ^{13}C NMR (100 MHz, $CDCl_3$, 25 °C): δ 135.1, 134.3, 129.6, 127.9, 77.2, 64.2, 32.3, 25.8, 24.1, 14.6 ppm. ^{29}Si NMR (79.3 MHz, $CDCl_3$, 25 °C): δ -16.7 ppm. ESI-MS: m/z calc. for $C_{20}H_{27}OSi$ $[M+H]^+$ 311.1831, found 311.1835.

(3-Phenoxypropyl)diphenylsilane

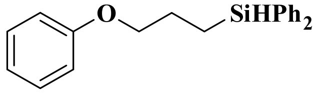

^{1}H NMR (500 MHz, $CDCl_3$, 25 °C): δ 7.64 – 7.59 (m, 4H), 7.45 – 7.37 (m, 6H), 7.32 – 7.26 (m, 2H), 6.96 (t, *J* = 7.3 Hz, 1H), 6.90 (d, *J* = 7.8 Hz, 2H), 4.96 (t, *J* = 3.7 Hz, 1H), 3.98 (t, *J* = 6.5 Hz, 2H), 2.02 – 1.92 (m, 2H), 1.37 – 1.29 (m, 2H) ppm. ^{13}C NMR (100 MHz, $CDCl_3$, 25 °C): δ 158.9, 135.1, 134.6, 129.6, 129.4, 128.0, 120.5, 114.5, 69.8, 24.3, 8.4 ppm. ^{29}Si NMR (79.3 MHz, $CDCl_3$, 25 °C): δ -13.7 ppm. ESI-MS: m/z calc. for $C_{21}H_{23}OSi$ $[M+H]^+$ 319.1518, found 319.1514.

5-(Diphenylsilyl)pentyl benzoate

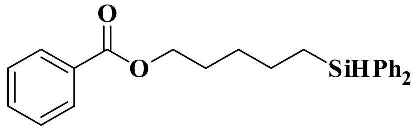

^{1}H NMR (500 MHz, $CDCl_3$, 25 °C): δ 8.05 (d, *J* = 7.4 Hz, 2H), 7.61 – 7.54 (m, 5H), 7.46 – 7.43 (m, 3H), 7.41 – 7.34 (m, 5H), 4.89 (t, *J* = 3.7 Hz, 1H), 4.30 (t, *J* = 6.6 Hz, 2H), 1.78 (dt, *J* = 13.2, 6.7 Hz, 2H), 1.55 (dd, *J* = 13.2, 9.5 Hz, 4H), 1.23 – 1.16 (m, 2H) ppm. ^{13}C NMR (100 MHz, $CDCl_3$, 25 °C): δ 166.6, 135.1, 134.4, 132.7, 130.5, 129.5, 129.5, 128.3, 128.0, 65.0, 29.5, 28.3, 24.1, 12.1 ppm. ^{29}Si NMR (79.3 MHz, $CDCl_3$, 25 °C): δ -13.9 ppm. ESI-MS: m/z calc. for $C_{24}H_{27}O_2Si$ $[M+H]^+$ 375.1780; found 375.1782.

9-(5-(Diphenylsilyl)pentyl)-9H-carbazole

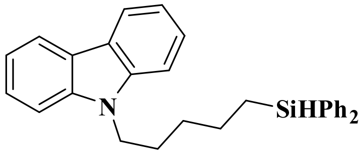

^{1}H NMR (500 MHz, $CDCl_3$, 25 °C): δ 8.14 – 8.13 (m, 2H), 7.58 – 7.55 (m, 4H), 7.50 (dd, *J* = 7.1, 4.5 Hz, 2H), 7.43 – 7.35 (m, 6H), 7.27 – 7.24 (m, 2H), 7.21 (t, *J* = 7.5 Hz, 2H), 4.86 (t, *J* = 3.5 Hz, 1H), 4.28 (t, *J* = 7.2 Hz, 2H), 1.92 – 1.85 (m, 2H), 1.53 – 1.45 (m, 4H), 1.18 – 1.11 (m, 2H) ppm. ^{13}C NMR (100 MHz, $CDCl_3$, 25 °C): δ 140.4, 135.1, 134.3, 130.0, 129.5, 128.0, 127.6, 125.5, 122.8, 120.3, 118.7, 108.6, 42.9, 30.6, 28.5, 24.2, 12.1 ppm. ^{29}Si NMR (79.3 MHz, $CDCl_3$, 25 °C): δ -13.9 ppm. ESI-MS: m/z calc. for $C_{29}H_{30}NSi$ $[M+H]^+$ 420.2147, found 420.2149

1-(5-(Diphenylsilyl)pentyl)-1H-indole

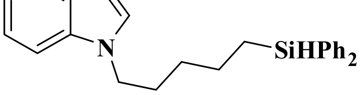

^{1}H NMR (500 MHz, $CDCl_3$, 25 °C): δ 7.66 (ddd, J = 9.3, 3.7, 2.7 Hz, 1H), 7.58 – 7.55 (m, 4H), 7.45 – 7.36 (m, 6H), 7.34 – 7.31 (m, 1H), 7.22 (tt, J = 13.8, 3.9 Hz, 1H), 7.15 – 7.10 (m, 1H), 7.06 (d, J = 3.1 Hz, 1H), 6.50 (dd, J = 3.1, 0.8 Hz, 1H), 4.87 (t, J = 3.7 Hz, 1H), 4.08 (t, J = 7.1 Hz, 2H), 1.83 (dq, J = 14.9, 7.5 Hz, 2H), 1.54 – 1.48 (m, 2H), 1.41 (ddd, J = 19.2, 9.1, 6.4 Hz, 2H), 1.15 (ddd, J = 13.6, 7.3, 4.6 Hz, 2H) ppm. ^{13}C NMR (100 MHz, $CDCl_3$, 25 °C): δ 135.9, 135.1, 134.3, 129.5, 128.0, 127.7, 127.6, 121.3, 120.9, 109.3, 100.8, 46.2, 30.4, 29.8, 24.1, 12.1 ppm. ^{29}Si NMR (79.3 MHz, $CDCl_3$, 25 °C): δ -13.9 ppm. ESI-MS: m/z calc. for $C_{25}H_{28}NSi$ $[M+H]^+$ 370.1991, found 370.1996.

6-((6-(Diphenylsilyl)pentyl)oxy)-4-methyl-2H-chromen-2-one

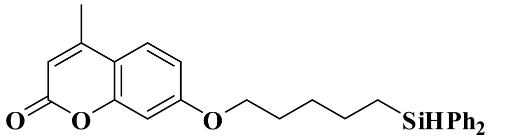

^{1}H NMR (500 MHz, $CDCl_3$, 25 °C): δ 7.58 – 7.53 (m, 4H), 7.47 (d, J = 8.8 Hz, 1H), 7.40 – 7.34 (m, 6H), 6.81 (dt, J = 9.5, 2.8 Hz, 1H), 6.77 (dd, J = 4.4, 2.0 Hz, 1H), 6.12 (t, J = 2.4 Hz, 1H), 4.87 (t, J = 3.7 Hz, 1H), 3.96 (t, J = 6.5 Hz, 2H), 2.39 (s, 3H), 1.83 – 1.76 (m, 2H), 1.57 – 1.51 (m, 4H), 1.22 – 1.14 (m, 2H) ppm. ^{13}C NMR (100 MHz, $CDCl_3$, 25 °C): δ 162.2, 161.4, 155.3, 152.5, 135.1, 134.4, 129.5, 128.0, 125.4, 113.4, 112.6, 111.8, 101.3, 68.4, 29.3, 28.5, 24.1 18.6, 12.1 ppm. ^{29}Si NMR (79.3 MHz, $CDCl_3$, 25 °C): δ -13.9 ppm. ESI-MS: m/z calc. for $C_{27}H_{29}O_3Si$ $[M+H]^+$ 429.1886, found 428.1882.

2. Spectroscopic data of products in catalytic double hydrosilylation with $PhSiH_3$.

Dioctylphenylsilane [1]

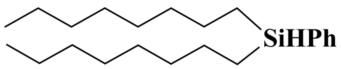

^{1}H NMR (500 MHz, $CDCl_3$, 25 °C): δ 7.55-7.54 (m, 2H), 7.37-7.35 (m, 3H), 4.27 (t, J = 4.0 Hz, 1H), 1.40-1.33 (m, 6H), 1.31-1.25 (m, 18H), 0.91-0.83 (m, 10H) ppm. ^{13}C NMR (100 MHz, $CDCl_3$, 25 °C): δ 136.2, 134.6, 129.0, 127.7, 33.2, 31.9, 29.2, 24.5, 22.6, 14.1, 11.9 ppm. ^{29}Si NMR (79.3 MHz, $CDCl_3$, 25 °C): δ -9.4 ppm.

Bis(5-bromopentyl)(phenyl)silane

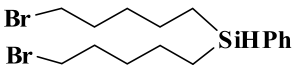

^{1}H NMR (500 MHz, $CDCl_3$, 25 °C): δ 7.52 – 7.49 (m, 2H), 7.39 – 7.33 (m, 3H), 4.26 (t, J = 3.5 Hz, 1H), 3.37 (t, J = 6.9 Hz, 4H), 1.89 – 1.77 (m, 4H), 1.51 – 1.43 (m, 4H), 1.43 – 1.34 (m, 4H), 0.89 – 0.81 (m, 4H) ppm. ^{13}C NMR (100 MHz, $CDCl_3$, 25 °C): δ δ 134.6, 133.4, 129.3, 127.9, 33.8, 32.4, 31.6, 23.7, 11.8 ppm. ^{29}Si NMR (79.3 MHz, $CDCl_3$, 25 °C): δ -9.5 ppm. ESI-MS: m/z calc. for $C_{16}H_{27}Br_2Si$ $[M+H]^+$ 405.0248, found 406.0252.

Bis(5-bromopentyl)(butyl)silane

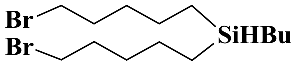

^{1}H NMR (400 MHz, $CDCl_3$, 25 °C): δ 3.68 (dt, J = 6.6, 3.1 Hz, 1H), 3.40 (t, J = 6.9 Hz, 4H), 1.93 – 1.80 (m, 4H), 1.51 – 1.42 (m, 4H), 1.42 – 1.28 (m, 8H), 0.89 (t, J = 6.8 Hz, 3H), 0.66 – 0.55 (m, 6H) ppm. ^{13}C NMR (100 MHz, $CDCl_3$, 25 °C): δ 33.9, 32.5, 31.7, 26.8, 26.3, 23.9, 13.7, 11.1, 10.9 ppm. ^{29}Si NMR (79.3 MHz, $CDCl_3$, 25 °C): δ -6.0 ppm. ESI-MS: m/z calc. for $C_{14}H_{31}Br_2Si$ $[M+H]^+$ 385.0561, found 385.0558.

Bis(4-bromobutyl)(butyl)silane

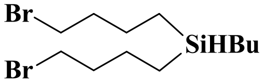

^{1}H NMR (400 MHz, $CDCl_3$, 25 °C): δ 3.75 – 3.67 (m, 1H), 3.41 (t, J = 6.8 Hz, 4H), 1.95 – 1.82 (m, 4H), 1.53-1.48 (m, 4H), 1.35 – 1.26 (m, 4H), 0.88 (dd, J = 8.7, 4.8 Hz, 3H), 0.65 – 0.54 (m, 6H) ppm. ^{13}C NMR (100 MHz, $CDCl_3$, 25 °C): δ 35.9, 33.5, 26.7, 26.2, 23.1, 13.7, 10.7, 10.2 ppm. ^{29}Si NMR (79.3 MHz, $CDCl_3$, 25 °C): δ -6.1 ppm. ESI-MS: m/z calc. for $C_{12}H_{27}Br_2Si$ $[M+H]^+$ 357.0248, found 357.0257.

(5-Bromopentyl)(octyl)(phenyl)silane

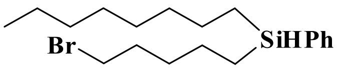

^{1}H NMR (500 MHz, $CDCl_3$, 25 °C): δ 7.54 – 7.50 (m, 2H), 7.39 – 7.32 (m, 3H), 4.26 (dd, J = 7.0, 3.5 Hz, 1H), 3.39 – 3.37 (m, 2H), 1.89 – 1.80 (m, 2H), 1.41 – 1.22 (m, 11H), 0.91 – 0.81 (m, 12H) ppm. ^{13}C NMR (100 MHz, $CDCl_3$, 25 °C): δ 135.8, 134.6, 129.2, 127.8, 33.8, 33.2, 32.5, 31.9, 31.6, 29.2, 24.5, 24.5, 23.8, 22.6, 14.1, 11.9, 11.8 ppm. ^{29}Si NMR (79.3 MHz, $CDCl_3$, 25 °C): δ -9.4 ppm. ESI-MS: m/z calc. for $C_{19}H_{34}BrSi$ $[M+H]^+$ 369.1613, found 369.1618.

(4-bromobutyl)(octyl)(phenyl)silane

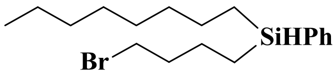

^{1}H NMR (400 MHz, $CDCl_3$, 25 °C): δ 7.53 – 7.50 (m, 2H), 7.38 – 7.32 (m, 3H), 4.26 (p, J = 3.3 Hz, 1H), 3.38 (t, J = 6.9 Hz, 2H), 1.92 – 1.84 (m, 2H), 1.57 – 1.48 (m, 2H), 1.40 – 1.20 (m, 11H), 0.91 – 0.80 (m, 8H) ppm. ^{13}C NMR (100 MHz, $CDCl_3$, 25 °C): δ 135.2, 134.7, 129.3, 127.9, 36.0, 33.4, 33.3, 31.9, 29.3, 29.3, 24.5, 23.2, 22.7, 14.2, 11.8, 11.1 ppm. ^{29}Si NMR (79.3 MHz, $CDCl_3$, 25 °C): δ -9.0 ppm. ESI-MS: m/z calc. for $C_{18}H_{32}BrSi$ $[M+H]^+$ 355.1456, found 355.1465.

(5-bromopentyl)(butyl)(octyl)silane

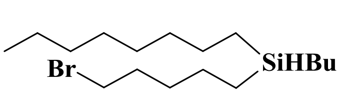

^{1}H NMR (400 MHz, $CDCl_3$, 25 °C): δ 3.71 – 3.63 (m, 1H), 3.41 (t, J = 6.9 Hz, 2H), 1.91 – 1.82 (m, 2H), 1.51 – 1.42 (m, 2H), 1.41 – 1.22 (m, 18H), 0.92 – 0.85 (m, 6H), 0.63 – 0.54 (m, 6H) ppm. ^{13}C NMR (100 MHz, $CDCl_3$, 25 °C): δ 33.9, 33.3, 32.5, 31.9, 31.7, 29.3, 29.2, 26.9, 26.8, 26.3, 24.6, 23.9, 22.6, 14.1, 13.7, 11.2, 10.9 ppm. ^{29}Si NMR (79.3 MHz, $CDCl_3$, 25 °C): δ -5.9 ppm. ESI-MS: m/z calc. for $C_{17}H_{38}BrSi$ $[M+H]^+$ 349.1926, found 349.1935.

(4-bromobutyl)(butyl)(octyl)silane

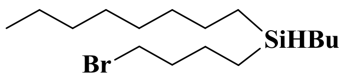

^{1}H NMR (400 MHz, $CDCl_3$, 25 °C): δ 3.71-3.65 (m, 1H), 3.42 (t, J = 6.8 Hz, 2H), 1.94 – 1.83 (m, 2H), 1.53 – 1.44 (m, 2H), 1.38 – 1.20 (m, 15H), 0.94 – 0.84 (m, 7H), 0.68 – 0.53 (m, 6H) ppm. ^{13}C NMR (100 MHz, $CDCl_3$, 25 °C): δ 36.0, 33.5, 33.3, 31.9, 29.3, 29.2, 26.8, 26.3, 24.7, 24.6, 23.3, 22.6, 14.1, 13.7, 10.9, 10.4 ppm. ^{29}Si NMR (79.3 MHz, $CDCl_3$, 25 °C): δ -6.0 ppm. ESI-MS: m/z calc. for $C_{16}H_{36}BrSi$ $[M+H]^+$ 335.1769, found 335.1776.

(4-Bromobutyl)(5-bromopentyl)(phenyl)silane

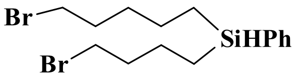

^{1}H NMR (500 MHz, $CDCl_3$, 25 °C): δ 7.54 – 7.50 (m, 2H), 7.40 – 7.34 (m, 3H), 4.28 (dt, J = 7.2, 3.7 Hz, 1H), 3.42 – 3.35 (m, 4H), 1.93 – 1.80 (m, 4H), 1.57 – 1.36 (m, 6H), 0.91 – 0.82 (m, 4H) ppm. ^{13}C NMR (100 MHz, $CDCl_3$, 25 °C): δ 135.1, 134.6, 129.4, 127.9, 35.8, 34.0, 33.3, 32.4, 31.6, 23.7, 23.0, 11.7, 11.0 ppm. ^{29}Si NMR (79.3 MHz, $CDCl_3$, 25 °C): δ -9.6 ppm. ESI-MS: m/z calc. for $C_{15}H_{25}Br_2Si$ $[M+H]^+$ 391.0092, found 391.0095.

Octyl(3-phenoxypropyl)(phenyl)silane

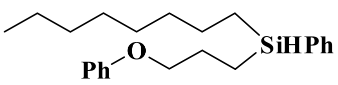

^{1}H NMR (500 MHz, $CDCl_3$, 25 °C): δ 7.56 – 7.52 (m, 2H), 7.40 – 7.33 (m, 3H), 7.28 – 7.23 (m, 3H), 6.88 – 6.83 (m, 2H), 4.35 – 4.28 (m, 1H), 3.92 (t, J = 6.7 Hz, 2H), 1.91 – 1.82 (m, 2H), 1.49 – 1.36 (m, 2H), 1.31 – 1.20 (m, 8H), 1.02 – 0.94 (m, 4H), 0.93 – 0.82 (m, 5H) ppm. ^{13}C NMR (100 MHz, $CDCl_3$, 25 °C): δ 134.6, 134.3, 129.4, 129.3, 127.9, 120.5, 114.5, 70.0, 33.2, 31.9, 29.2, 24.4, 22.6, 17.9, 14.4, 14.1, 11.8, 8.1 ppm. ^{29}Si NMR (79.3 MHz, $CDCl_3$, 25 °C): δ -9.1 ppm. ESI-MS: m/z calc. for $C_{23}H_{35}OSi$ $[M+H]^+$ 355.2457, found 355.2454.

3. Spectroscopic data of products in catalytic hydrosilylation with HMTS.

Bis(trimethylsiloxy)methyloctylsilane.[4]

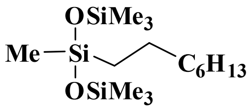

^{1}H NMR (500 MHz, $CDCl_3$, 25 °C): δ 1.33-1.26 (m, 12H), 0.88 (t, 6.8 Hz, 3H), 0.49-0.47 (m, 2H), 0.09 (s, 18H), -0.01 (s, 3H) ppm. ^{13}C NMR (100 MHz, $CDCl_3$, 25 °C): δ 33.7, 32.0, 29.4, 29.3, 23.9, 22.7, 16.7, 14.1, 1.7, -1.6 ppm. ^{29}Si NMR (79.3 MHz, $CDCl_3$, 25 °C): δ 6.6, -10.7 ppm.

Bis(trimethylsiloxy)methylhexylsilane.

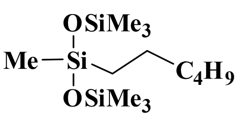

^{1}H NMR (500 MHz, $CDCl_3$, 25 °C): δ 1.33-1.26 (m, 8H), 0.88 (t, 6.8 Hz, 3H), 0.59-0.57 (m, 2H), 0.10 (s, 18H), 0.02 (s, 3H) ppm. ^{13}C NMR (100 MHz, $CDCl_3$, 25 °C): δ 32.9, 31.6, 24.5, 22.6, 14.1, 12.8, 1.7, -2.2 ppm. ^{29}Si NMR (79.3 MHz, $CDCl_3$, 25 °C): δ 6.5, -10.7 ppm. ESI-MS: m/z calc. for $C_{13}H_{35}O_2Si_3$ $[M+H]^+$ 307.1945, found 306.1949.

Hydrosilylation of 1-octene by PMHS.[5]

^{1}H NMR (500 MHz, $CDCl_3$, 25 °C): δ 1.31-1.26 (m, $(CH_2)_6$), 0.88 (t, 6.8 Hz, $-(CH_2)_6-CH_3$), 0.59-0.57 (m, $SiCH_2-$), 0.15-0.11 (m, $Si(CH_3)_3$), 0.03 (s, $SiCH_3$) ppm. ^{13}C NMR (100 MHz, $CDCl_3$, 25 °C): δ 33.3, 31.9, 29.3, 29.2, 24.5, 22.7, 14.1, 12.8 ppm.

Hydrosilylation of 1-hexene by PMHS.

^{1}H NMR (500 MHz, $CDCl_3$, 25 °C): δ 1.32-1.25 (m, $(CH_2)_6$), 0.88 (t, 6.8 Hz, $-(CH_2)_6-CH_3$), 0.57-0.55 (m, $SiCH_2-$), 0.15-0.07 (m, $Si(CH_3)_3$), 0.03 (s, $SiCH_3$) ppm. ^{13}C NMR (100 MHz, $CDCl_3$, 25 °C): δ 32.9, 31.6, 24.5, 22.6, 14.1, 12.7 ppm.

4. Procedure for reaction inhibition in presence of radical scavenger TEMPO.

1 (0.0012 mmol) and K (0.0037 mmol) in THF (1 mL) were taken and silane (0.5 mmol), 1-octene (0.5 mmol) and TEMPO (0.5/1 mmol) were added to it at room temperature. The reaction was stirred at room temperature for 30 min and then quenched by exposing the reaction mixture to air. The solution was concentrated under vacuum, and the product was purified by column chromatography using hexane as an eluent.

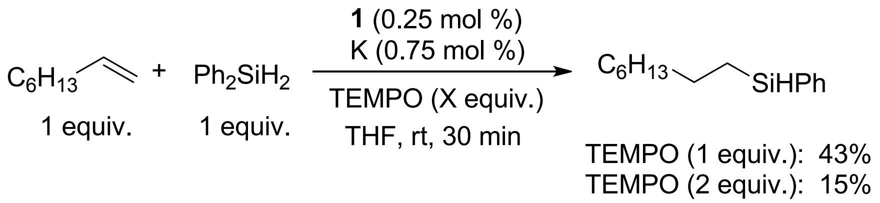

Scheme S1 Reaction inhibition in presence of TEMPO.

5. Procedure for reaction inhibition in presence of radical scavenger Galvinoxyl radical.

1 (0.0012 mmol) and K (0.0037 mmol) in THF (1 mL) were taken and silane (0.5 mmol), 1-octene (0.5 mmol) and Galvinoxyl radical (0.5/1 mmol) were added to it at room temperature. The reaction was stirred at room temperature for 30 min and then quenched by exposing the reaction mixture to air. The solution was concentrated under vacuum, and the product was purified by column chromatography using hexane as an eluent.

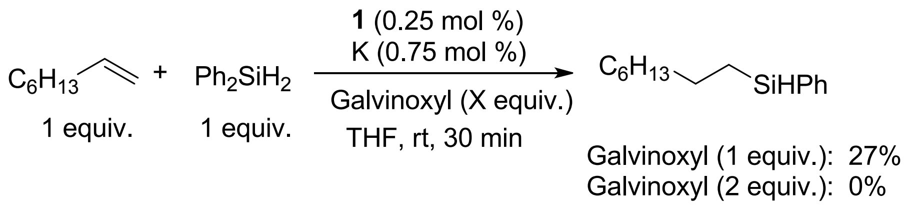

Scheme S2 Reaction inhibition in presence of galvinoxyl radical.

6. Cyclic Voltammogram of complex 1

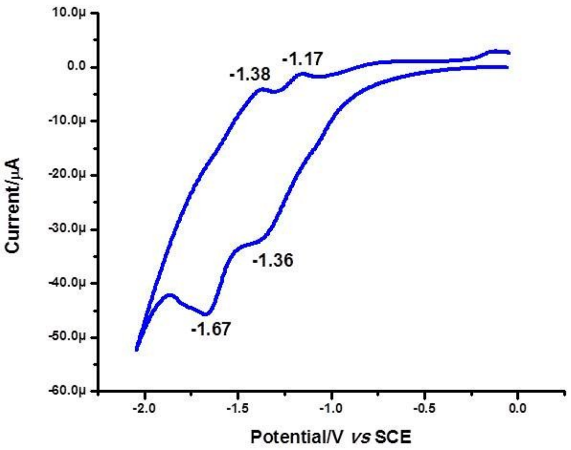

Fig. S1 Cyclic voltammogram of compound **1** in dry THF solvent.

7. Magneto-structural Correlation

To understand clearly the electronic spin structure of this linear three spin-centered system **2**, [S_i = ½; (Ply·) ↔ S_j = 2/2; (Ni^{2+}) ↔ S_k = ½; (Ply·)] we have performed the variable temperature dc magnetic susceptibility measurement for both the complexes in an applied field of 0.1 Tesla, temperature ranging from 2 to 300K. The thermal variation of the effective magnetic moment (μ_{eff}). The molar susceptibility of compound **1** corresponds to an effective magnetic moment of 2.82 μ_B at 300K that remains constant down to 45 K revealing a usual Curie paramagnetism for a high spin Ni^{2+} ion (S = 1). The sharp fall of the μ_{eff} values at low temperature is concomitant with the zero-field splitting. The same for compound **2** shows a magnetic moment of 3.42 μ_B at 300 K which is slightly smaller than the value anticipated for four uncoupled spins of Ni^{2+} ion and ligand radicals respectively (~ 3.7 μ_B obtained from eqn. 1). This decrease in the μ_{eff} with lowering temperature clearly indicates the persistence of antiferromagnetic interaction, even at 300 K. This data is strongly suggestive of an AF-coupled triplet as the ground state for **2** along with significant population in the quintet state over a wide temperature range.

Putting the spin angular momentum values of the uncoupled three spin-centered system -

$$\mu_{eff} = 2\sqrt{S_i(S_i+1) + S_j(S_j+1) + S_k(S_k+1)} \qquad (1)$$

This ground state for **1** and **2** from SQUID data (triplet and AF-coupled triplet respectively) is also in complete agreement with their EPR silence probed by X-band EPR.

8. X-ray photoelectron spectroscopy (XPS)

XPS Measurement Details. $Ni2p_{3/2}$ core level spectra of Ni based samples were recorded with a Thermo Fisher Scientific Multilab 2000 spectrometer using non-monochromatic AlKα radiation (1253.6 eV) run at 15 kV and 10 mA as X-ray source. The binding energies reported here were calculated with reference to C1s peak at 284.5 eV. For XPS analysis, powder solid samples from glovebox were mounted quickly on the sample holders and were kept in the preparation chamber at ultrahigh vacuum (UHV) at 5×10^{-8} mbar for 5 h in order to desorb any volatile species present on the surface. After 5 h, samples were placed into an ultrahigh vacuum (UHV) chamber at 5×10^{-9} mbar housing the analyzer. All the spectra were obtained here in the digital mode with 40 eV pass energy across the hemispheres of the electron analyzer and 0.05 eV step increment.

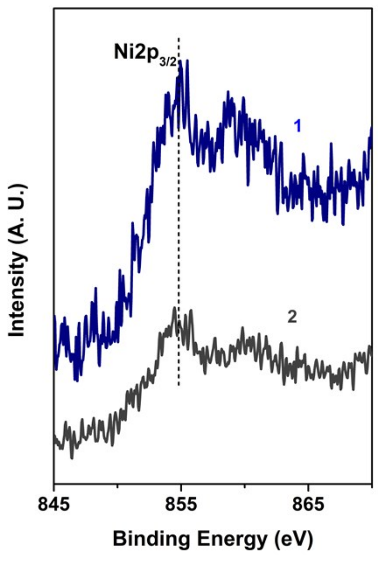

Fig. S2 X-ray photoelectron spectra for the $Ni2p_{3/2}$ core level for **1** (blue line) and **2** (gray line). The identical peak position before and after reduction proves the retention of nickel's +2 oxidation state.

9. Computational results:

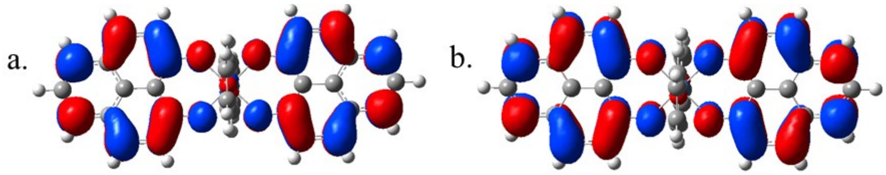

Fig. S3 a) The LUMO for **1** and b) its single electron reduced product, both cases show complete ligand based orbitals. The isosurface value is set to ± 0.02 (**e.**bohr^{-3})$^{1/2}$ for depiction

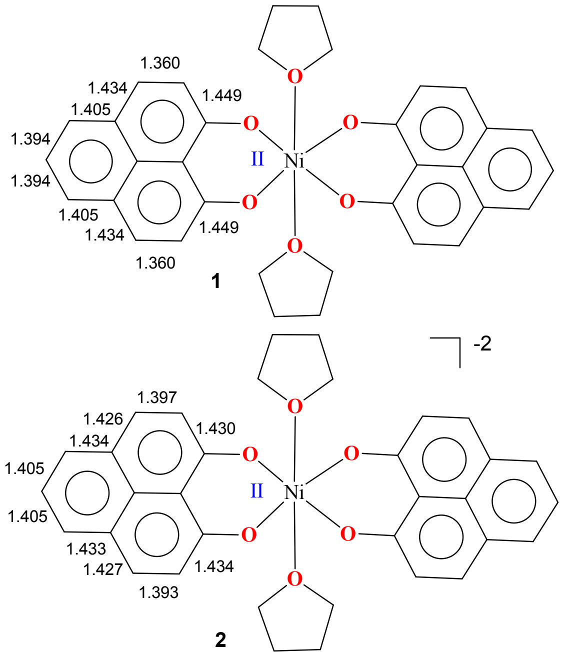

Fig. S4 Comparison of C-C bond lengths (Å) of the PLY-ligand backbone, between two-electron reduced product **2** and the pre-catalyst **1**. The bond length elongation are fully consistent with occupation of electrons in the ligand centric LUMO, thus implying the ligand can house those electrons.

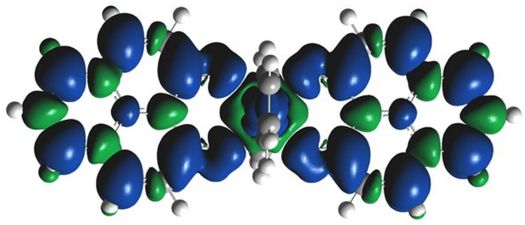

Fig. S5 Spin density plot for the one-electron reduced product from **1**. The computationally optimized molecule has a quartet ground state, and the extra electron (in addition to two unpaired electrons in high-spin Ni$^{(II)}$ complex) is fully delocalized on the PLY backbone, proving the ligand can host the electron. The contour values was set to ± 0.0004 (**e.**bohr^{-3})$^{1/2}$.

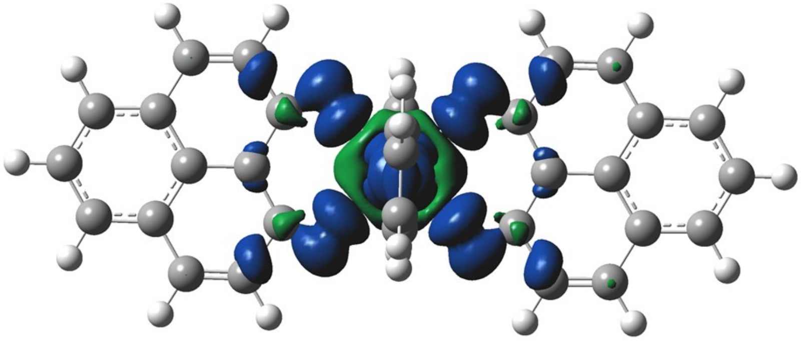

Fig. S6 Spin density plot for the paramagnetic molecule **1**. The Spin density of Ni(II) for compound **1** was computed to be 1.69, and the corresponding value for compound **2** is 1.72. The contour values was set to ± 0.0004 (**e.**bohr^{-3})$^{1/2}$.

10. ^{1}H, ^{13}C and ^{29}Si NMR spectra of hydrosilylated products.

Fig. S7 ^{1}H NMR spectrum ($CDCl_3$) of octyldiphenylsilane.

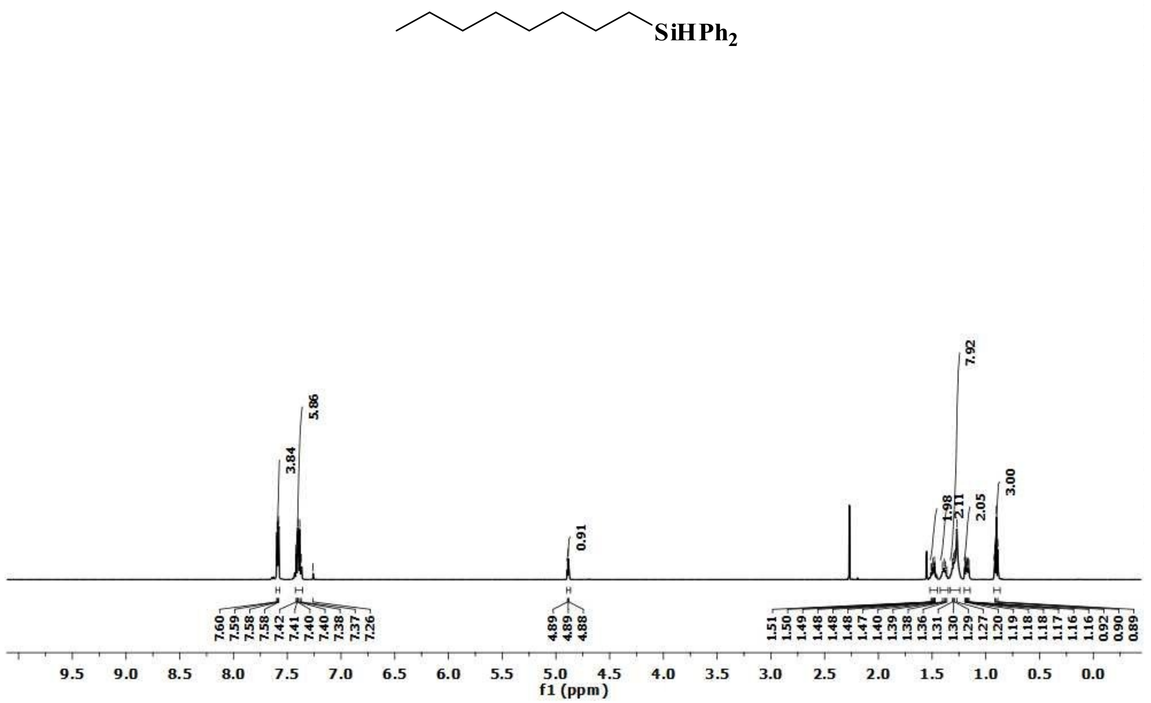

Fig. S8 ^{13}C NMR spectrum ($CDCl_3$) of octyldiphenylsilane.

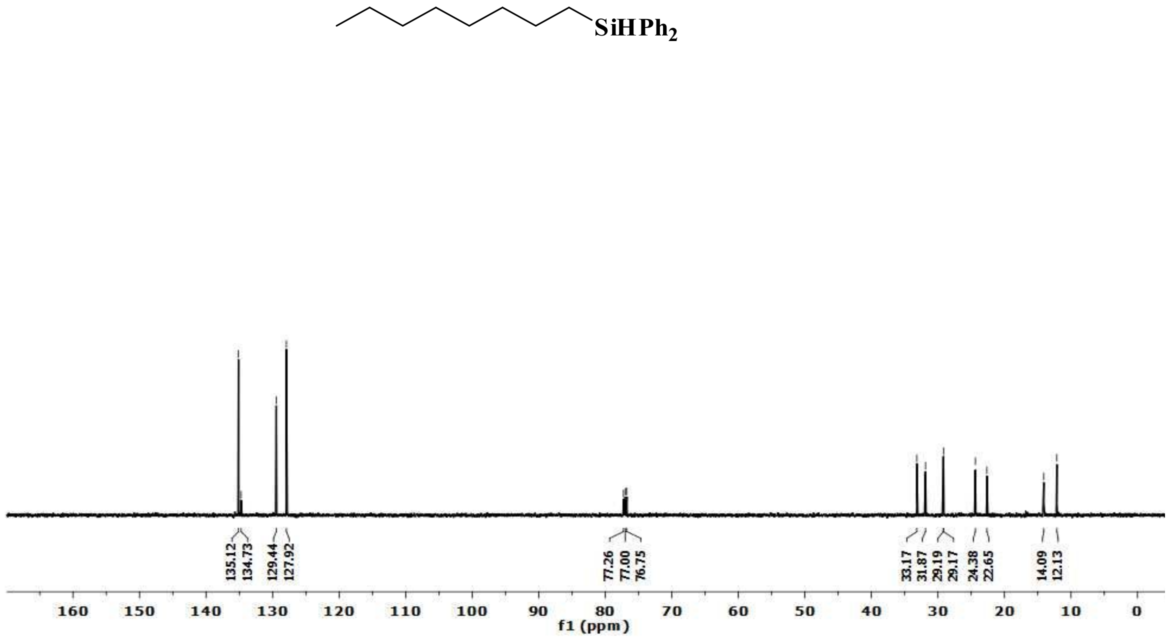

Fig. S9 ^{29}Si NMR spectrum ($CDCl_3$) of octyldiphenylsilane.

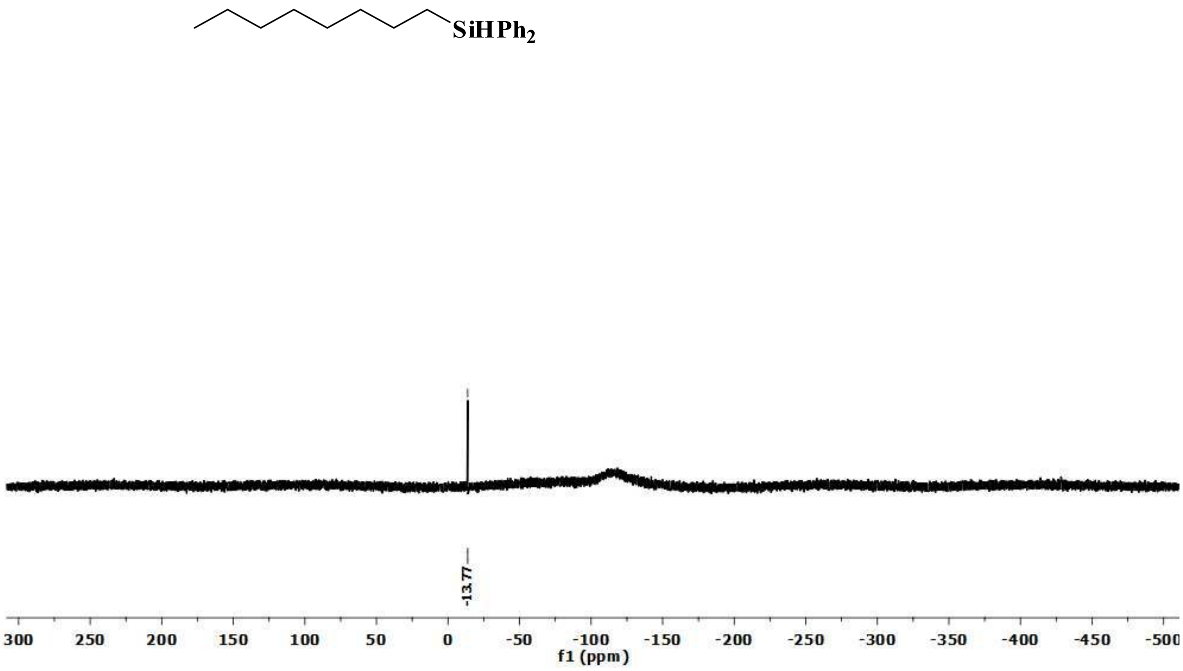

Fig. S10 ^{1}H NMR spectrum ($CDCl_3$) of hexyldiphenylsilane.

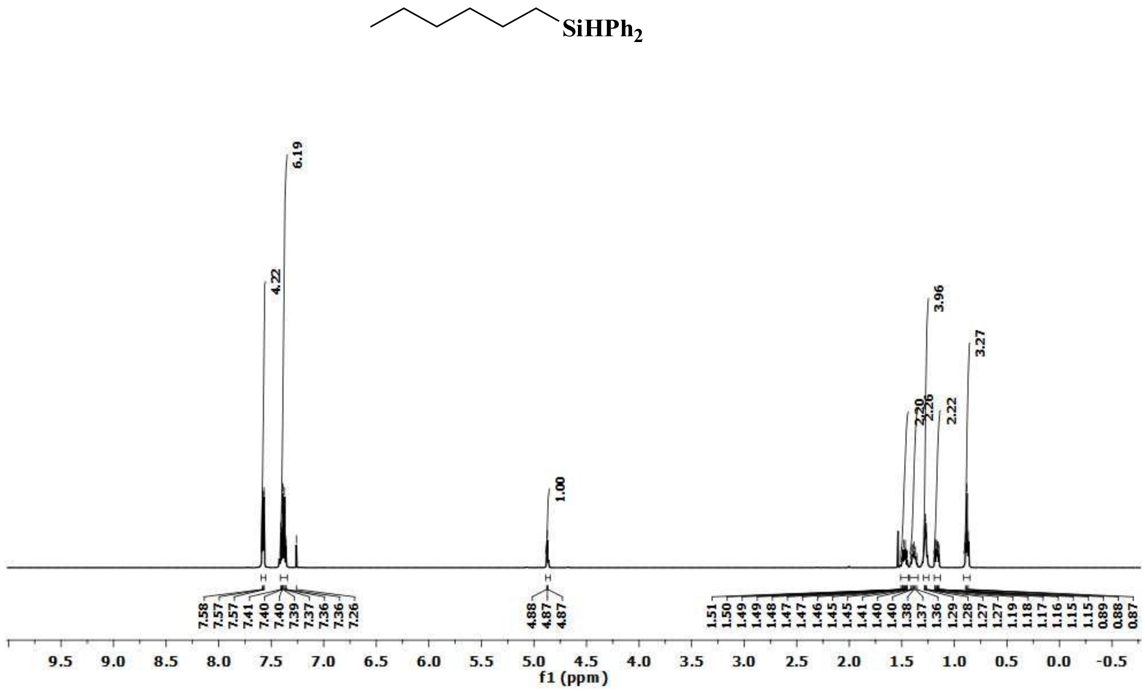

Fig. S11 ^{13}C NMR spectrum ($CDCl_3$) of hexyldiphenylsilane.

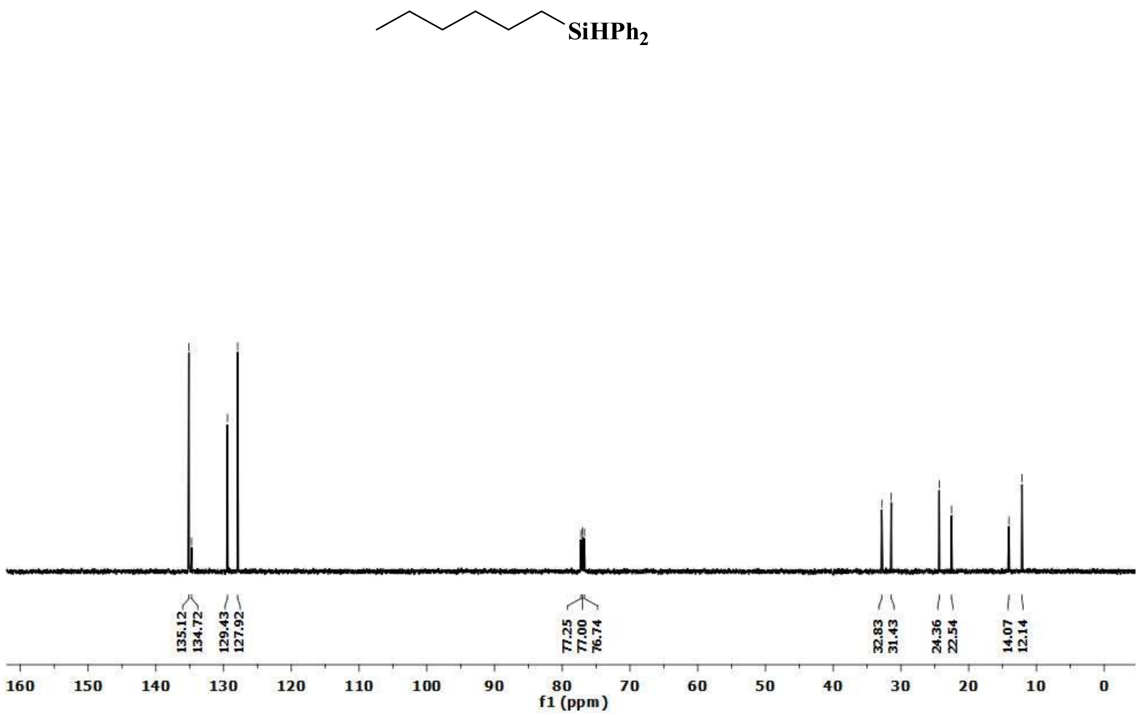

Fig. S12 ^{29}Si NMR spectrum ($CDCl_3$) of hexyldiphenylsilane.

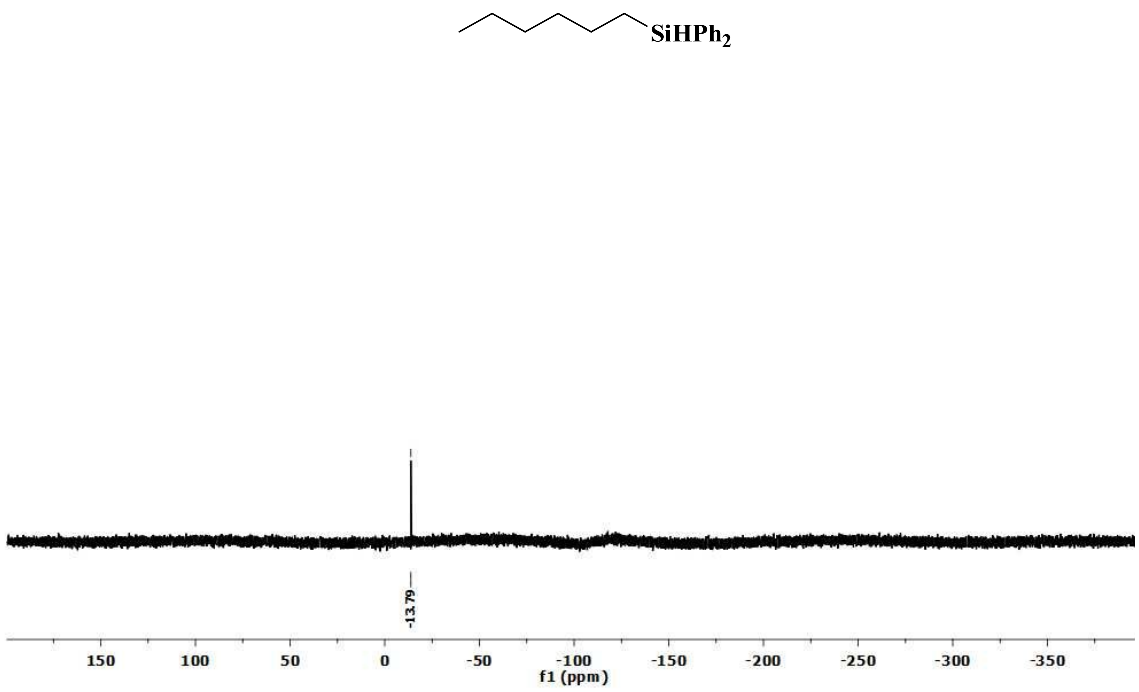

Fig. S13 ^{1}H NMR spectrum ($CDCl_3$) of (5-bromopentyl)diphenylsilane.

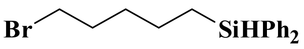

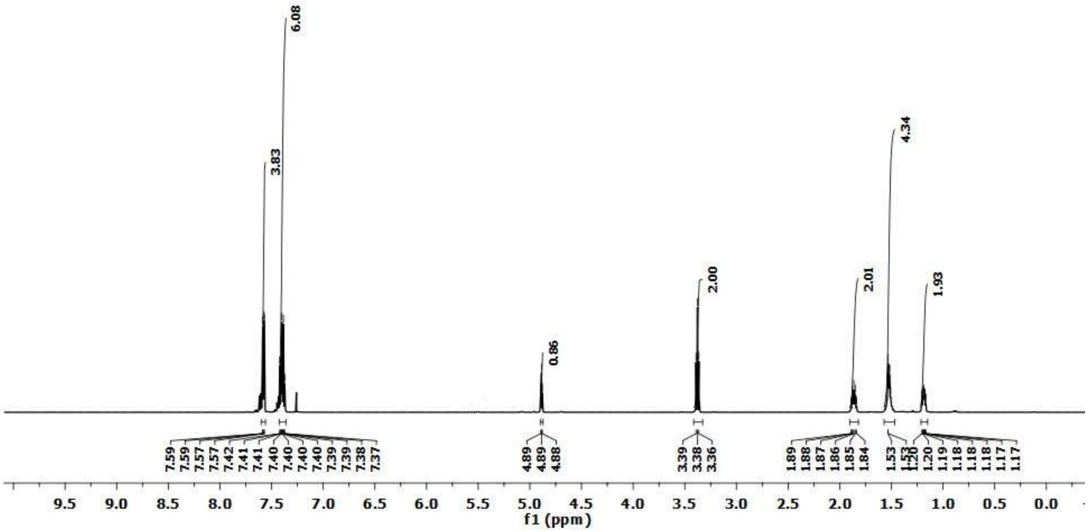

Fig. S14 ^{13}C NMR spectrum ($CDCl_3$) of (5-bromopentyl)diphenylsilane.

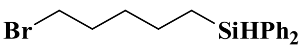

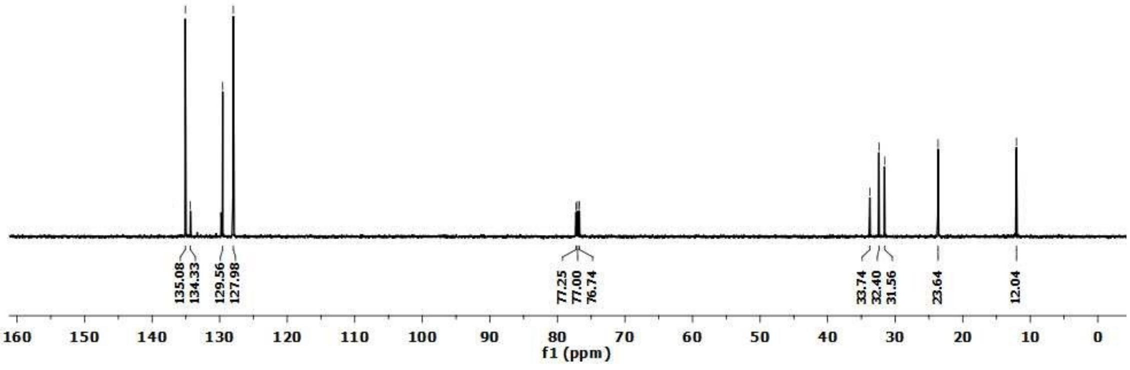

Fig. S15 ^{29}Si NMR spectrum ($CDCl_3$) of (5-bromopentyl)diphenylsilane.

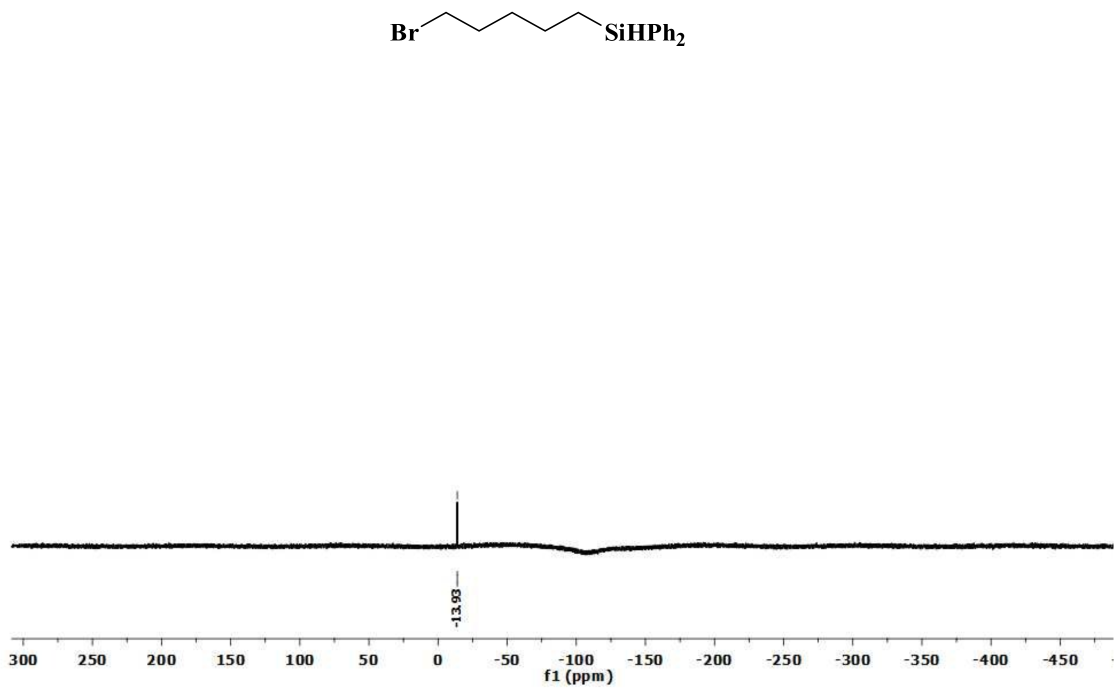

Fig. S16 ^{1}H NMR spectrum ($CDCl_3$) of (4-bromobutyl)diphenylsilane.

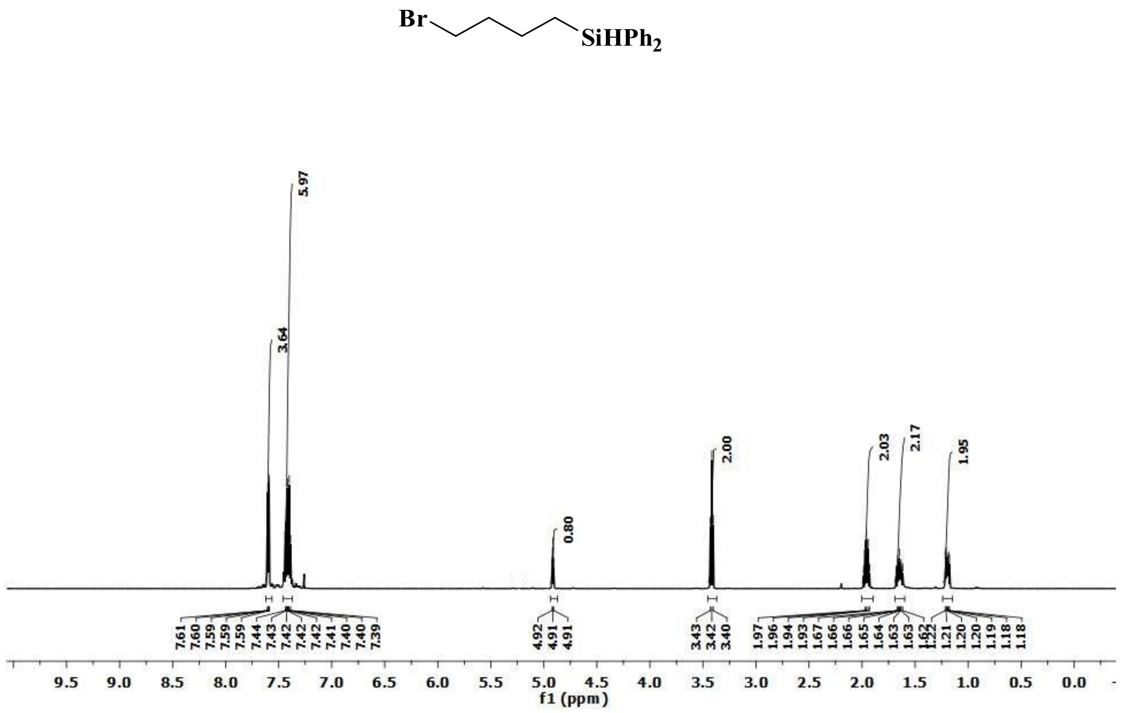

Fig. S17 ^{13}C NMR spectrum ($CDCl_3$) of (4-bromobutyl)diphenylsilane.

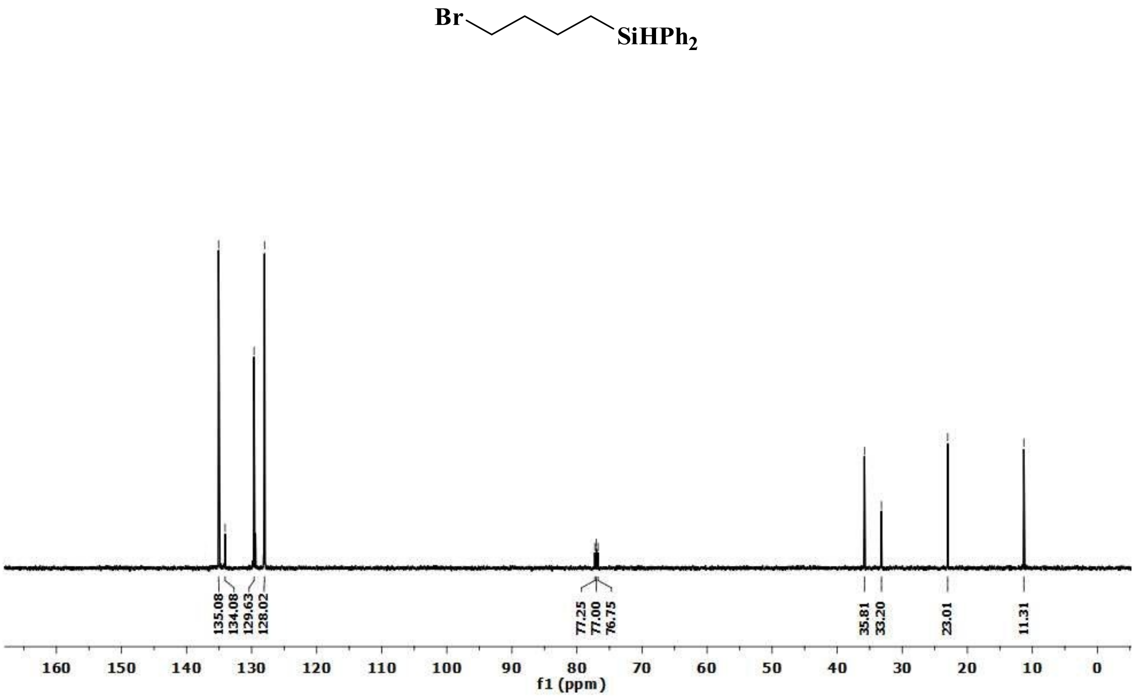

Fig. S18 ^{29}Si NMR spectrum ($CDCl_3$) of (4-bromobutyl)diphenylsilane.

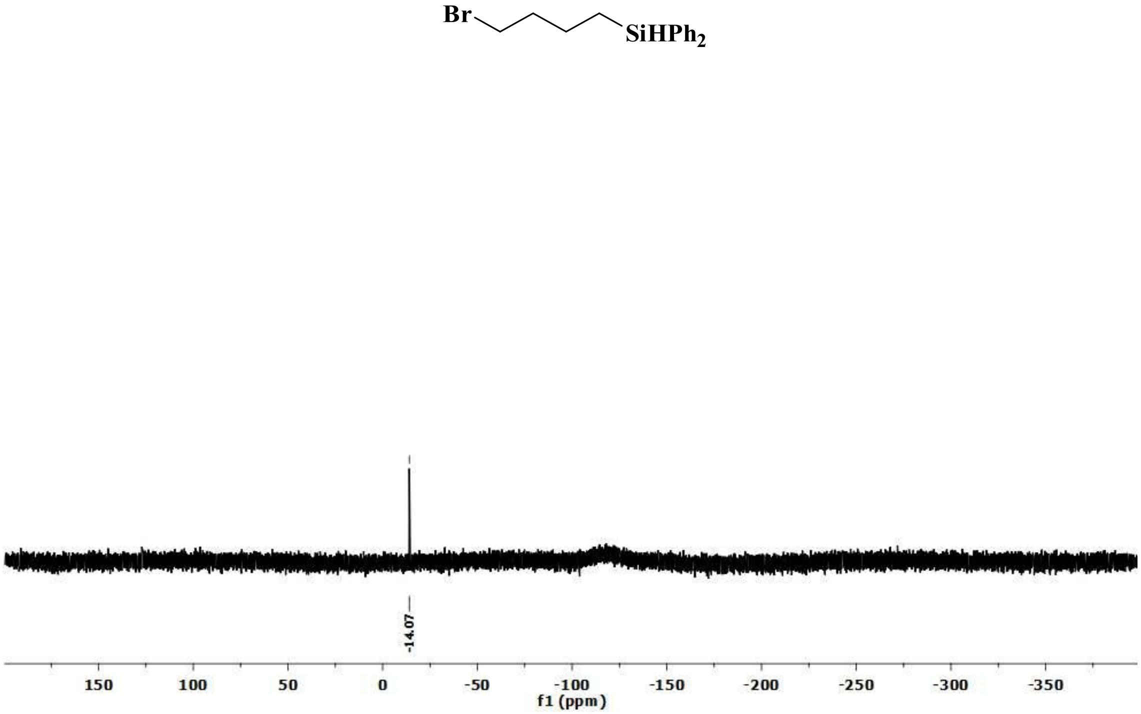

Fig. S19 ^{1}H NMR spectrum ($CDCl_3$) of 1,6-bis(diphenylsilyl)hexane.

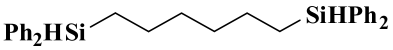

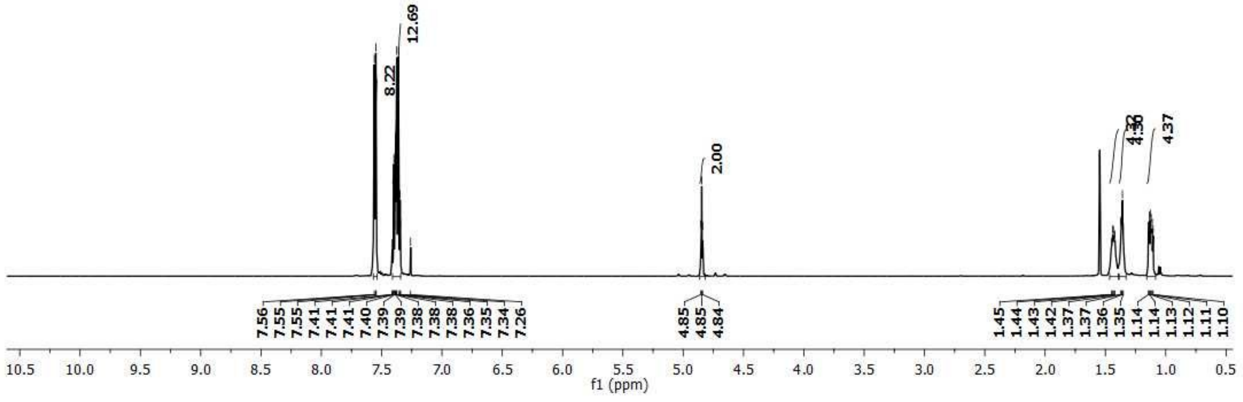

Fig. S20 ^{13}C NMR spectrum ($CDCl_3$) of 1,6-bis(diphenylsilyl)hexane.

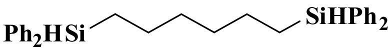

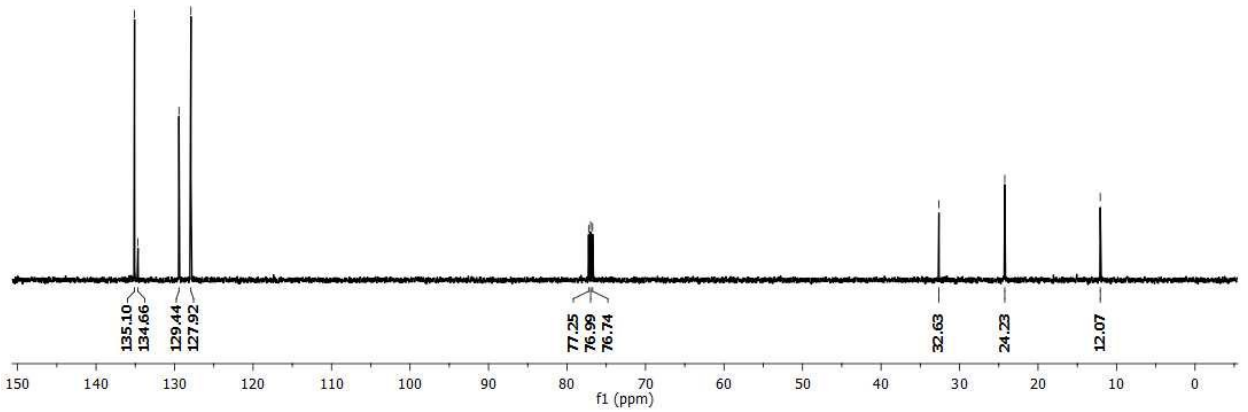

Fig. S21 ^{29}Si NMR spectrum ($CDCl_3$) of 1,6-bis(diphenylsilyl)hexane.

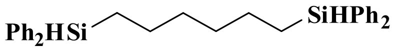

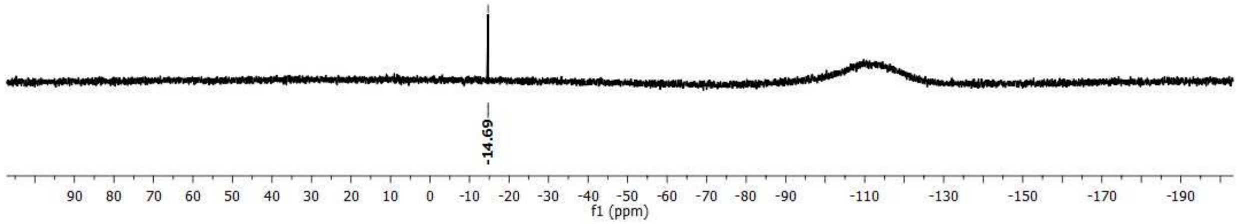

Fig. S22 ^{1}H NMR spectrum ($CDCl_3$) of (2-butoxyethyl)diphenylsilane.

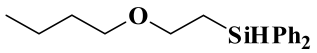

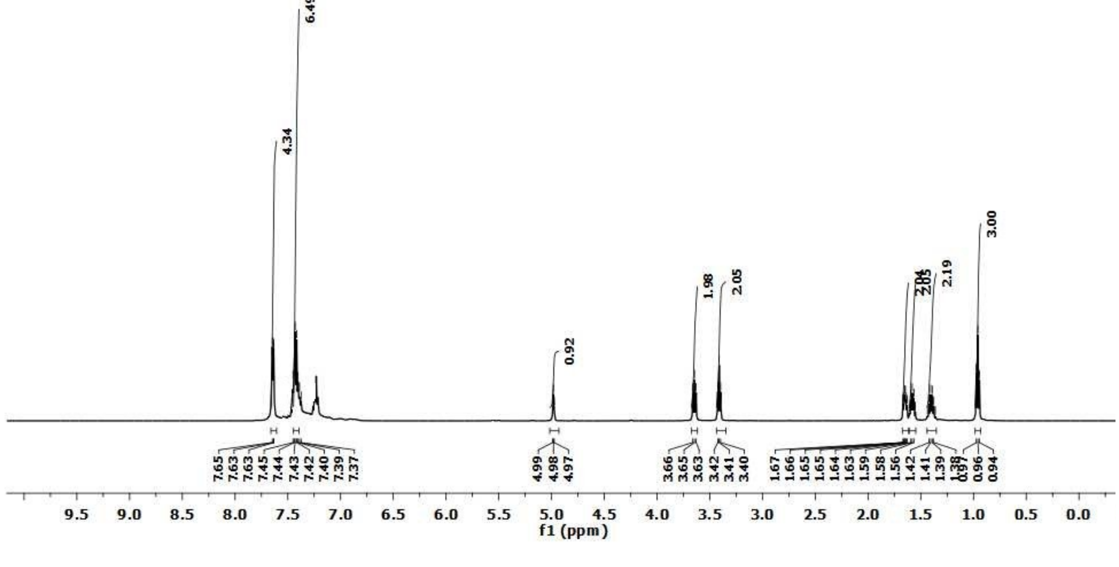

Fig. S23 ^{13}C NMR spectrum ($CDCl_3$) of (2-butoxyethyl)diphenylsilane.

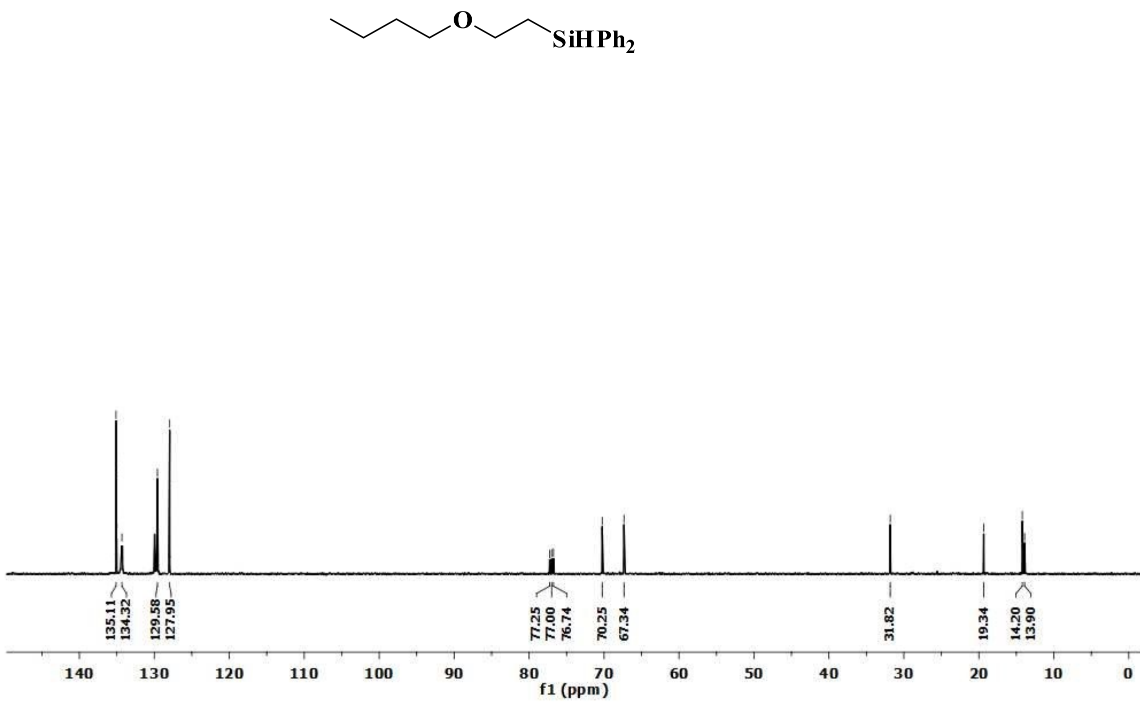

Fig. S24 ^{29}Si NMR spectrum ($CDCl_3$) of (2-butoxyethyl)diphenylsilane.

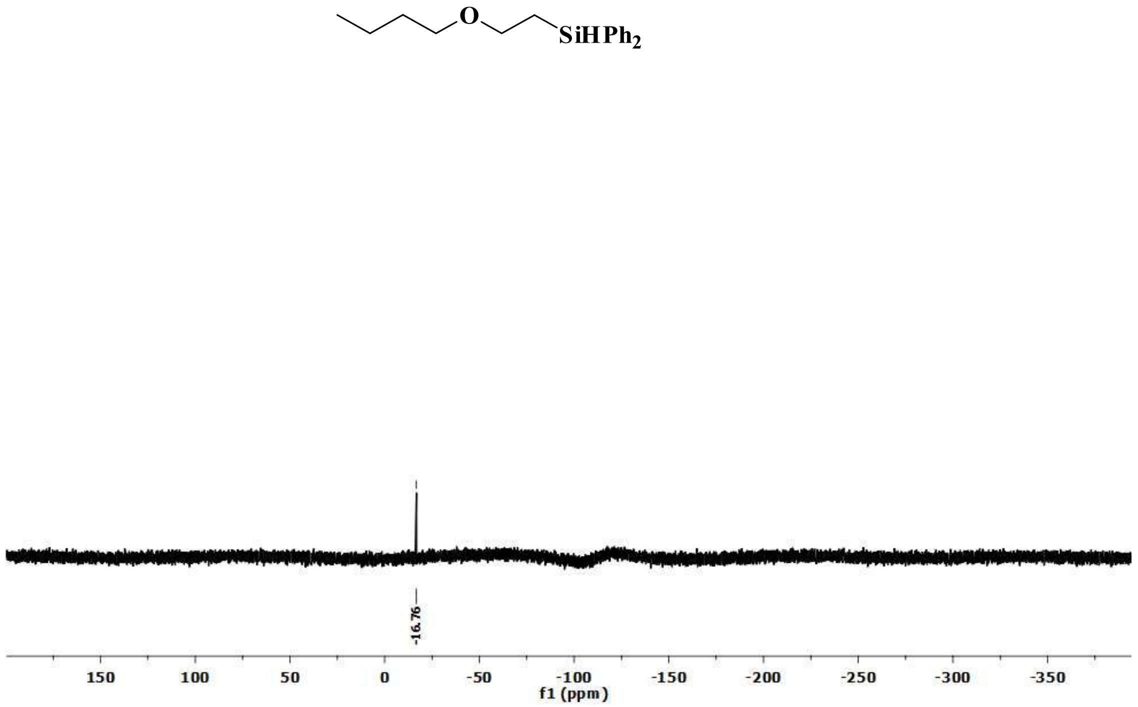

Fig. S25 ^{1}H NMR spectrum ($CDCl_3$) of (2-(cyclohexyloxy)ethyl)diphenylsilane.

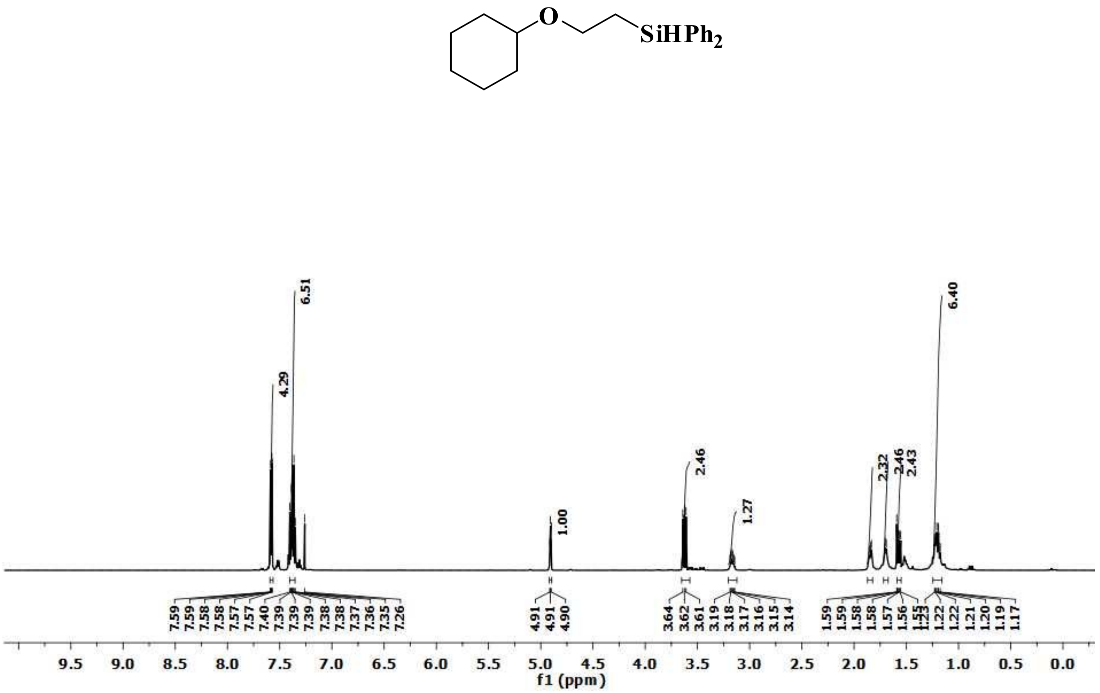

Fig. S26 ^{13}C NMR spectrum ($CDCl_3$) of (2-(cyclohexyloxy)ethyl)diphenylsilane.

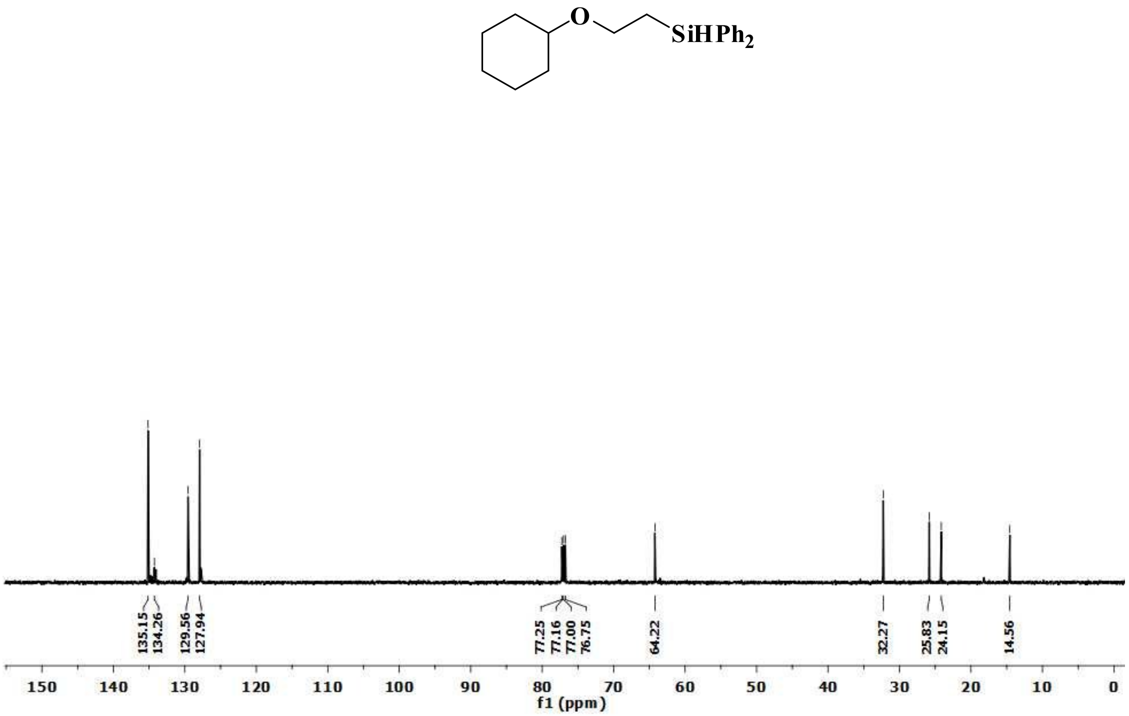

Fig. S27 ^{29}Si NMR spectrum ($CDCl_3$) of (2-(cyclohexyloxy)ethyl)diphenylsilane.

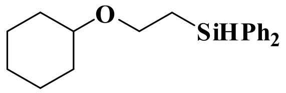

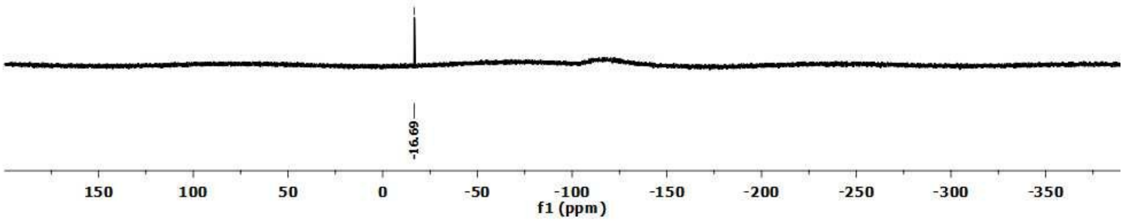

Fig. S28 ^{1}H NMR spectrum ($CDCl_3$) of (3-phenylpropyl)diphenylsiIane.

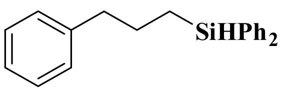

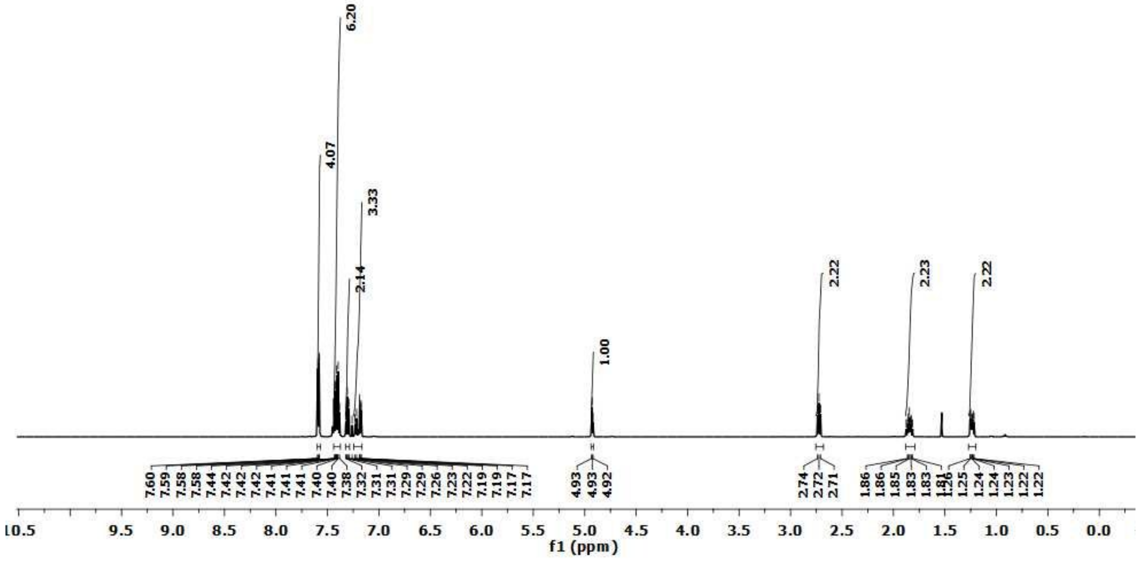

Fig. S29 ^{13}C NMR spectrum ($CDCl_3$) of (3-phenylpropyl)diphenylsiIane.

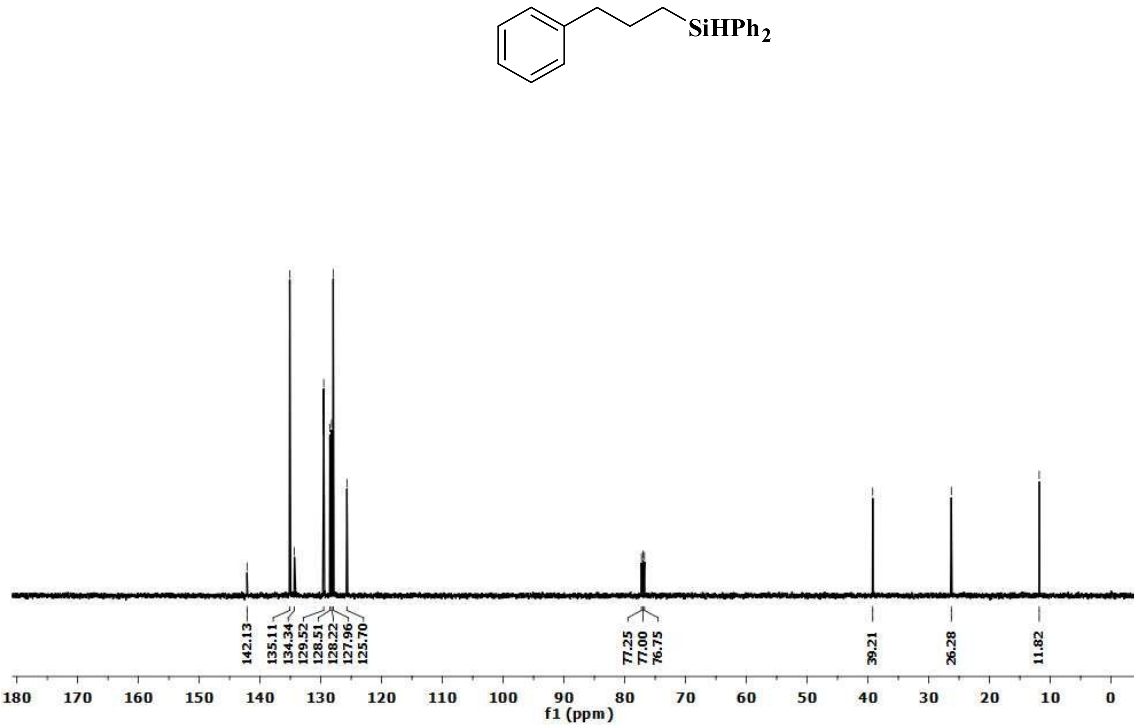

Fig. S30 ^{29}Si NMR spectrum ($CDCl_3$) of (3-phenylpropyl)diphenylsiIane.

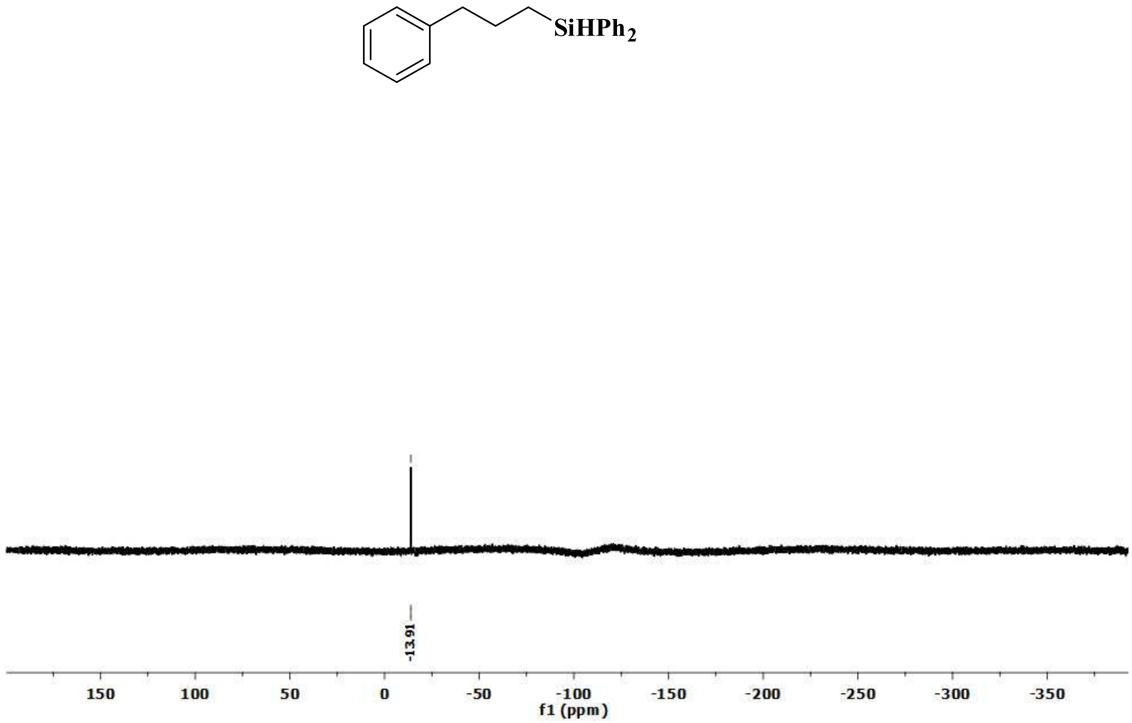

Fig. S31 ^{1}H NMR spectrum ($CDCl_3$) of (3-(4-methoxyphenyl)propyl)diphenylsilane.

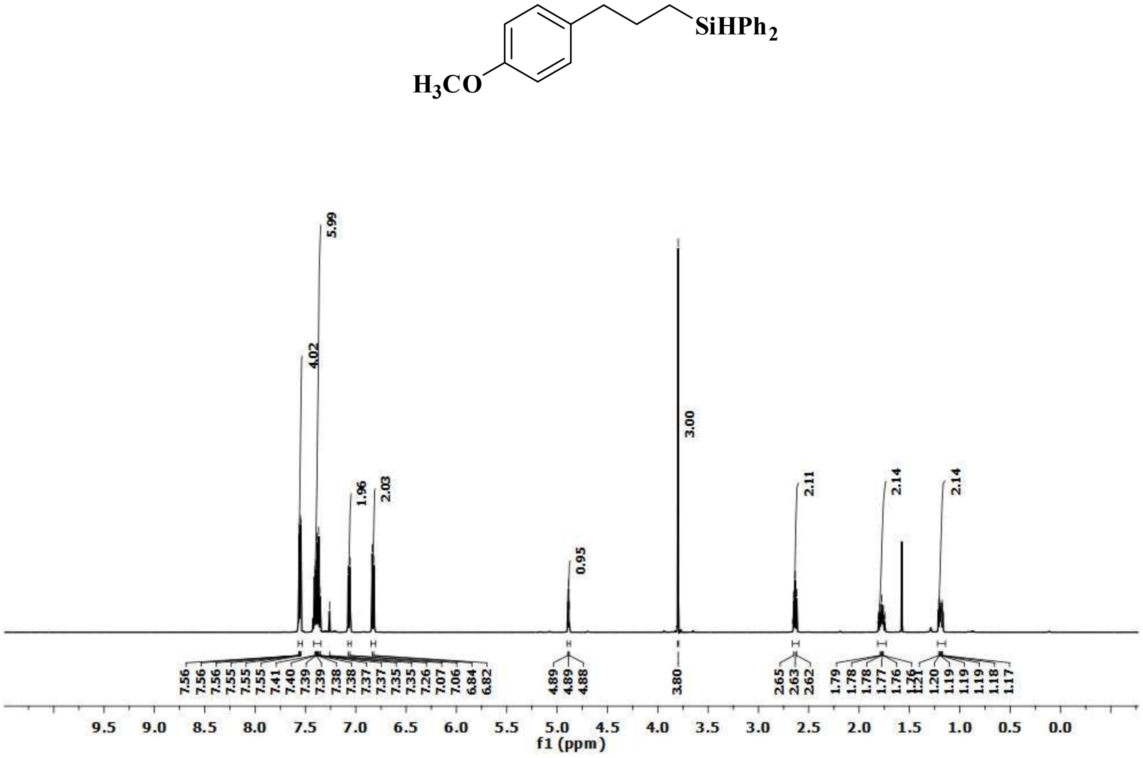

Fig. S32 ^{13}C NMR spectrum ($CDCl_3$) of (3-(4-methoxyphenyl)propyl)diphenylsilane.

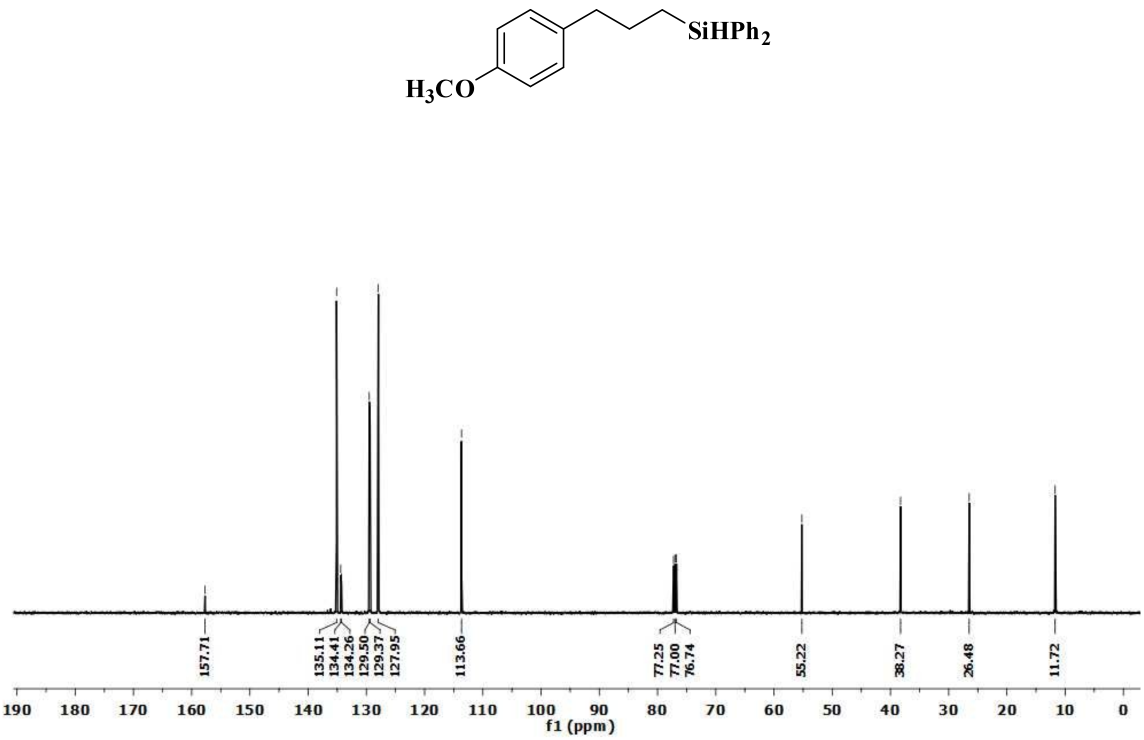

Fig. S33 ^{29}Si NMR spectrum ($CDCl_3$) of (3-(4-methoxyphenyl)propyl)diphenylsilane.

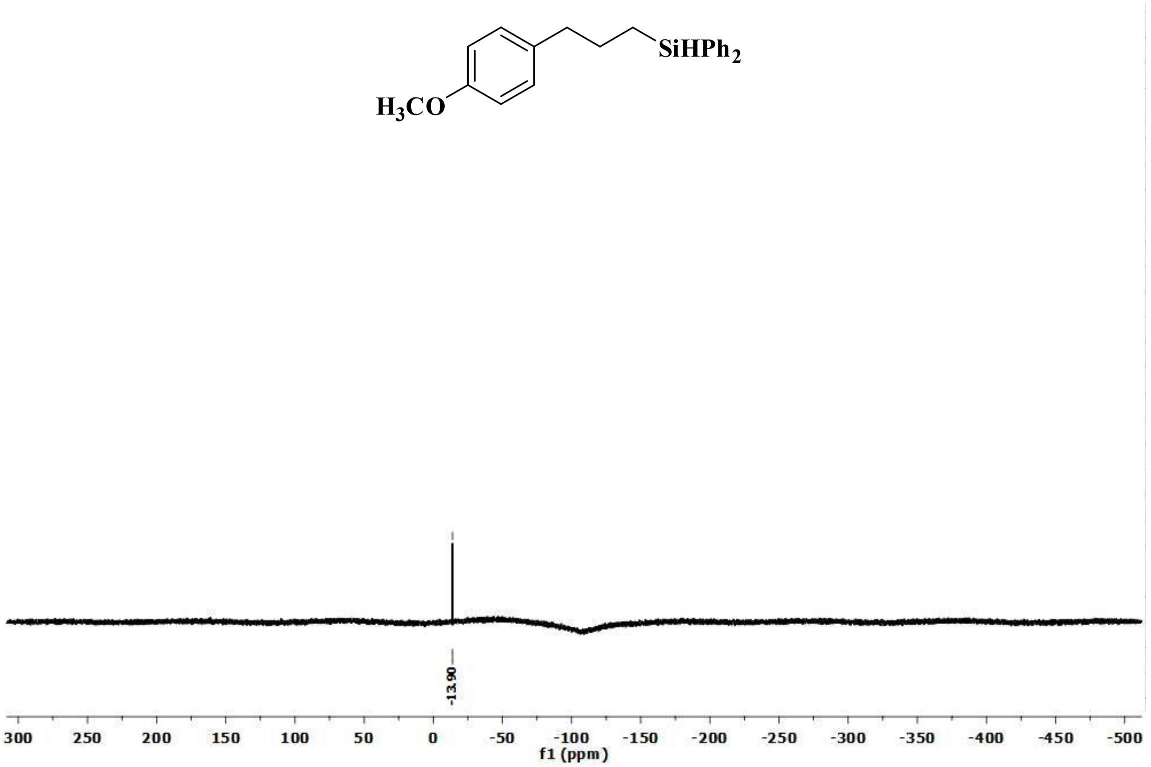

Fig. S34 ^{1}H NMR spectrum ($CDCl_3$) of (3-phenoxypropyl)diphenylsilane.

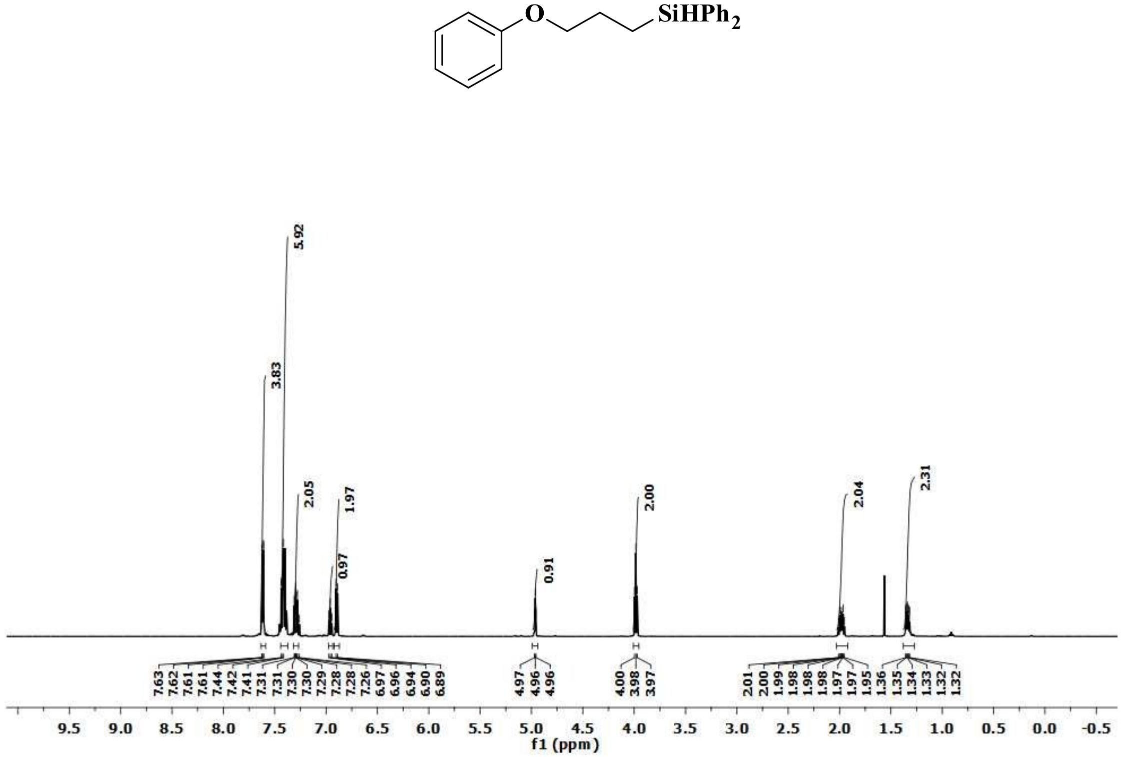

Fig. S35 ^{13}C NMR spectrum ($CDCl_3$) of (3-phenoxypropyl)diphenylsilane.

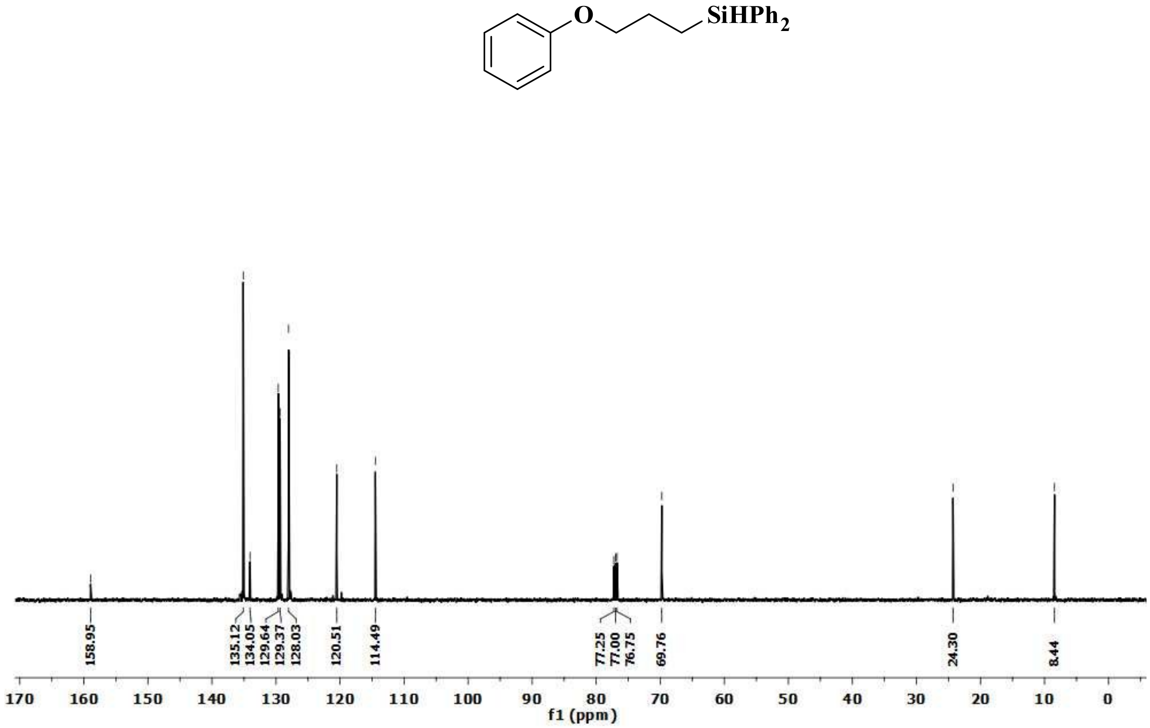

Fig. S36 ^{29}Si NMR spectrum ($CDCl_3$) of (3-phenoxypropyl)diphenylsilane.

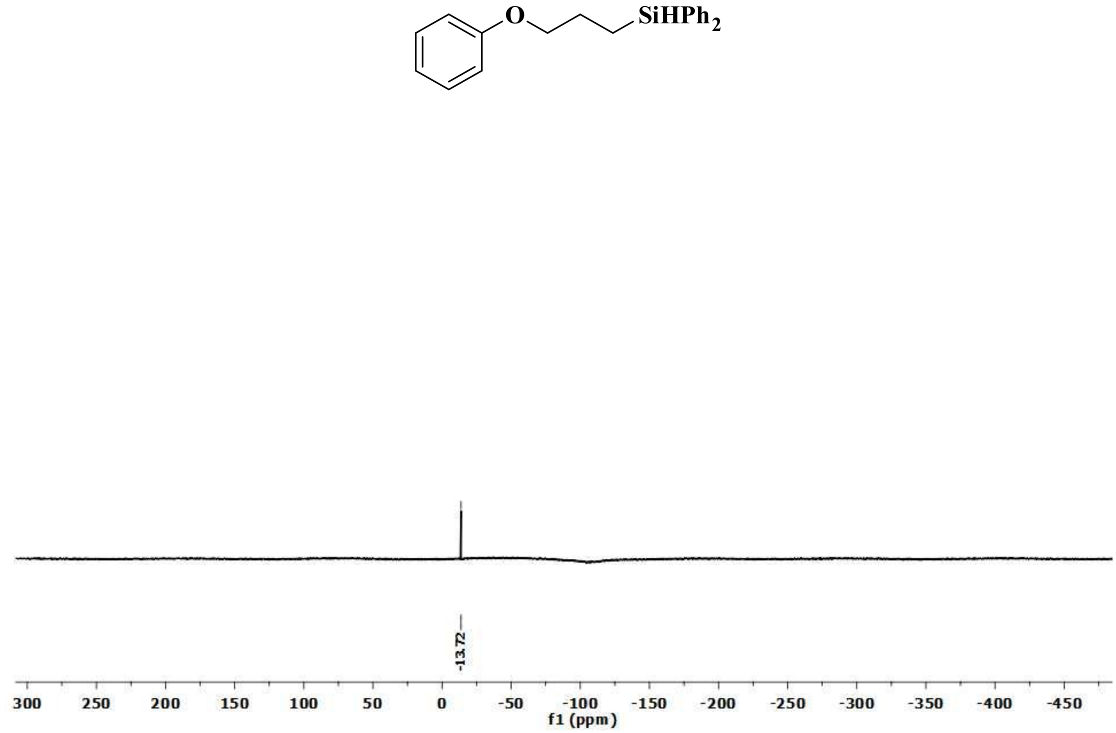

Fig. S37 ^{1}H NMR spectrum ($CDCl_3$) of 5-(diphenylsilyl)pentyl benzoate.

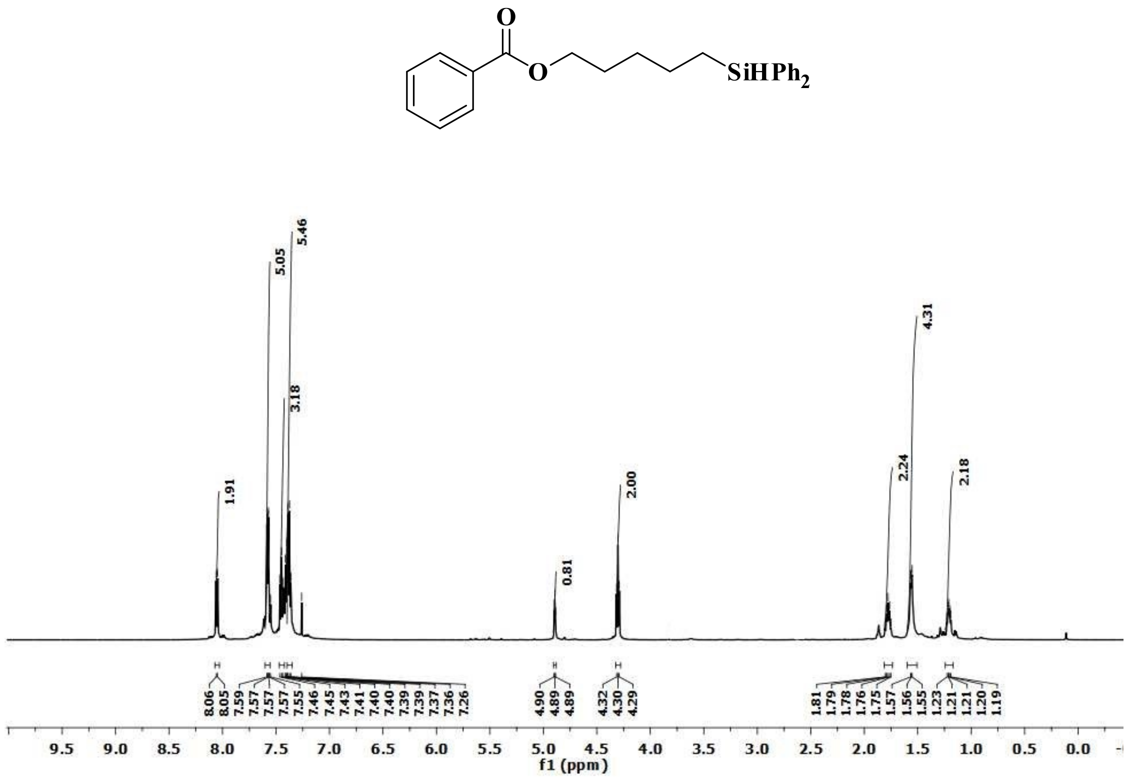

Fig.S38 ^{13}C NMR spectrum ($CDCl_3$) of 5-(diphenylsilyl)pentyl benzoate.

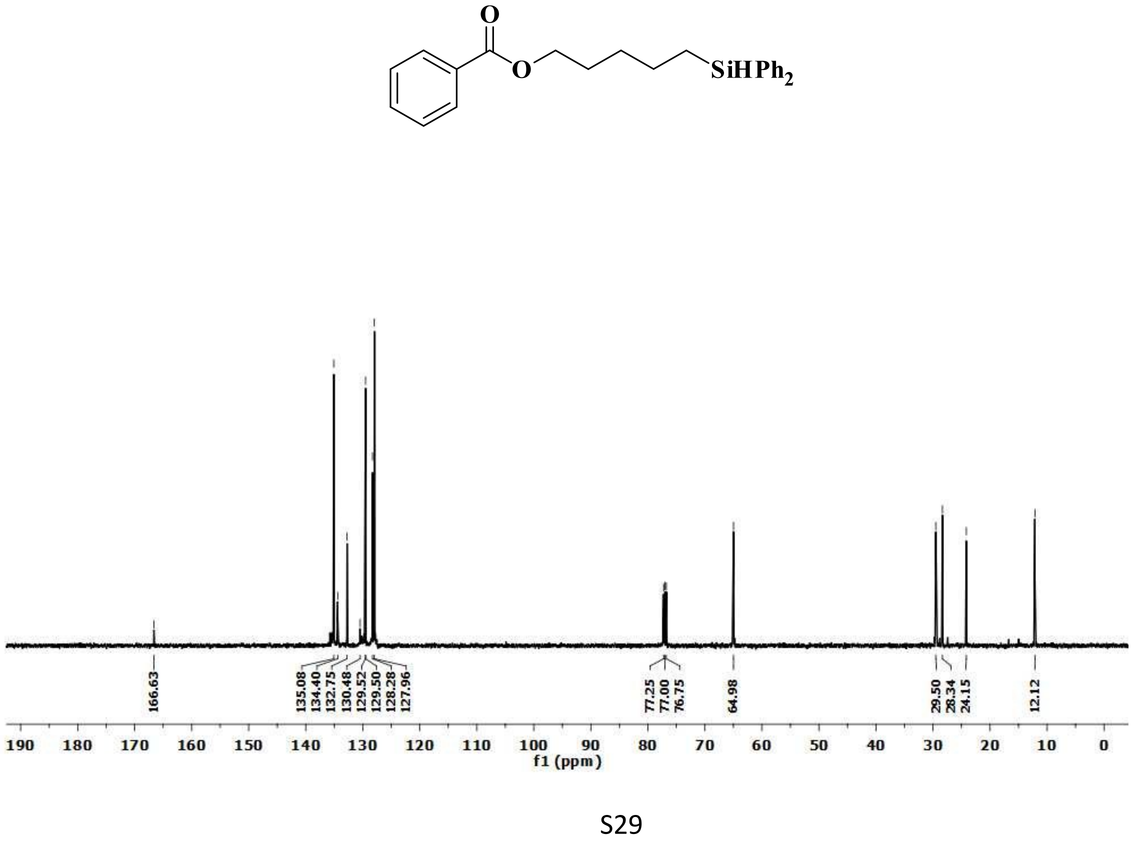

Fig. S39 ^{29}Si NMR spectrum ($CDCl_3$) of 5-(diphenylsilyl)pentyl benzoate.

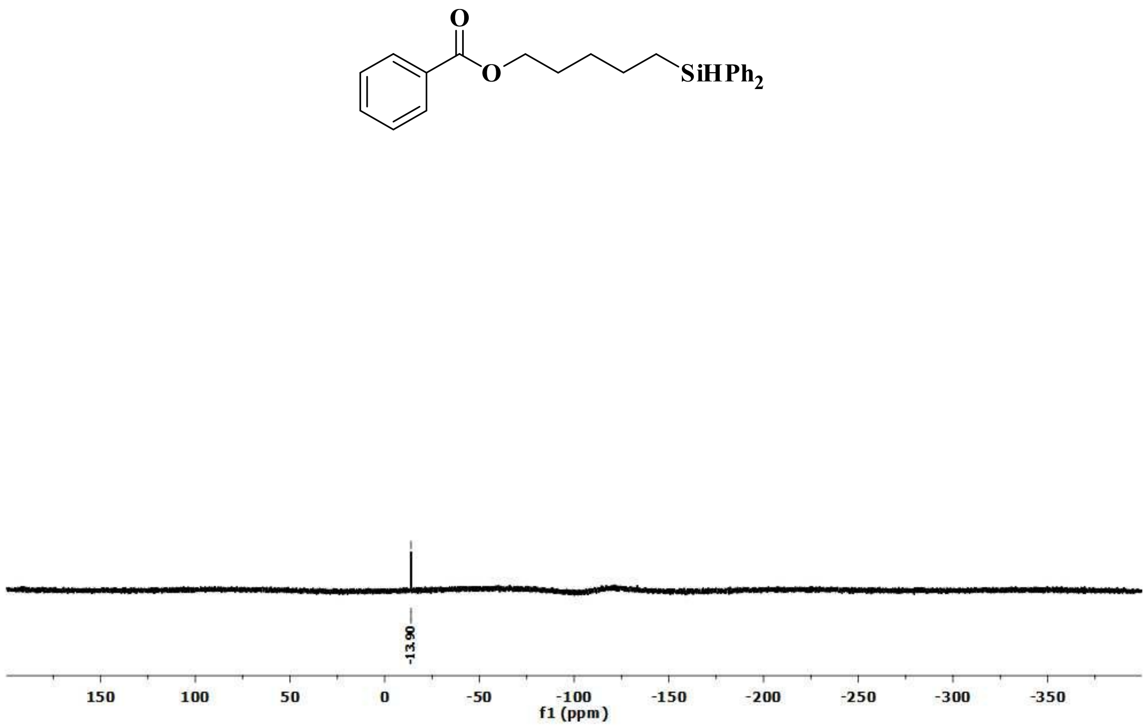

Fig. S40 ^{1}H NMR spectrum ($CDCl_3$) of 1-(5-(diphenylsilyl)pentyl)-1H-indole.

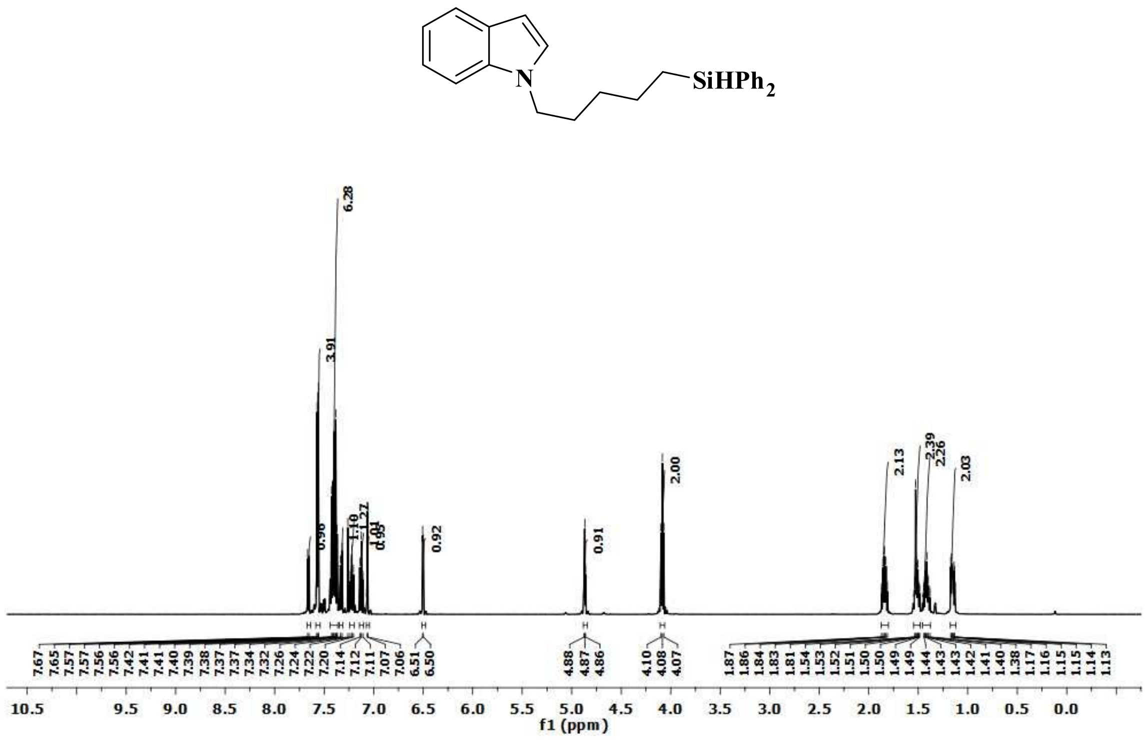

Fig. S41 ^{13}C NMR spectrum ($CDCl_3$) of 1-(5-(diphenylsilyl)pentyl)-1H-indole.

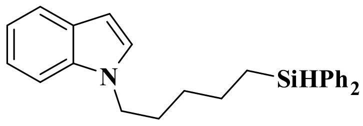

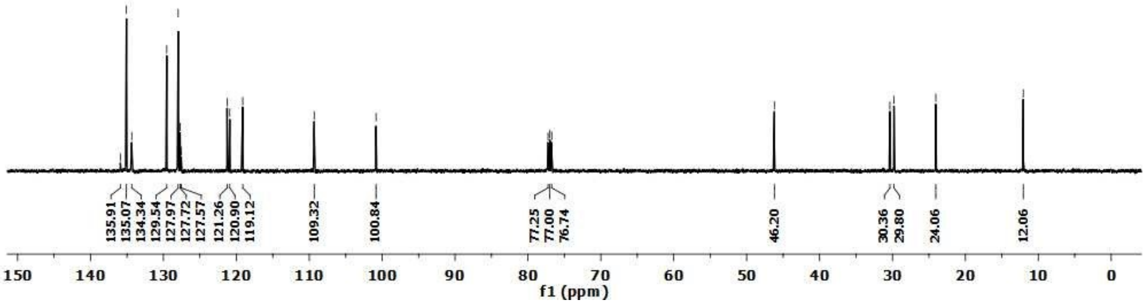

Fig. S42 ^{29}Si NMR spectrum ($CDCl_3$) of 1-(5-(diphenylsilyl)pentyl)-1H-indole.

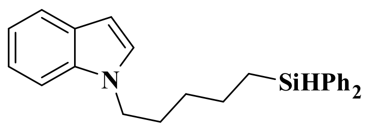

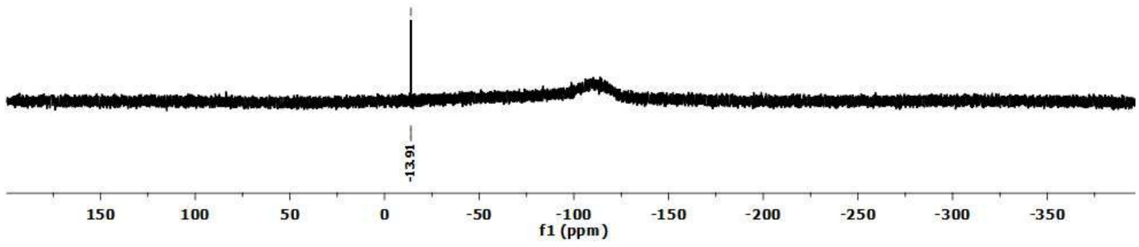

Fig. S43 ^{1}H NMR spectrum ($CDCl_3$) of 9-(5-(diphenylsilyl)pentyl)-9H-carbazole.

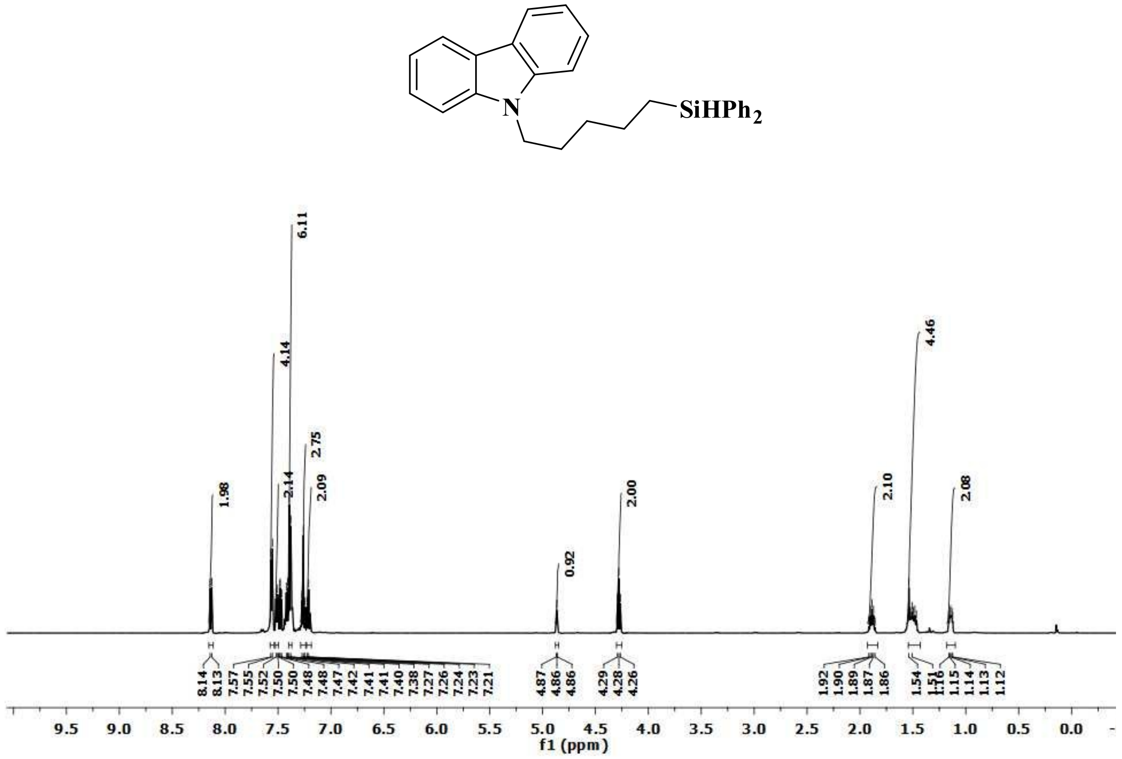

Fig. S44 ^{13}C NMR spectrum ($CDCl_3$) of 9-(5-(diphenylsilyl)pentyl)-9H-carbazole.

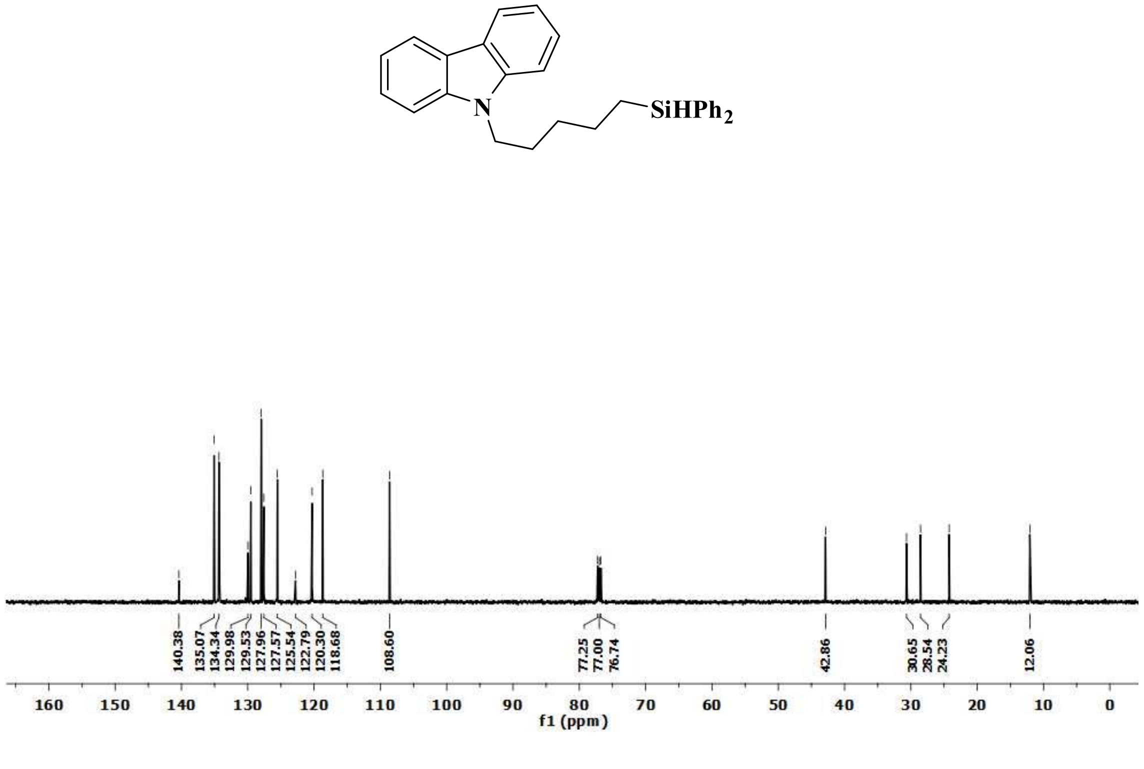

Fig. S45 ^{29}Si NMR spectrum ($CDCl_3$) of 9-(5-(diphenylsilyl)pentyl)-9H-carbazole.

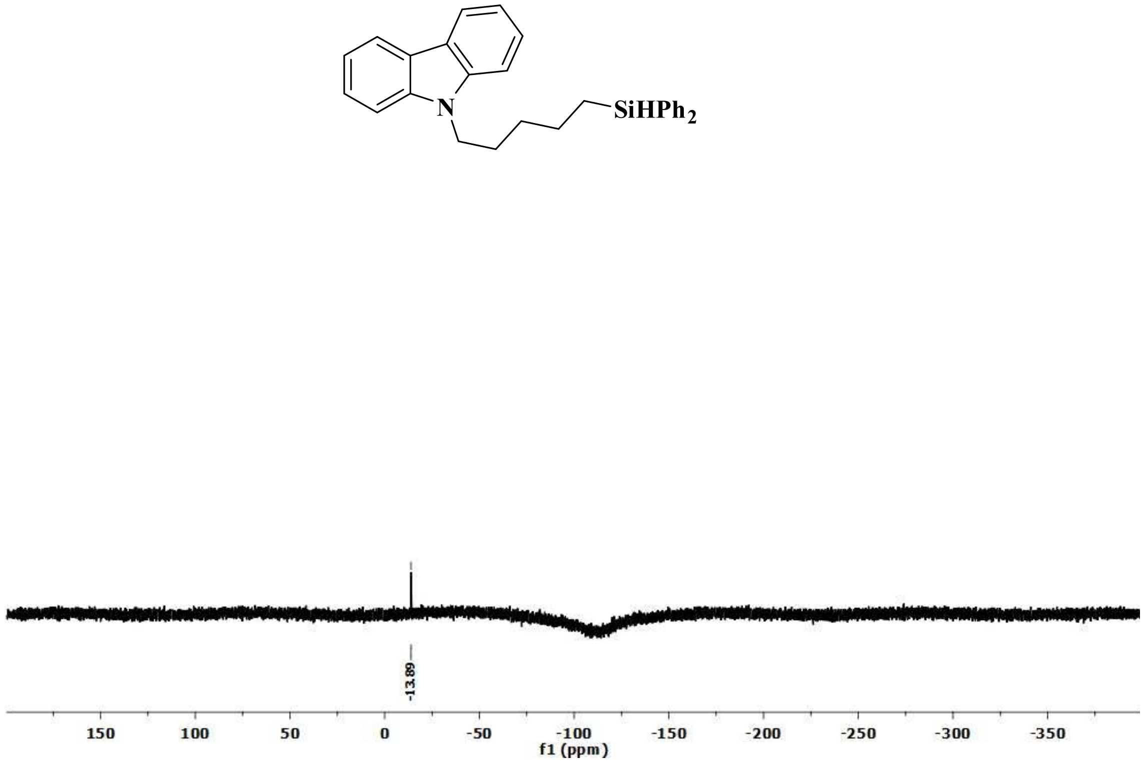

Fig. S46 ^{1}H NMR spectrum ($CDCl_3$) of 6-((6-(diphenylsilyl)pentyl)oxy)-4-methyl-2H-chromen-2-one.

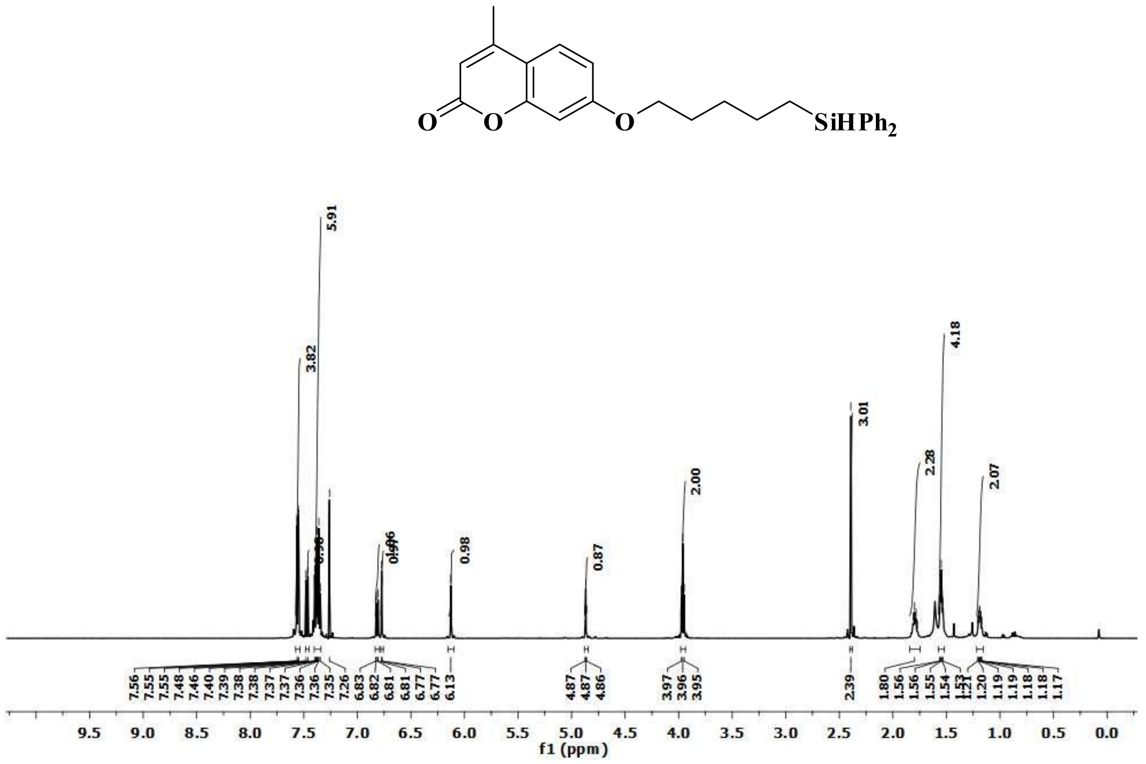

Fig. S47 ^{13}C NMR spectrum ($CDCl_3$) of 6-((6-(diphenylsilyl)pentyl)oxy)-4-methyl-2H-chromen-2-one.

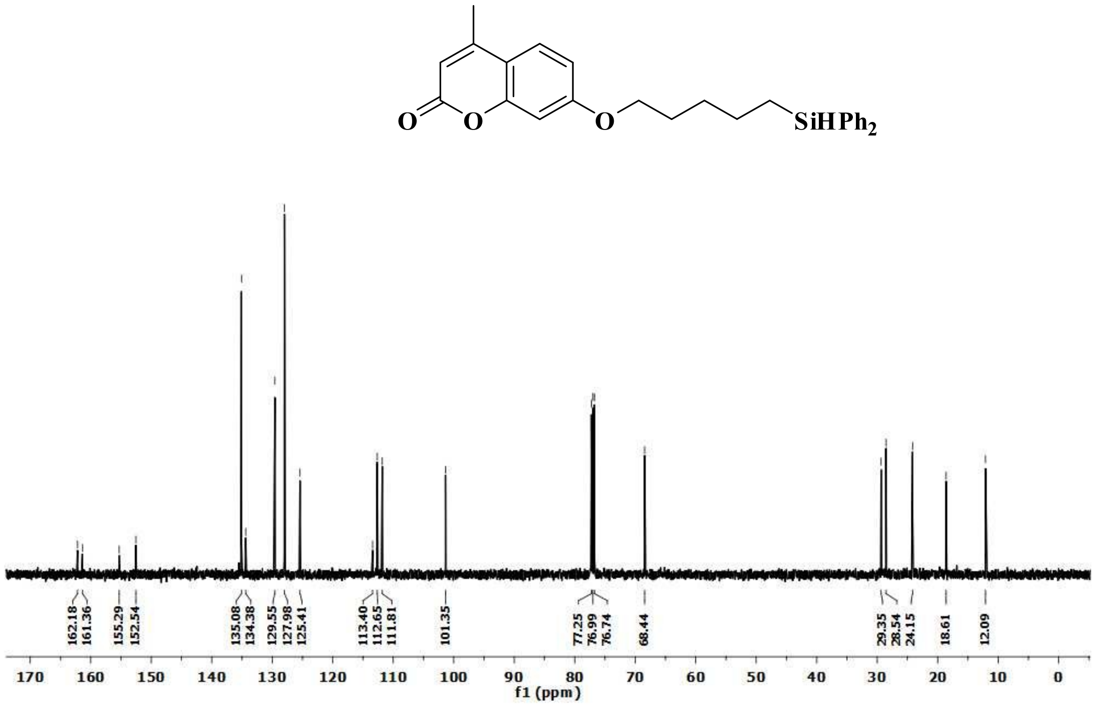

Fig. S48 ^{29}Si NMR spectrum ($CDCl_3$) of 6-((6-(diphenylsilyl)pentyl)oxy)-4-methyl-2H-chromen-2-one.

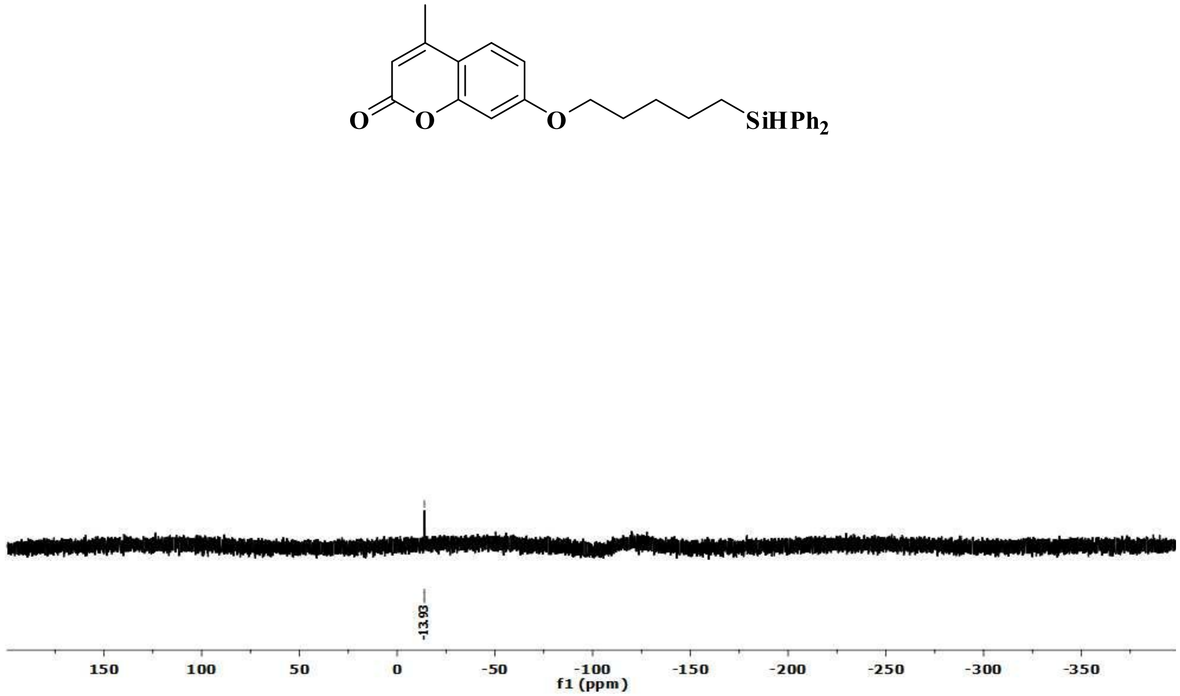

Fig. S49 ^{1}H NMR spectrum ($CDCl_3$) of dioctyl(phenyl)silane.

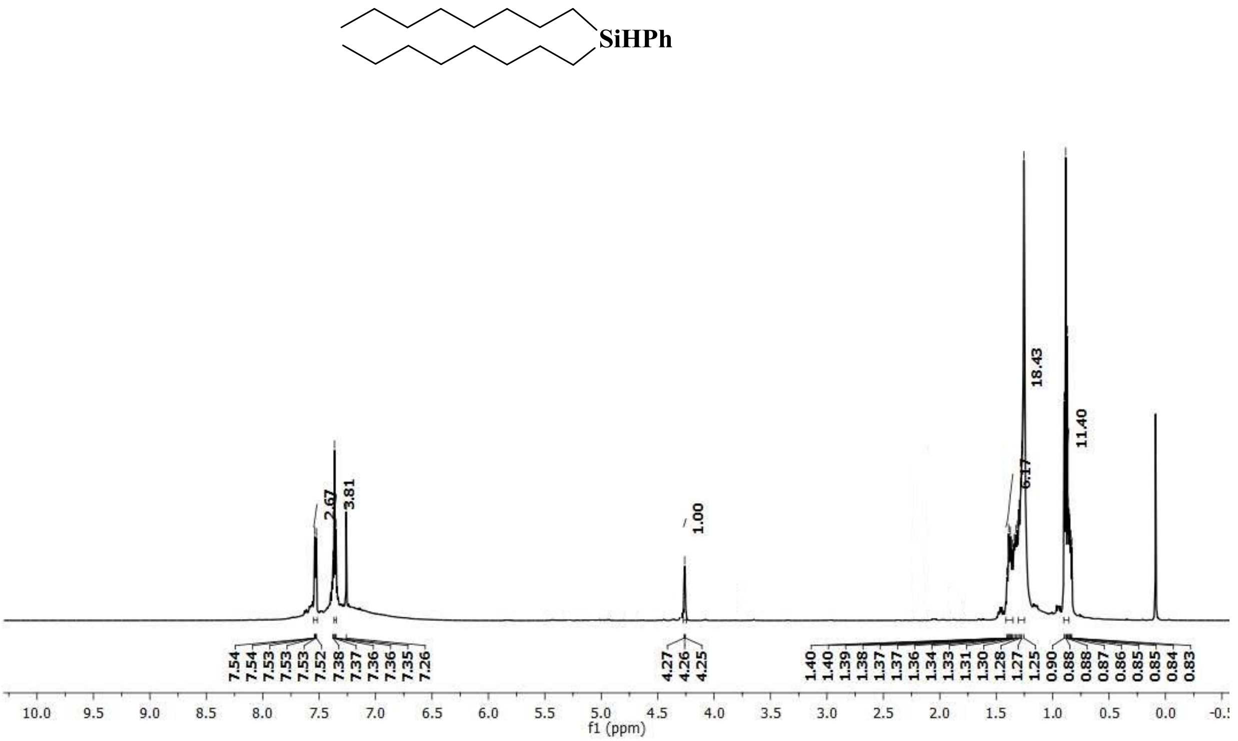

Fig. S50 ^{13}C NMR spectrum ($CDCl_3$) of dioctyl(phenyl)silane.

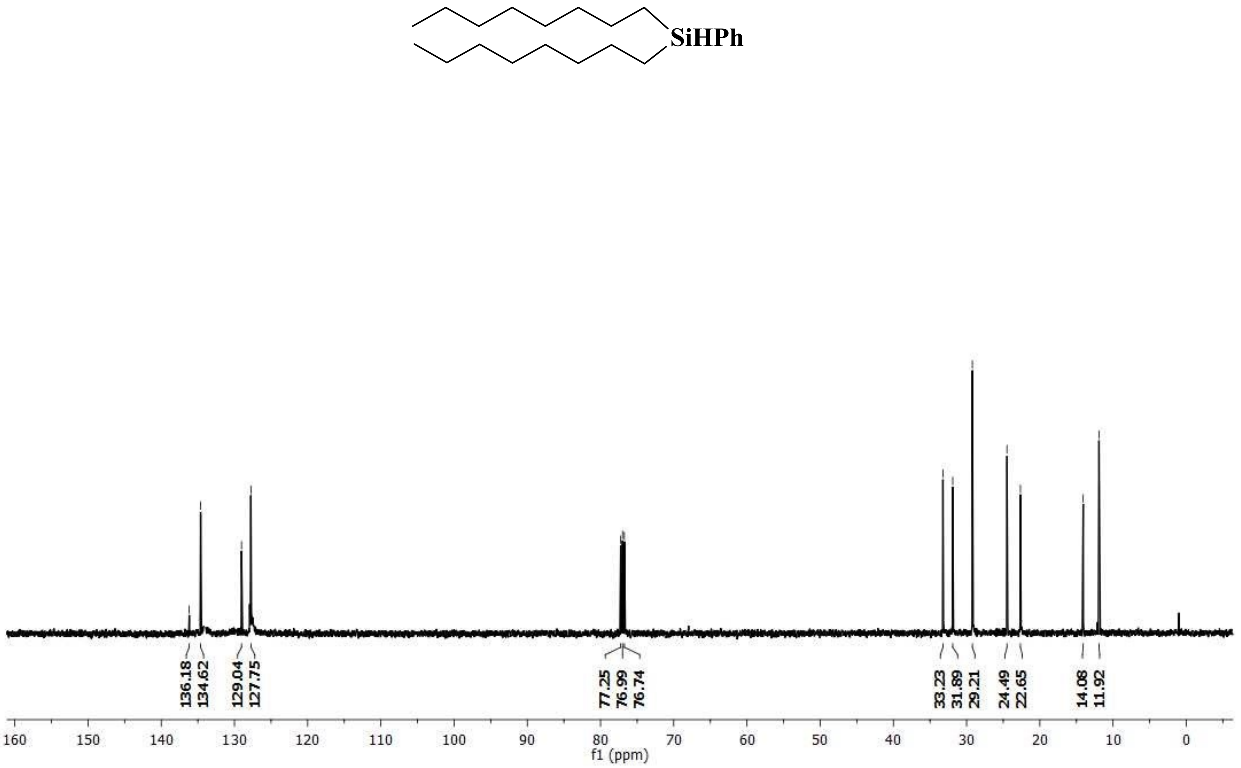

Fig. S51 ^{29}Si NMR spectrum ($CDCl_3$) of dioctyl(phenyl)silane.

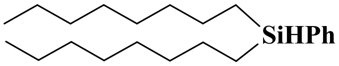

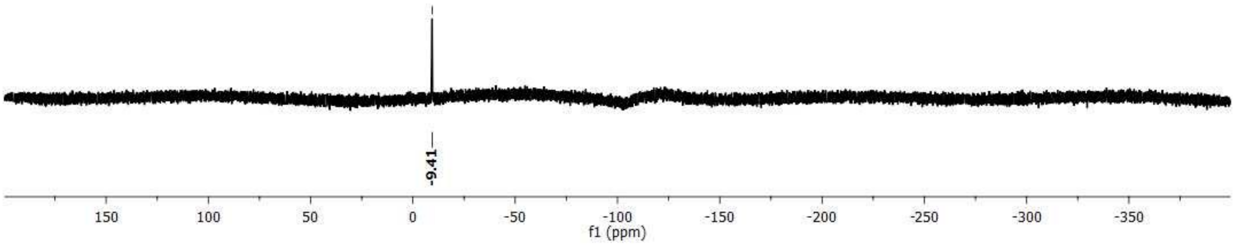

Fig. S52 ^{1}H NMR spectrum ($CDCl_3$) of bis(5-bromopentyl)(phenyl)silane.

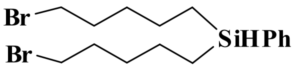

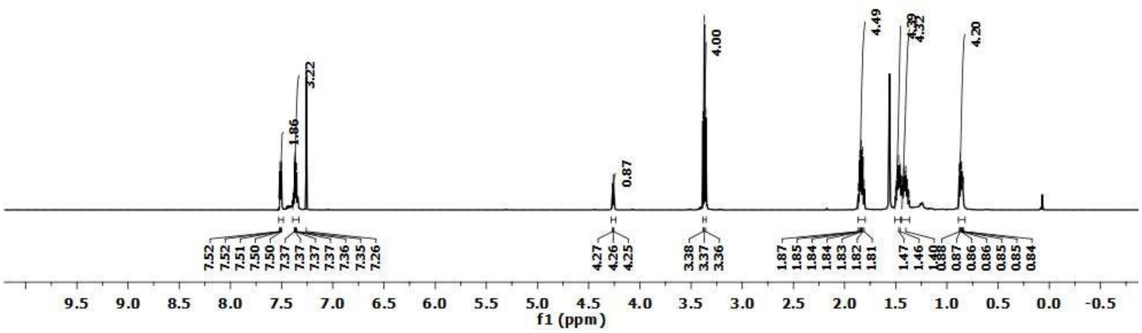

Fig. S53 ^{13}C NMR spectrum ($CDCl_3$) of bis(5-bromopentyl)(phenyl)silane.

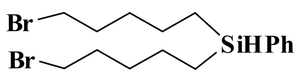

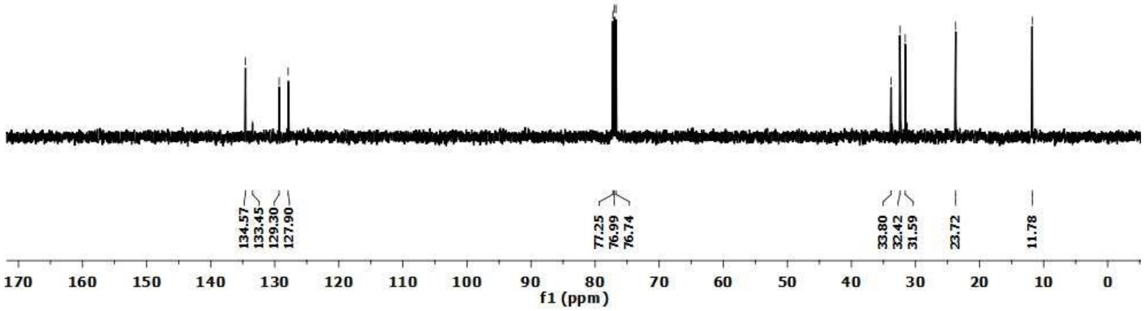

Fig. S54 ^{29}Si NMR spectrum ($CDCl_3$) of bis(5-bromopentyl)(phenyl)silane.

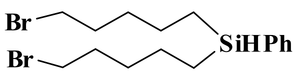

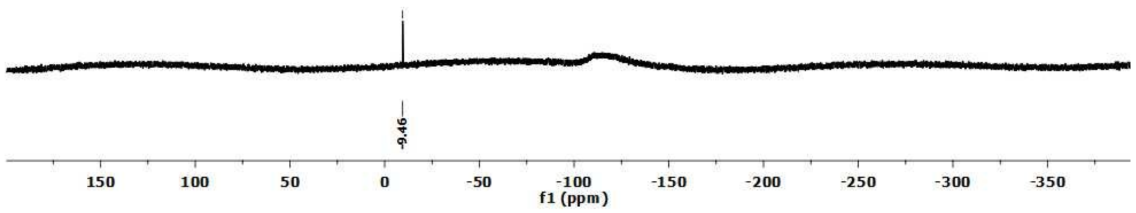

Fig. S55 ^{1}H NMR spectrum ($CDCl_3$) of bis(5-bromopentyl)(butyl)silane

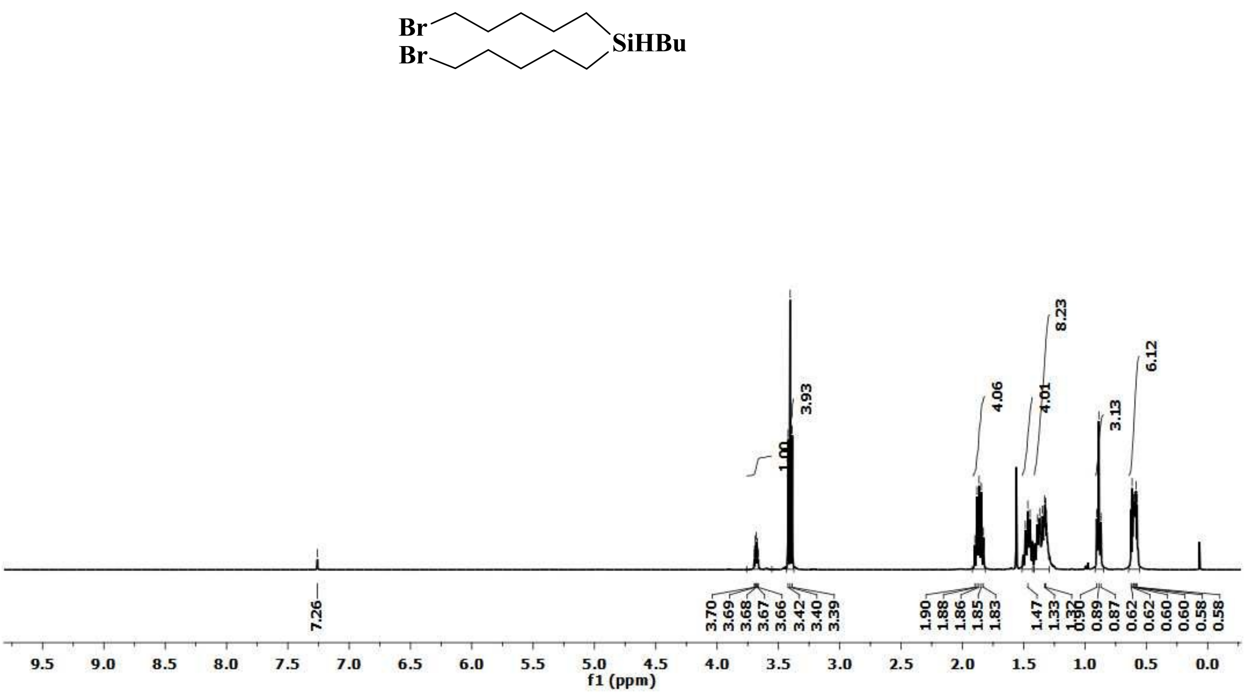

Fig. S56 ^{13}C NMR spectrum ($CDCl_3$) of bis(5-bromopentyl)(butyl)silane

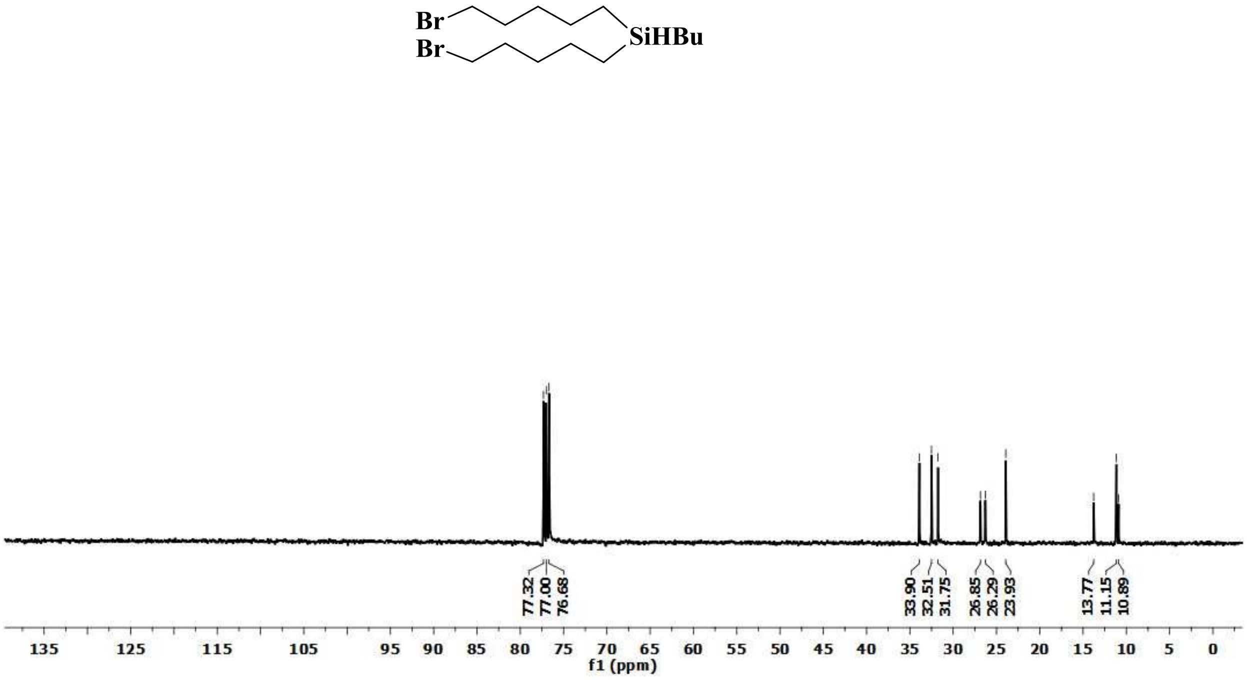

Fig. S57 ^{29}Si NMR spectrum ($CDCl_3$) of bis(5-bromopentyl)(butyl)silane

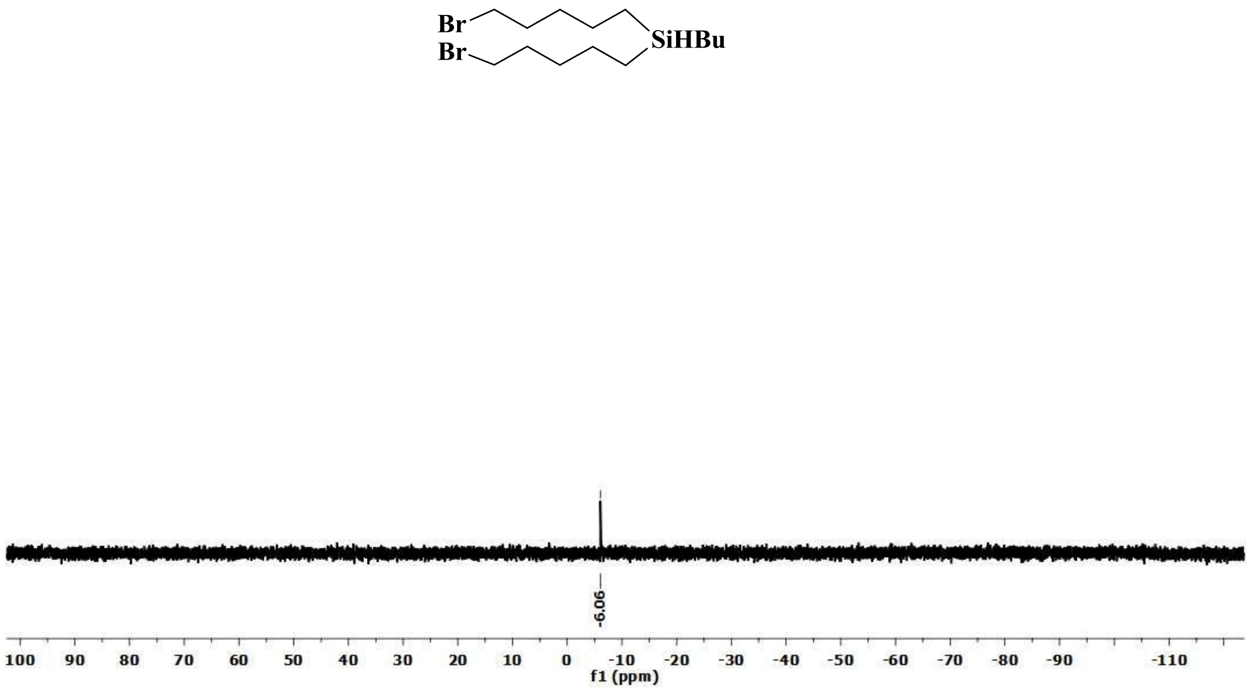

Fig. S58 ^{1}H NMR spectrum ($CDCl_3$) of bis(4-bromobutyl)(butyl)silane

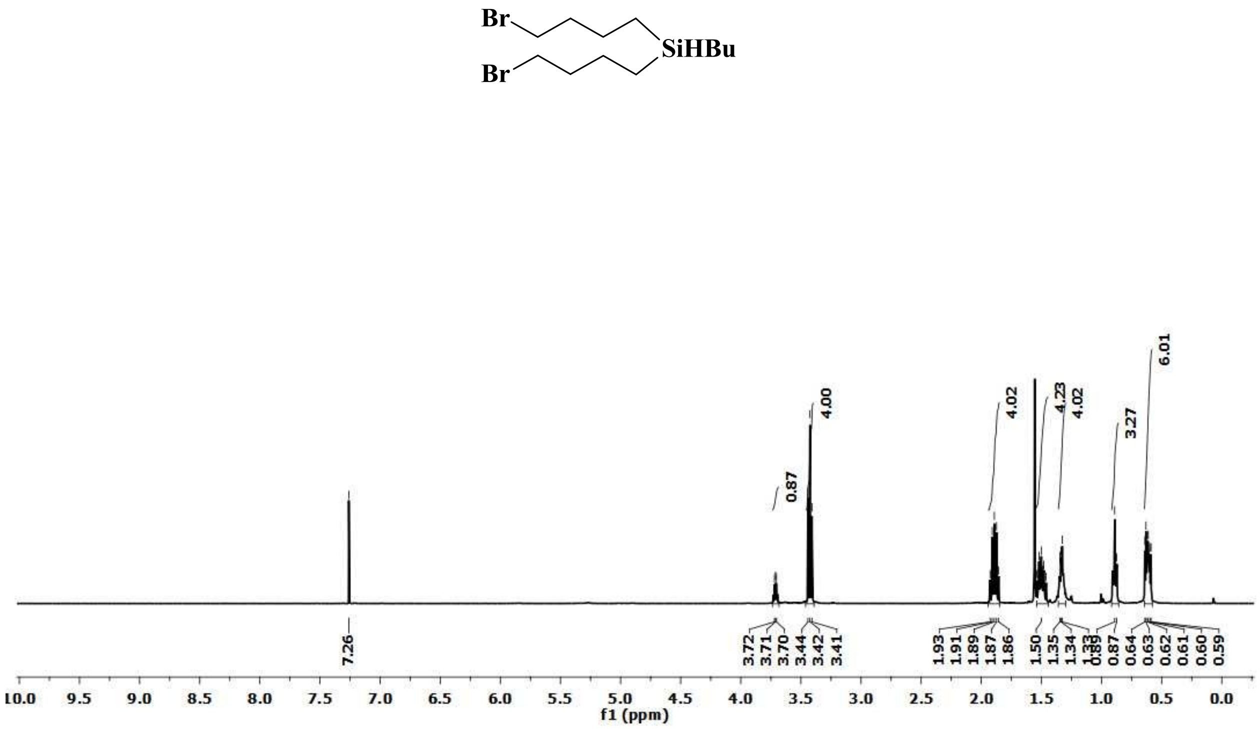

Fig. S59 ^{13}C NMR spectrum ($CDCl_3$) of bis(4-bromobutyl)(butyl)silane

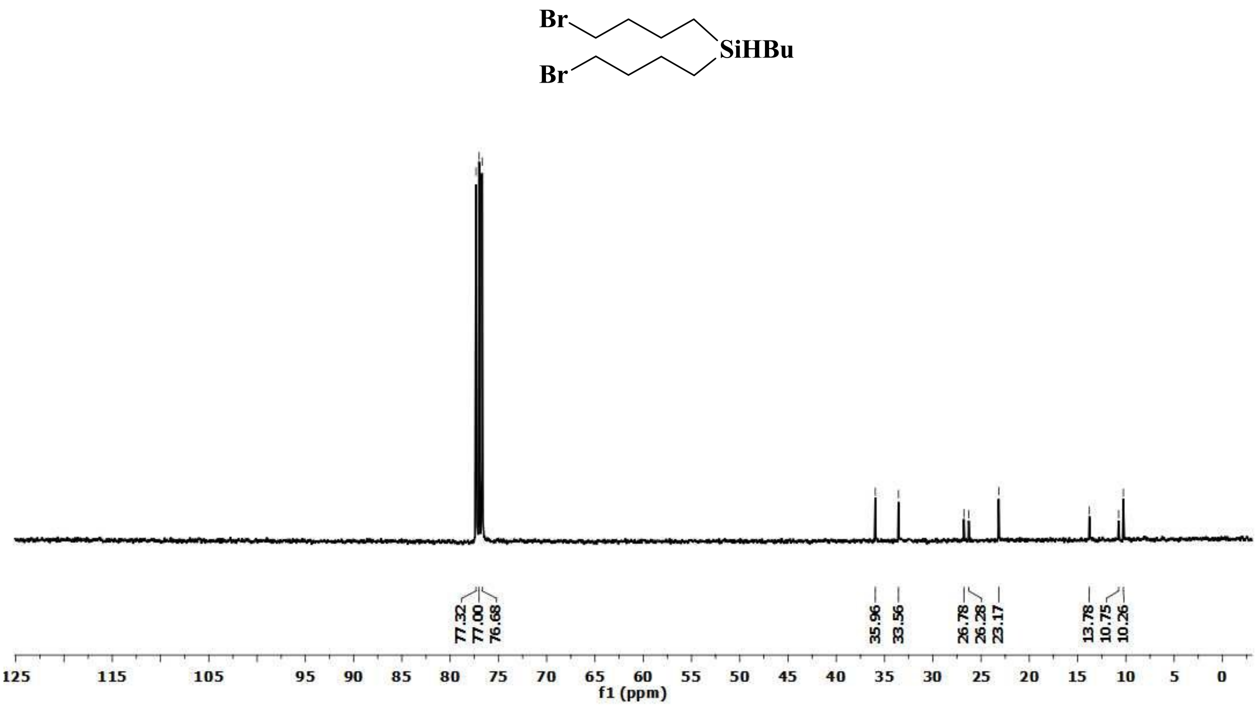

Fig. S60 ^{29}Si NMR spectrum ($CDCl_3$) of bis(4-bromobutyl)(butyl)silane

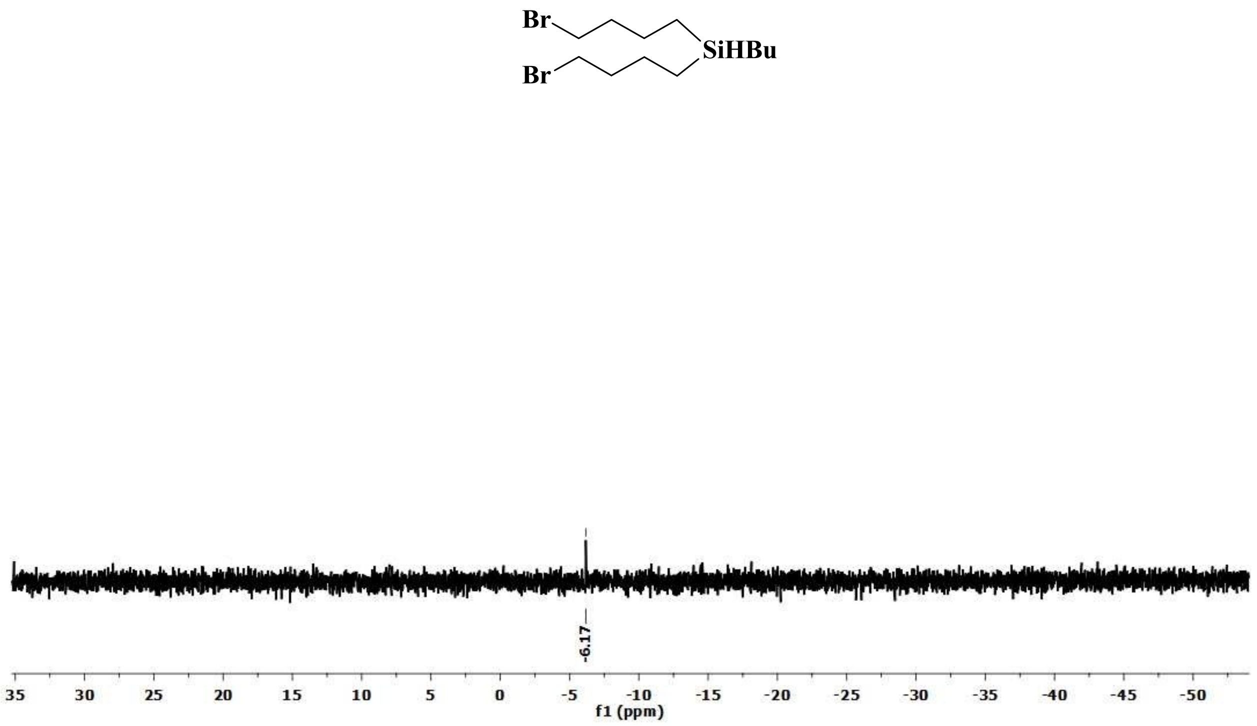

Fig. S61 ^{1}H NMR spectrum ($CDCl_3$) of (5-bromopentyl)(octyl)(phenyl)silane.

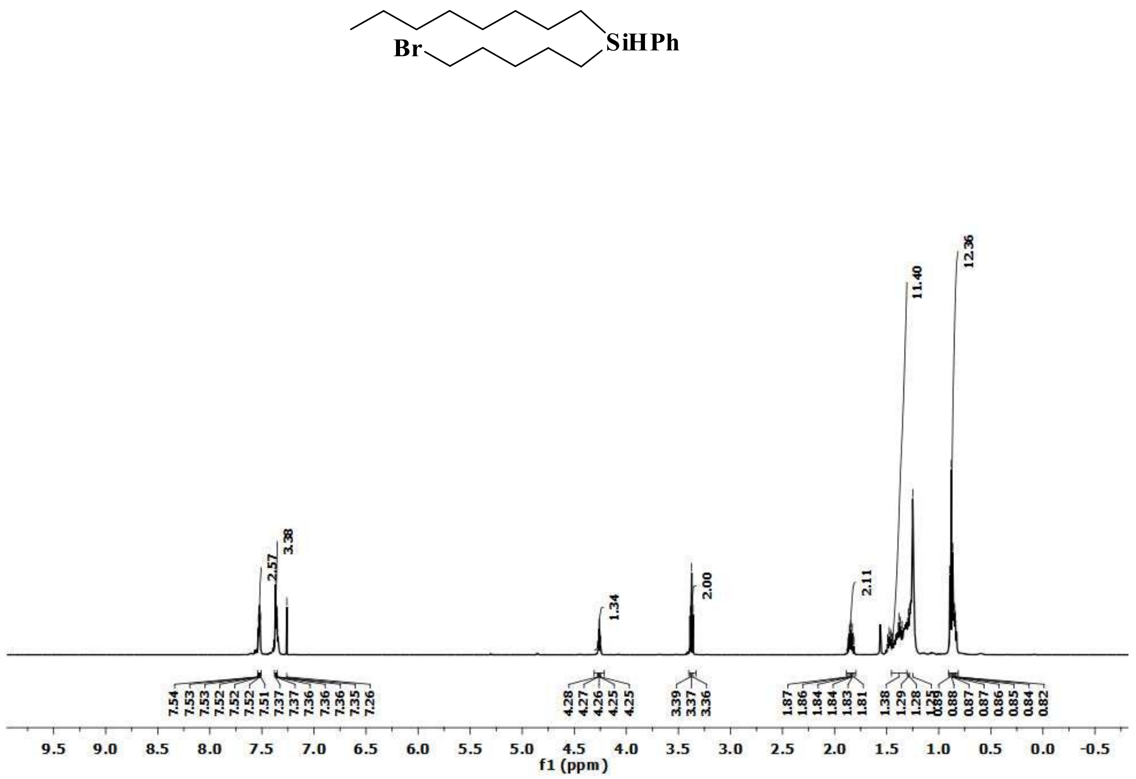

Fig. S62 ^{13}C NMR spectrum ($CDCl_3$) of (5-bromopentyl)(octyl)(phenyl)silane.

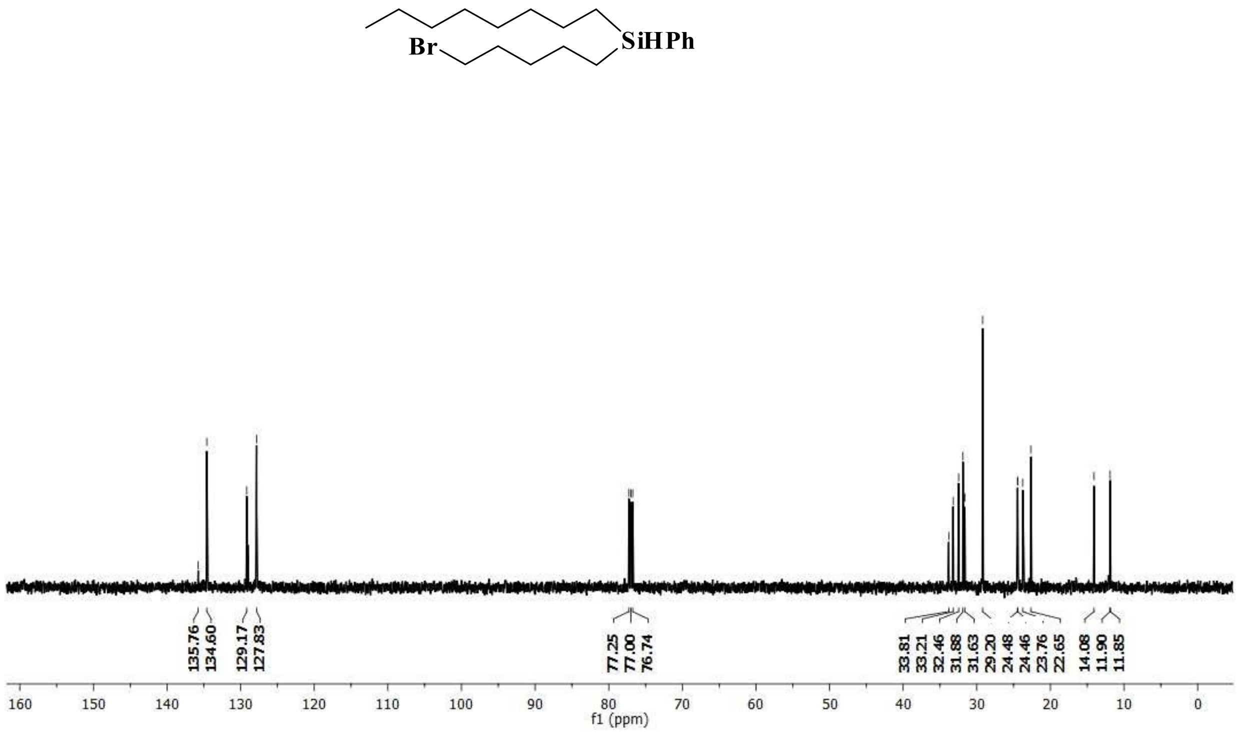

Fig. S63 ^{29}Si NMR spectrum ($CDCl_3$) of (5-bromopentyl)(octyl)(phenyl)silane.

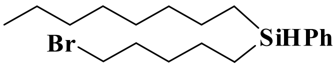

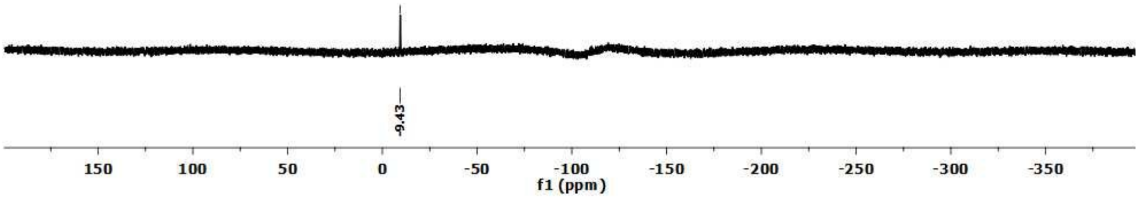

Fig. S64 ^{1}H NMR spectrum ($CDCl_3$) of (4-bromobutyl)(octyl)(phenyl)silane.

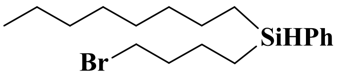

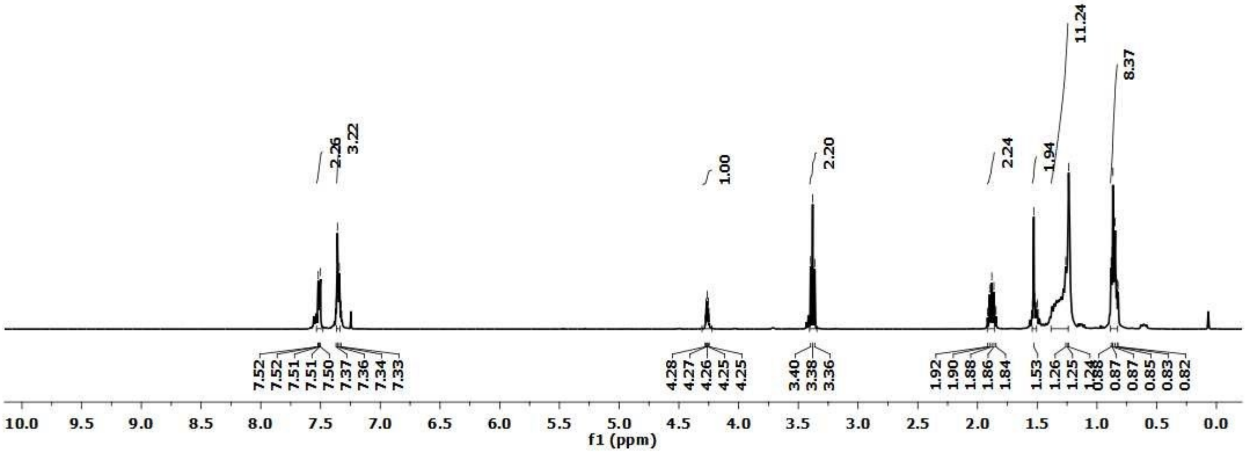

Fig. S65 ^{13}C NMR spectrum ($CDCl_3$) of (4-bromobutyl)(octyl)(phenyl)silane.

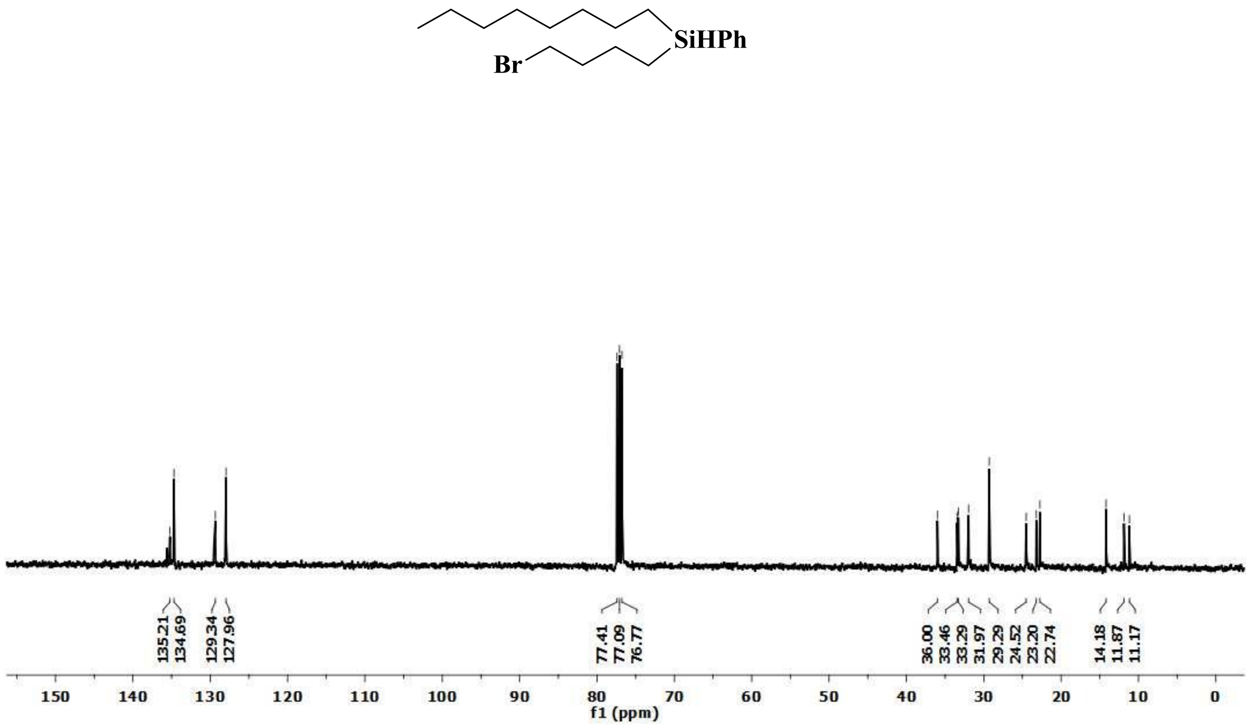

Fig. S66 ^{29}Si NMR spectrum ($CDCl_3$) of (4-bromobutyl)(octyl)(phenyl)silane.

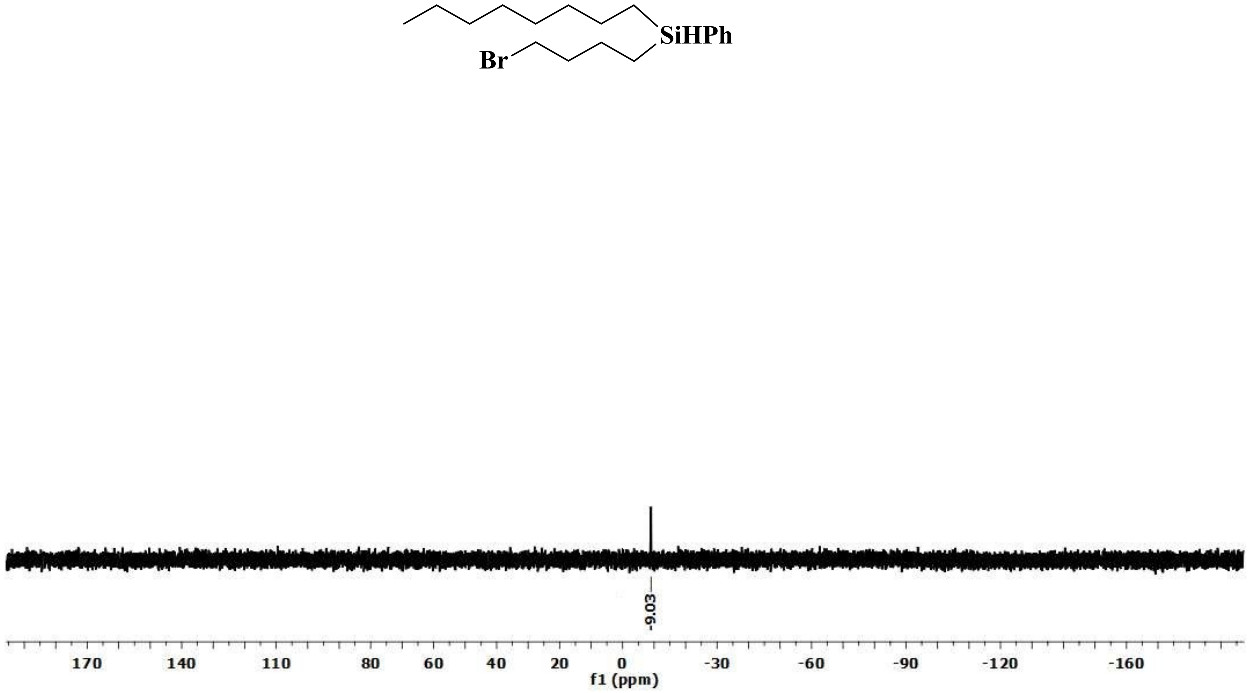

Fig. S67 ^{1}H NMR spectrum ($CDCl_3$) of (5-bromopentyl)(octyl)(butyl)silane.

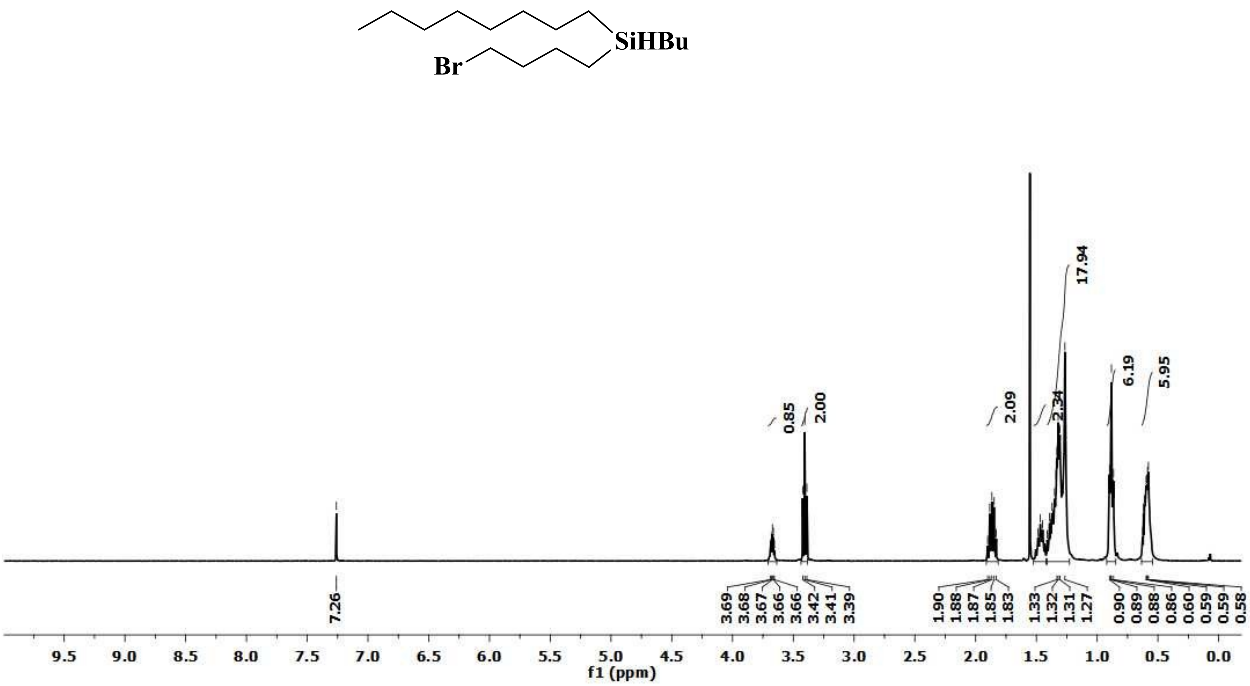

Fig. S68 ^{13}C NMR spectrum ($CDCl_3$) of (5-bromopentyl)(octyl)(butyl)silane.

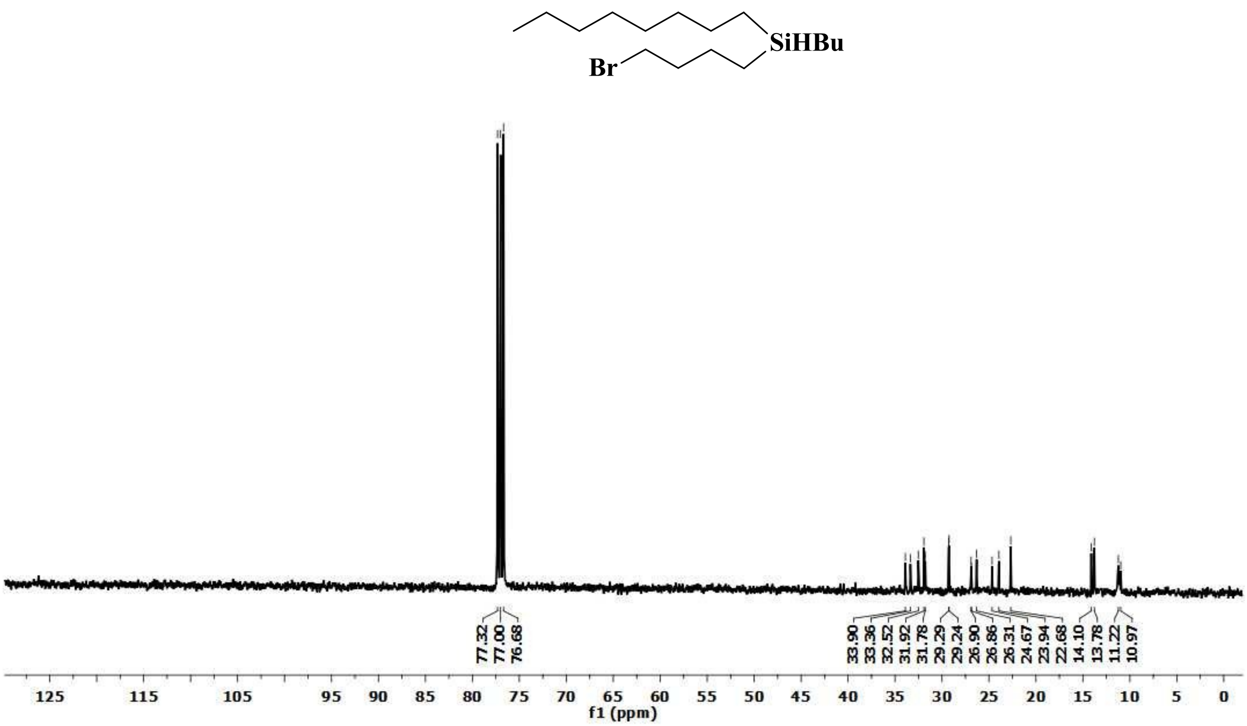

Fig. S69 ^{29}Si NMR spectrum ($CDCl_3$) of (5-bromopentyl)(octyl)(butyl)silane.

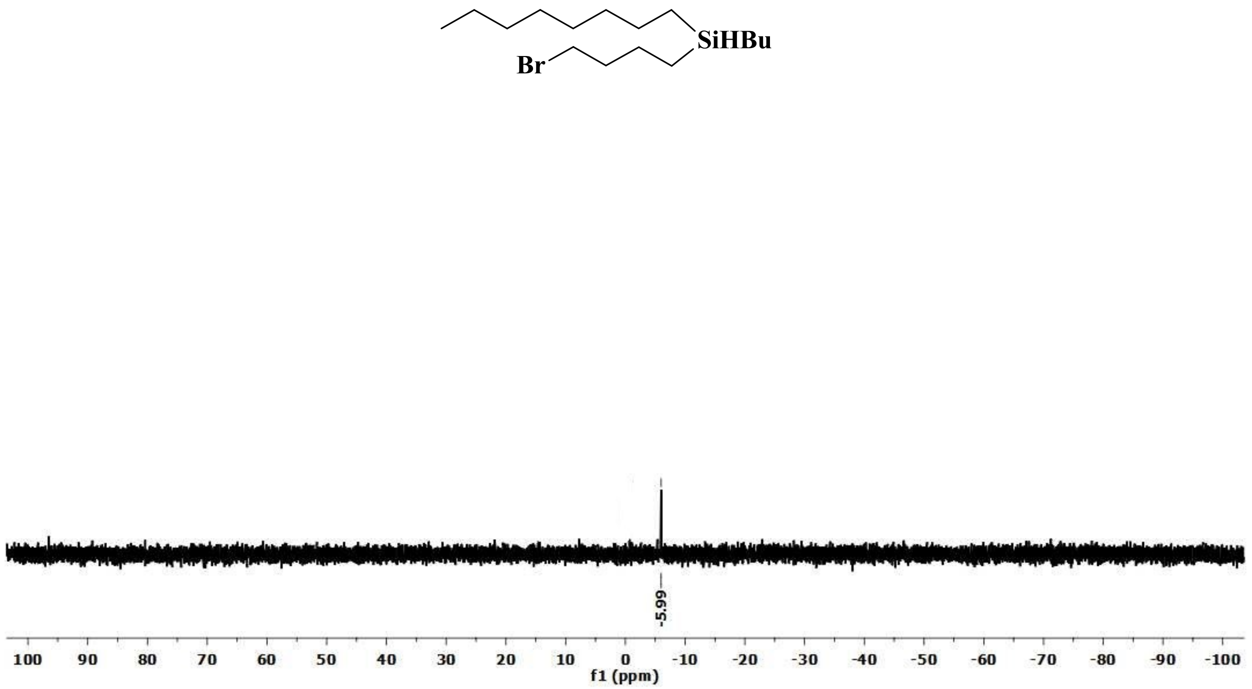

Fig. S70 ^{1}H NMR spectrum ($CDCl_3$) of (4-bromobutyl)(octyl)(butyl)silane.

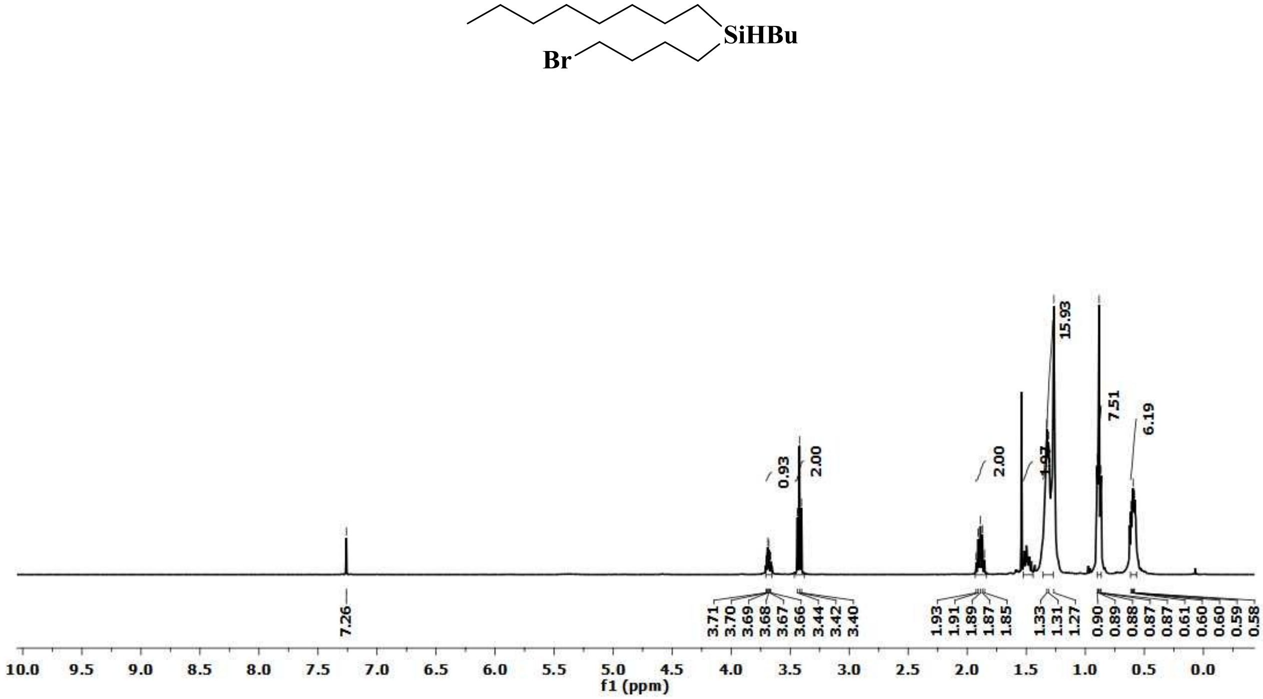

Fig. S71 ^{13}C NMR spectrum ($CDCl_3$) of (4-bromobutyl)(octyl)(butyl)silane.

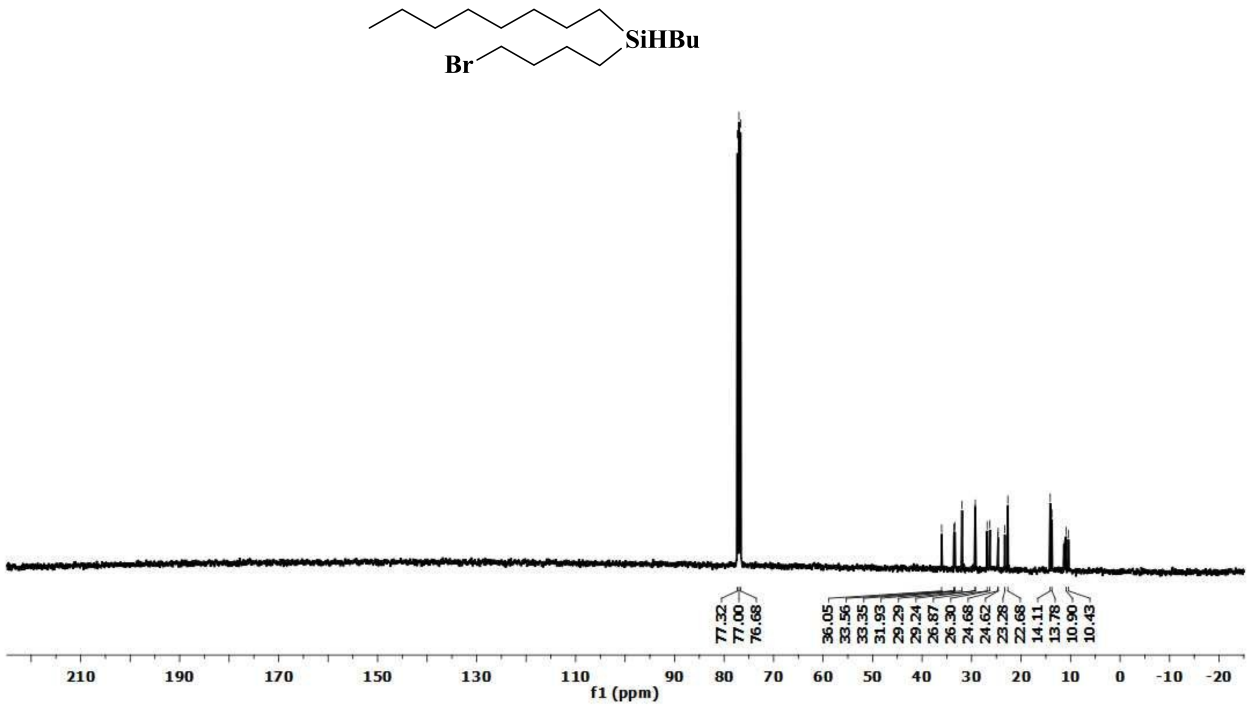

Fig. S72 ^{29}Si NMR spectrum ($CDCl_3$) of (4-bromobutyl)(octyl)(butyl)silane.

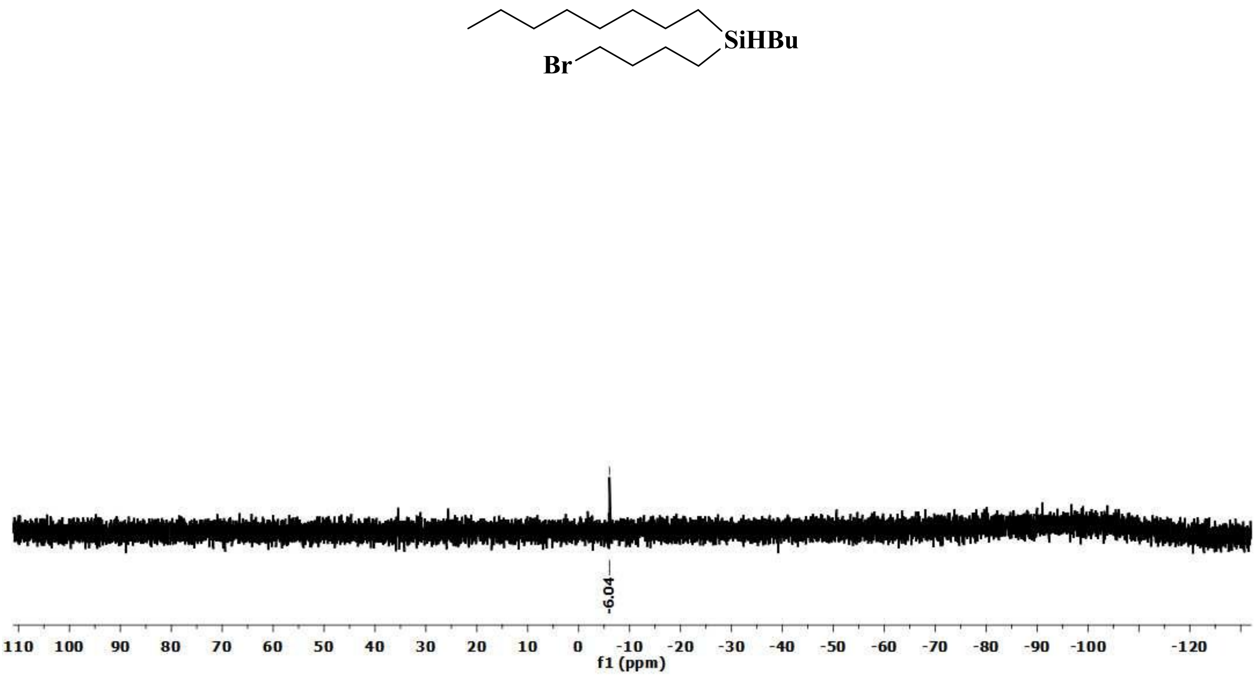

Fig. S73 ^{1}H NMR spectrum ($CDCl_3$) of (4-bromobutyl)(5-bromopentyl)(phenyl)silane.

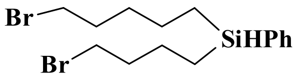

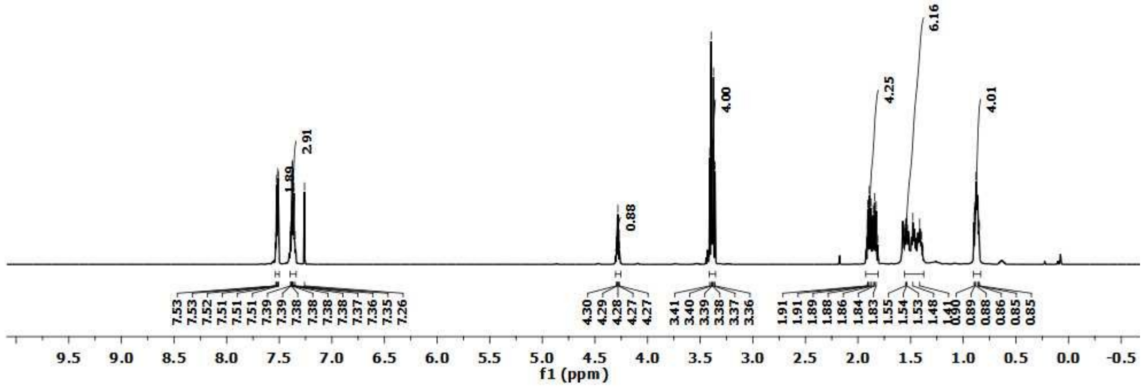

Fig. S74 ^{13}C NMR spectrum ($CDCl_3$) of (4-bromobutyl)(5-bromopentyl)(phenyl)silane.

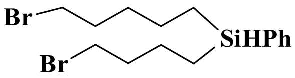

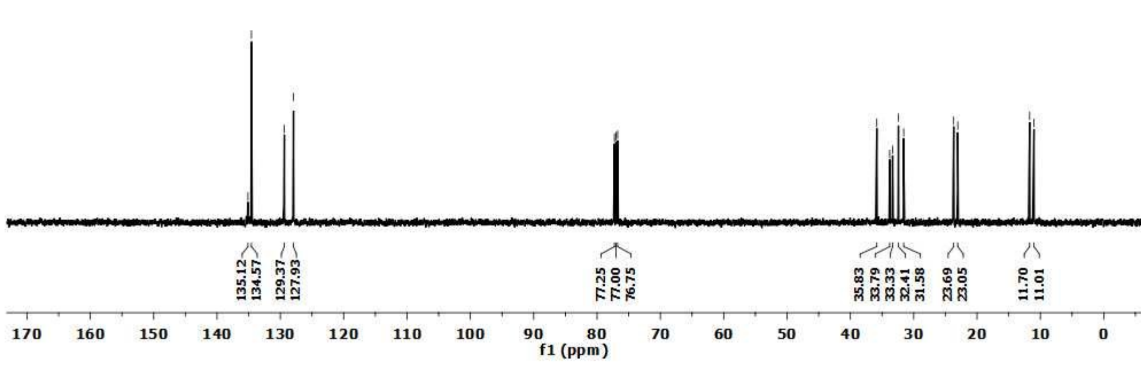

Fig. S75 ^{29}Si NMR spectrum ($CDCl_3$) of (4-bromobutyl)(5-bromopentyl)(phenyl)silane.

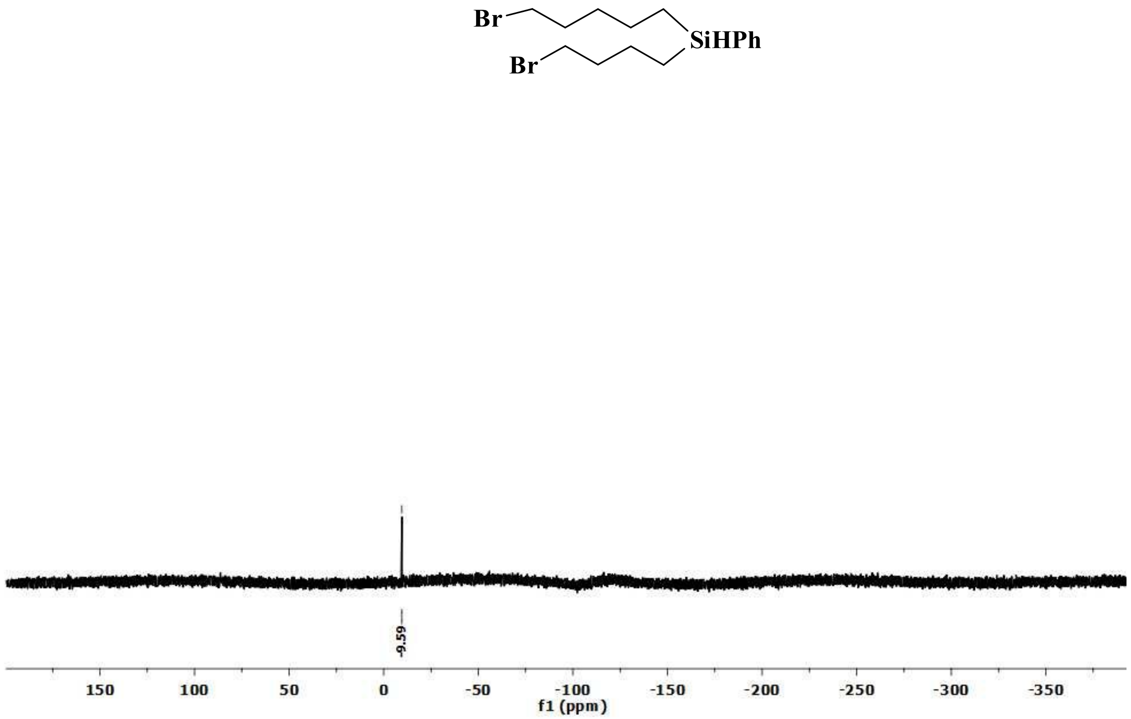

Fig. S76 ^{1}H NMR spectrum ($CDCl_3$) of octyl(3-phenoxypropyl)(phenyl)silane.

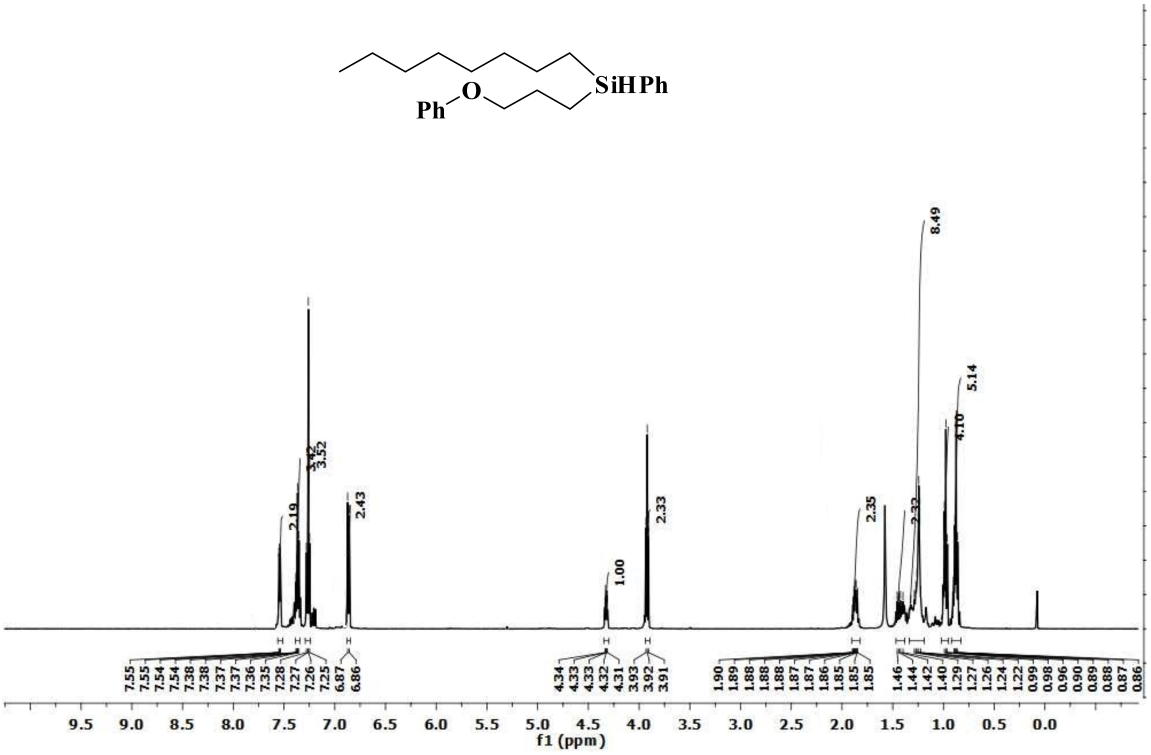

Fig. S77 ^{13}C NMR spectrum ($CDCl_3$) of octyl(3-phenoxypropyl)(phenyl)silane

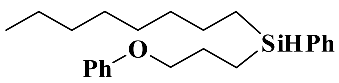

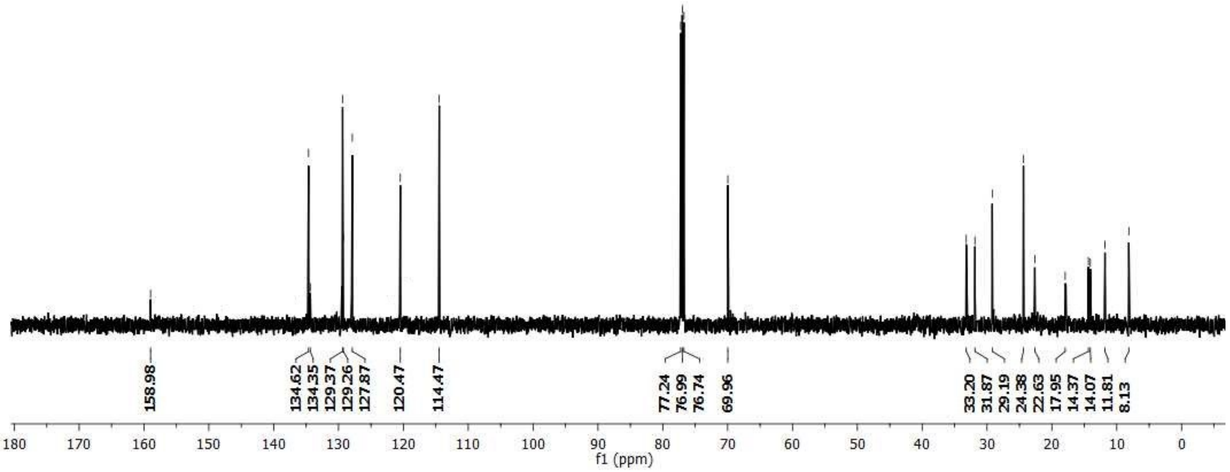

Fig. S78 ^{29}Si NMR spectrum ($CDCl_3$) of octyl(3-phenoxypropyl)(phenyl)silane

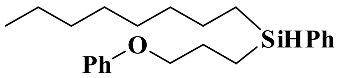

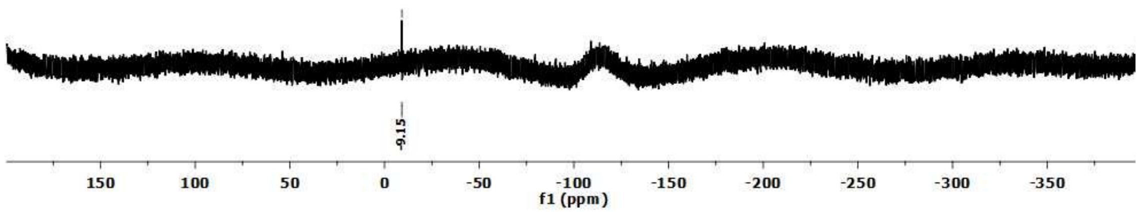

Fig. S79 ^{1}H NMR spectrum ($CDCl_3$) of Bis(trimethylsiloxy)methyloctylsilane.

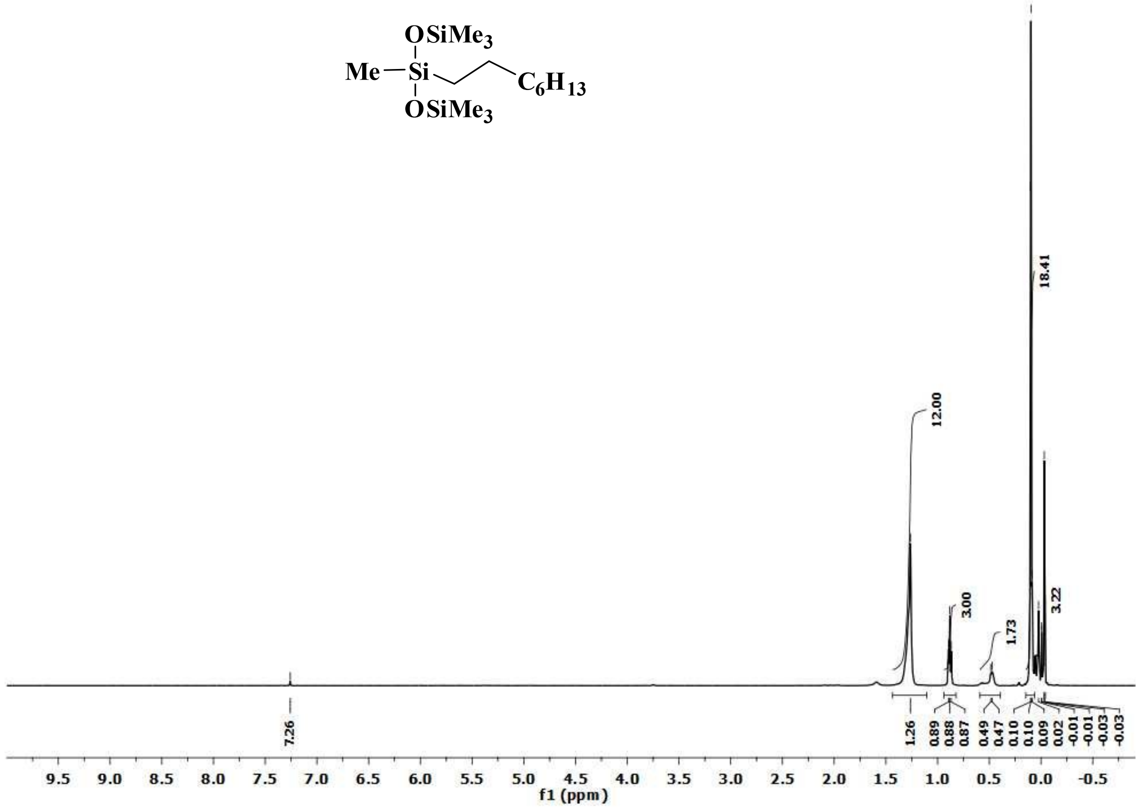

Fig. S80 ^{13}C NMR spectrum ($CDCl_3$) of Bis(trimethylsiloxy)methyloctylsilane.

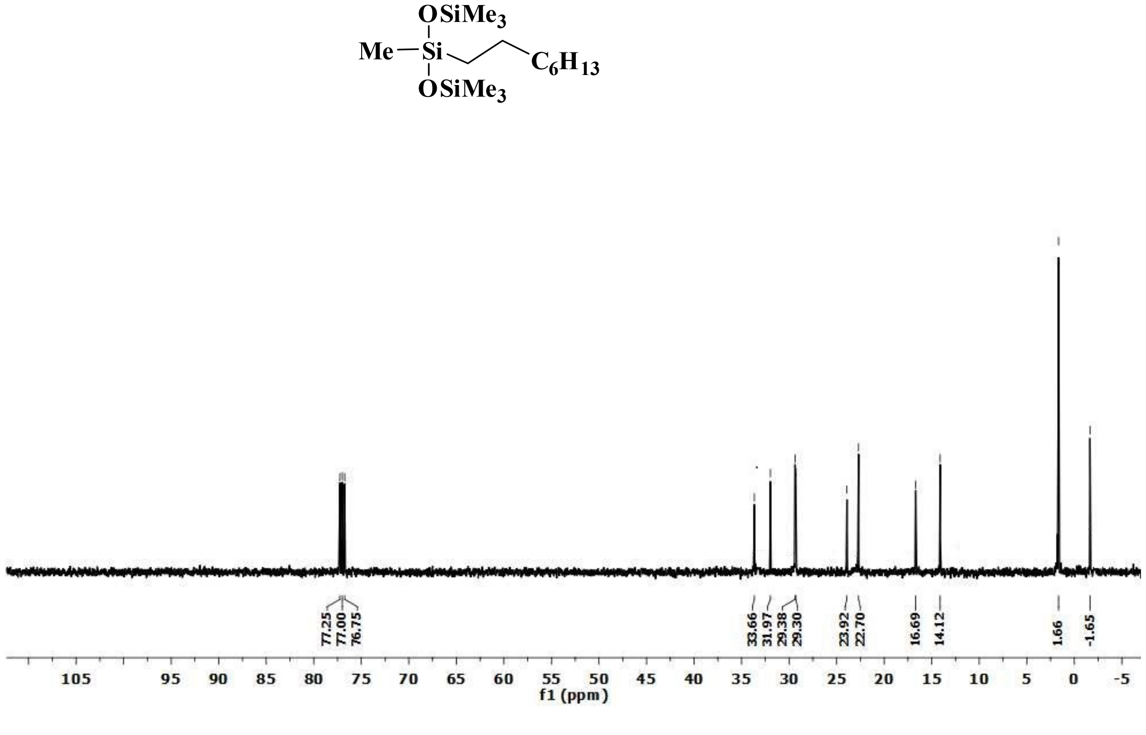

Fig. S81 ^{29}Si NMR spectrum ($CDCl_3$) of Bis(trimethylsiloxy)methyloctylsilane.

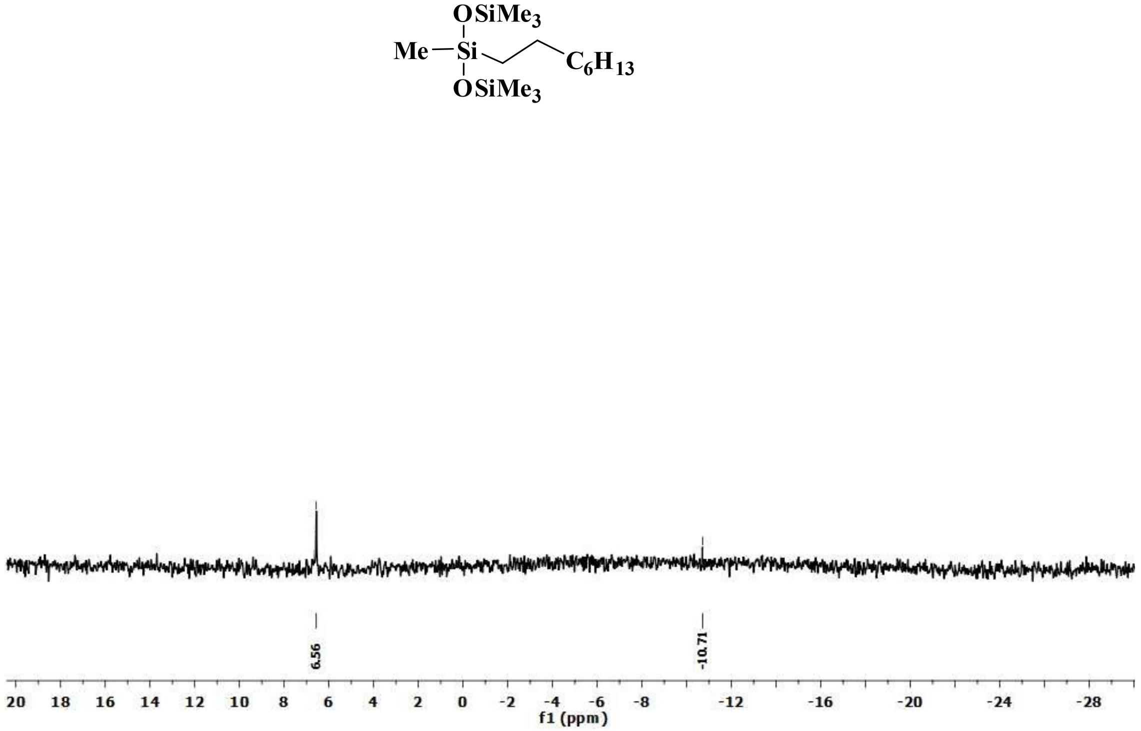

Fig. S82 ^{1}H NMR spectrum ($CDCl_3$) of Bis(trimethylsiloxy)methylhexylsilane.

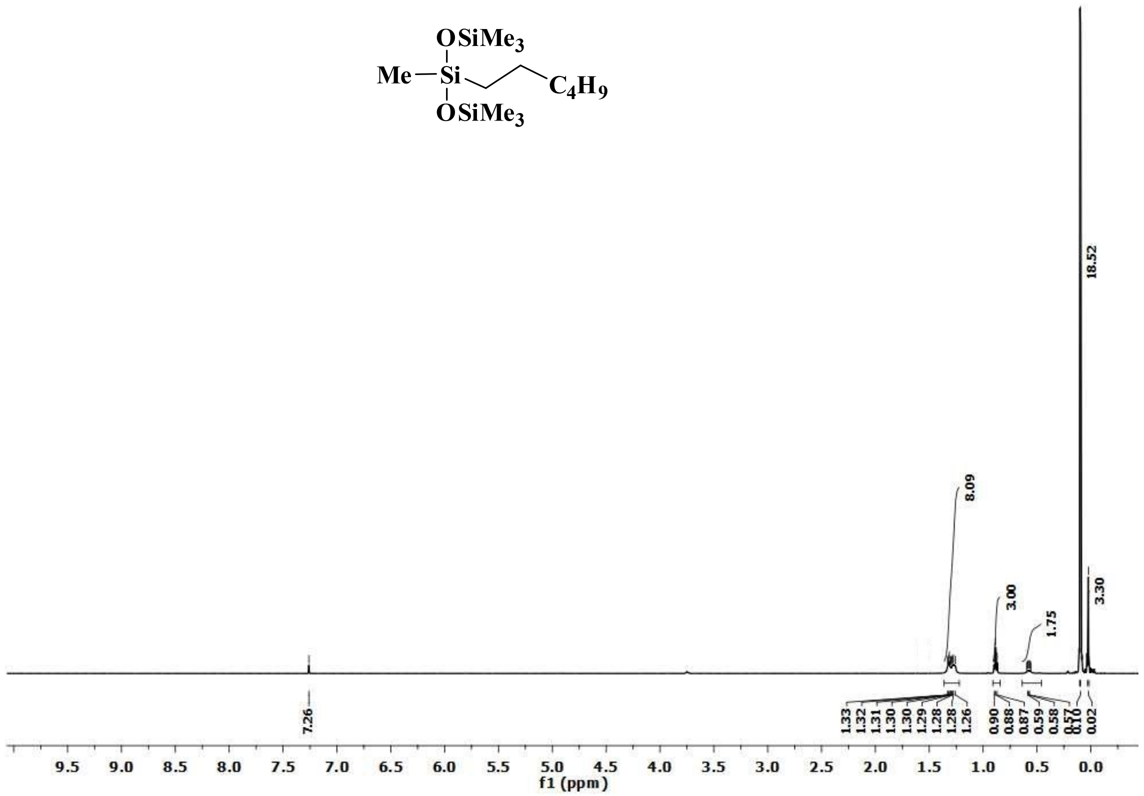

Fig. S83 ^{13}C NMR spectrum ($CDCl_3$) of Bis(trimethylsiloxy)methylhexylsilane.

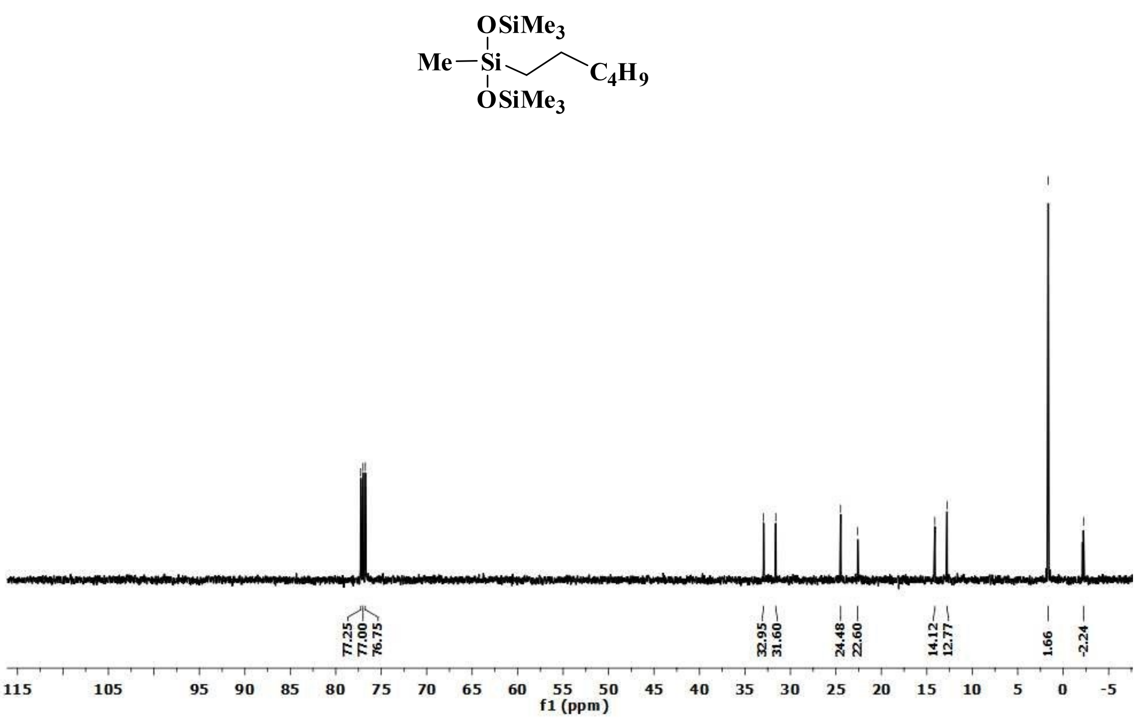

Fig. S84 ^{29}Si NMR spectrum ($CDCl_3$) of Bis(trimethylsiloxy)methylhexylsilane.

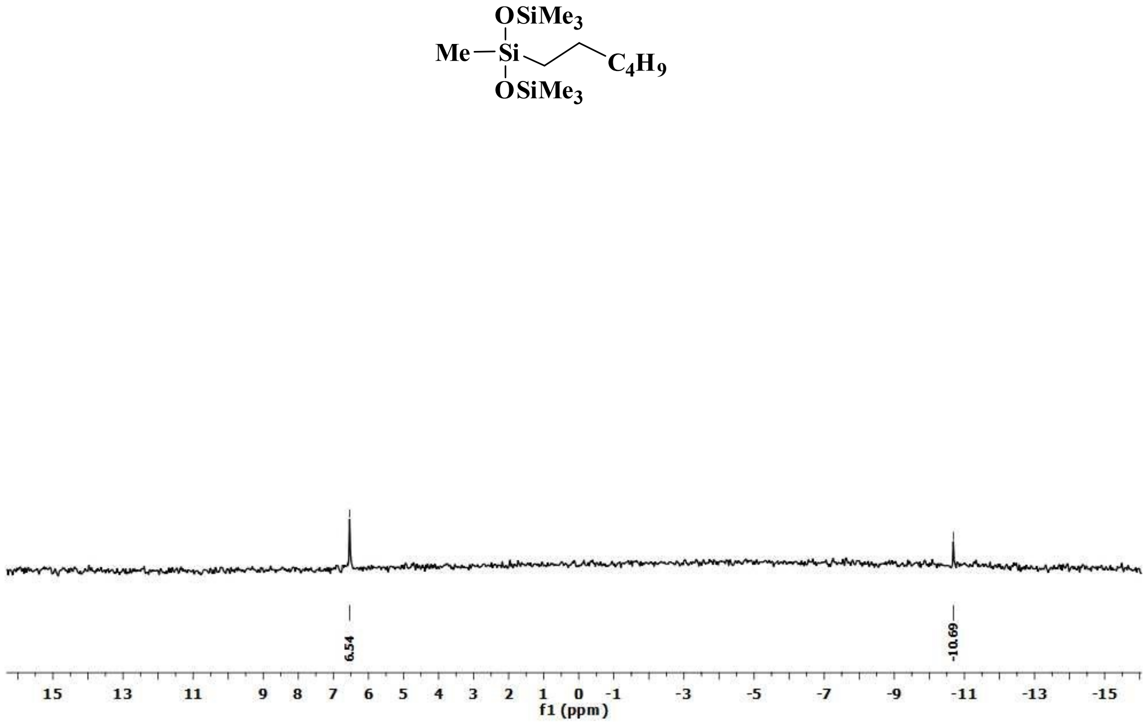

Fig. S85 ^{1}H NMR spectrum ($CDCl_3$) of 1-octene functionalized PMHS.

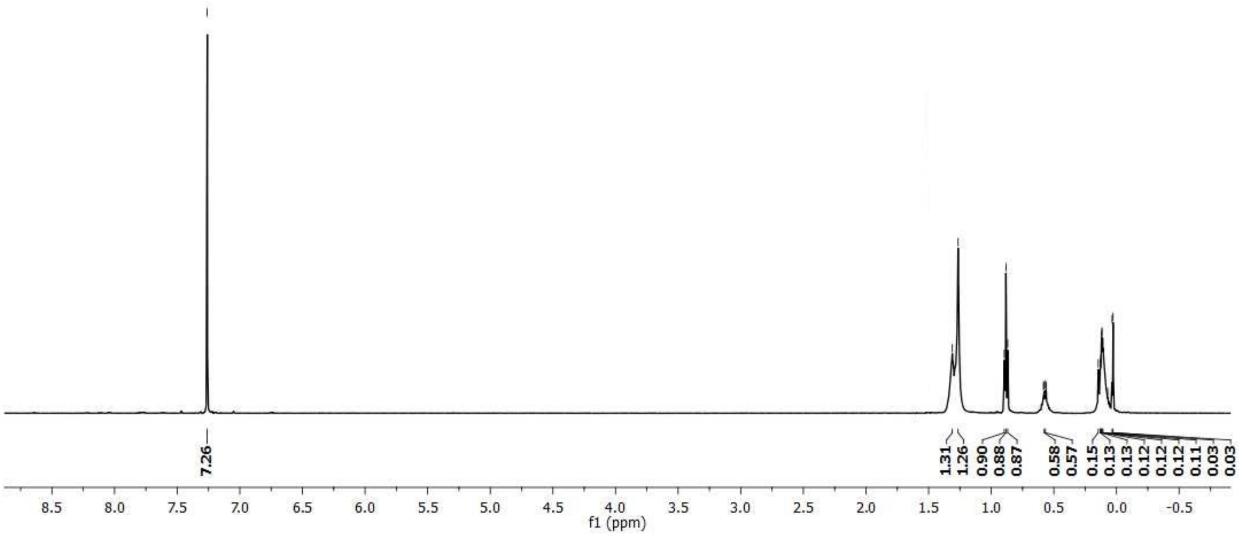

Fig. S86 ^{13}C NMR spectrum ($CDCl_3$) of 1-octene functionalized PMHS.

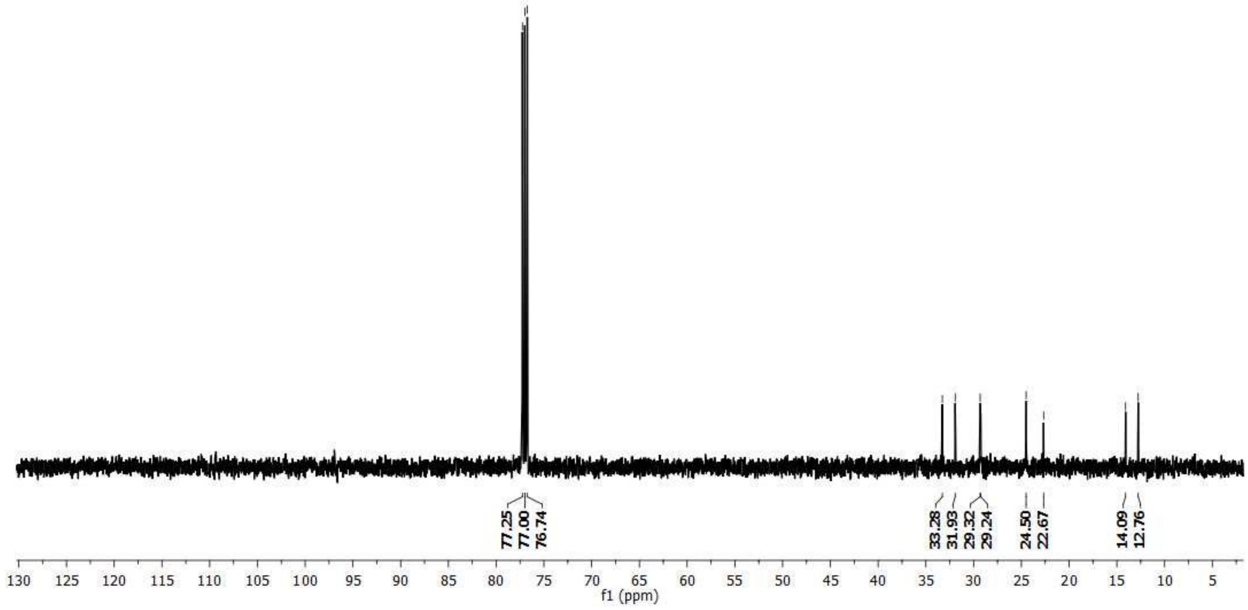

Fig. S87 ^{1}H NMR spectrum ($CDCl_3$) of 1-hexene functionalized PMHS.

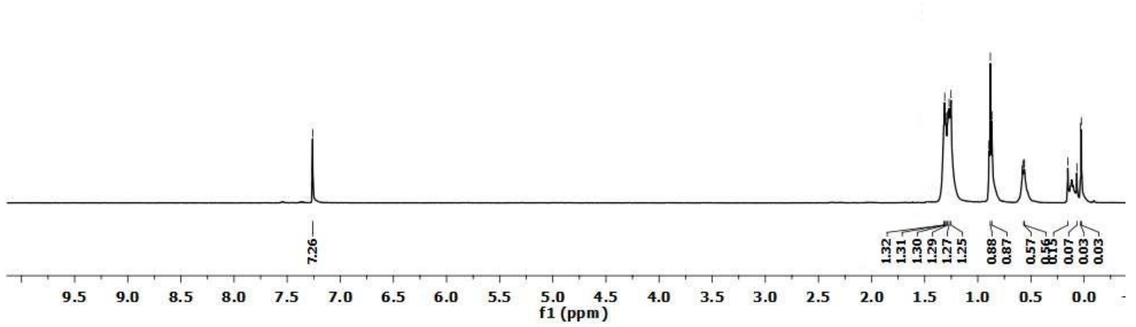

Fig. S88 ^{13}C NMR spectrum ($CDCl_3$) of 1-hexene functionalized PMHS.

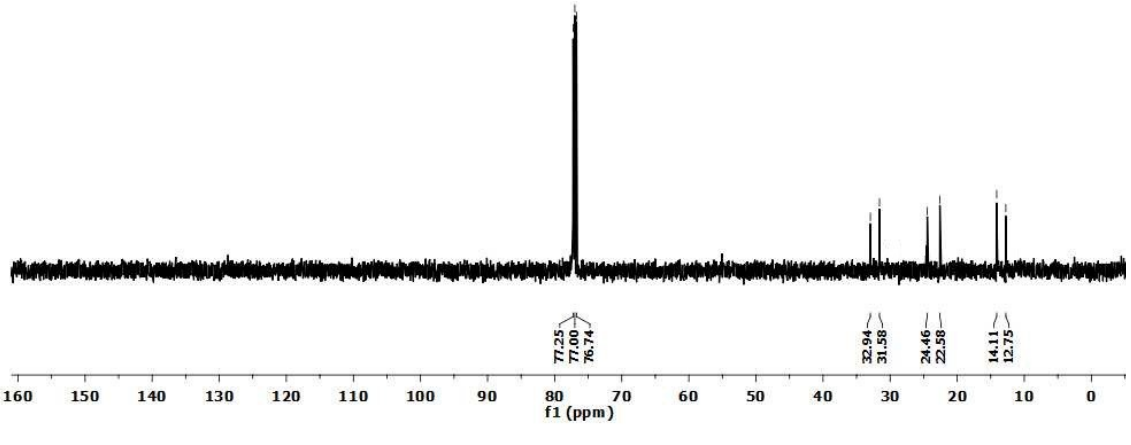

11. Radical trapping by TEMPO.

a. Trapping of diphenyl silyl radical (6).

1 (0.25 mmol) and K (0.75 mmol) in THF (1 mL) were taken and diphenylsilane (0.25 mmol) and TEMPO (0.25 mmol) were added to it at room temperature. The reaction was stirred at room temperature for 4 h and then the reaction mixture was subjected to HRMS characterization in acetonitrile solvent. While trapping and characterizing these intermediates we followed earlier report by Koenig and coworkers.[6]

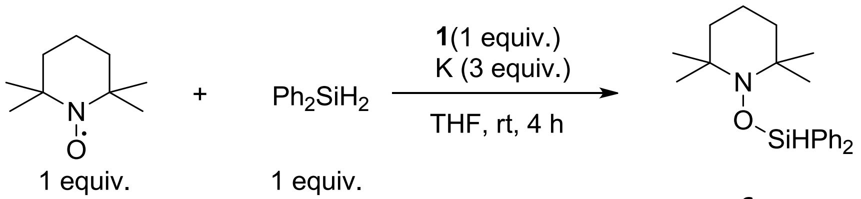

6

Fig. S89 HRMS spectrum of reaction mixture showing formation of **6**

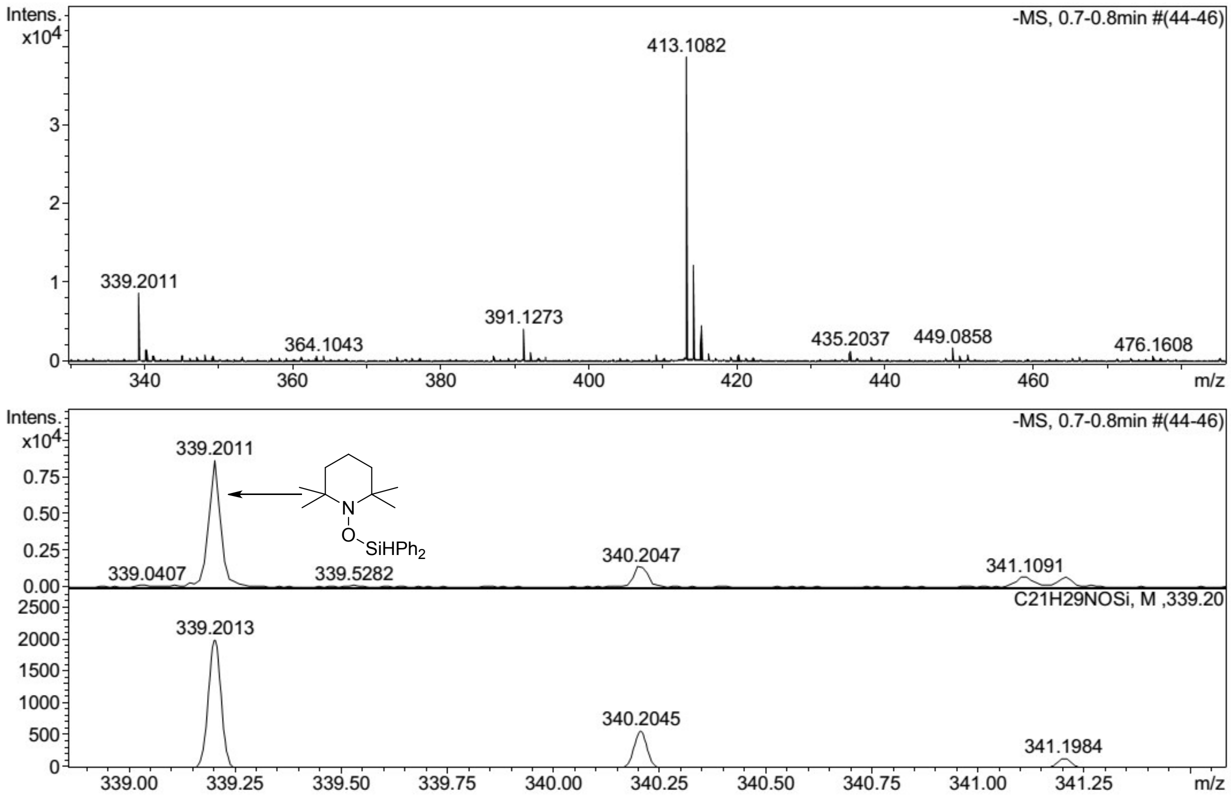

b. Trapping of silylated alkyl radical (7).

1 (0.25 mmol) and K (0.75 mmol) in THF (1 mL) were taken and diphenylsilane (0.25 mmol), 1-hexene (0.25 mmol) and TEMPO (0.25 mmol) were added to it at room temperature. The reaction was stirred at room temperature for 4 h and then the reaction mixture was taken for HRMS characterization in acetonitrile solvent. A trace amount of species **7** was found in the mass spectrum.

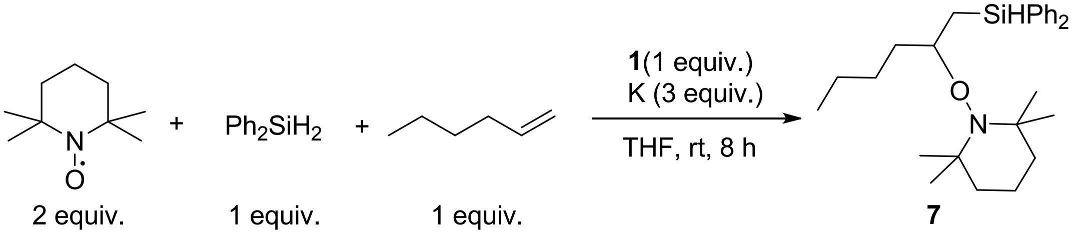

Fig. S90 HRMS spectrum of reaction mixture displaying formation of **7**

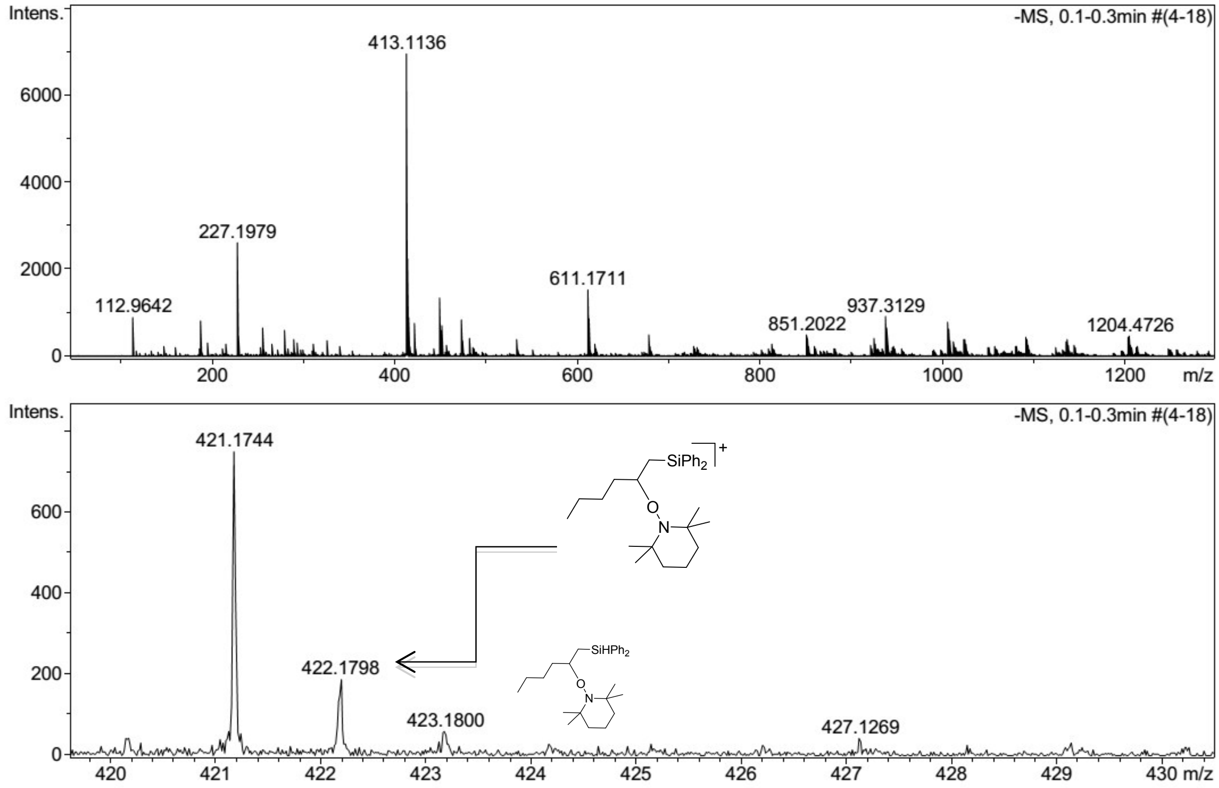

12. X-ray crystallographic details.

Table S1 Crystal data and details of the structure determination for **1**.

CCDC	1518117
Empirical formula	$C_{17}H_{15}Ni_{0.5}O_3$, C_4H_8O
Formula weight	368.75
Temperature (K)	293 K
Wavelength (A˚)	0.71073

Crystal system	monoclinic
Space group	P21/n
Unit cell dimensions	
a (Å)	9.6006(9)
b (Å)	15.4568(16)
c (Å)	11.7534(11)
α (°)	90
β (°)	93.542(9)
γ(°)	90
Volume (Å^3)	1740.8(3)
Z	4
Calculated density (Mg/m3)	1.407
Absorption coefficient (mm-1)	0.614
F(000)	780
Crystal size (mm)	0.25x 0.47 x 0.62
Theta range for data collection(°)	3.0, 25.0
Dataset	-11: 11 ; -18: 15 ; -12: 13
Tot.,Uniq. Data, R(int)	6724, 3066, 0.052
Observed Data [I > 0.0 sigma(I)]	2478
Nref, Npar	3066, 232
R, wR2, S	0.0578, 0.1734, 1.07
Min. and Max. Resd. Dens. [e/Ang^3]	-0.55, 0.81

Table S2 Selected bond distances (Å) and angles (°) for **1**.

Complex **1**			
	Bond distances (Å)		Bond angles (°)
Ni1 -O1	1.980(2)	O1 -Ni1 -O2	89.47(8)
Ni1 -O2	1.990(2)	O1 -Ni1 -O3	90.63(9)
Ni1 -O3	2.154(2)	O1 -Ni1 -O1_a	180.00
O1 -C1	1.262(4)	O1 -Ni1 -O2_a	90.53(8)
O2 -C9	1.269(4)	O1 -Ni1 -O3_a	89.38(9)
O3 -C10	1.410(5)	O2 -Ni1 -O3	88.66(8)
O3 -C13	1.442(4)	Ni1 -O1 -C1	128.4(2)
		Ni1 -O2 -C9	128.79(17)

Table S3 Final coordinates and equivalent isotropic displacement parameters of the non-hydrogen atoms for **1**

Atom	x	y	z	U(eq) [Ang^2]
----	---	---	---	-----------
Ni1	0	1/2	1/2	0.0164(2)
O1	0.1146(2)	0.41194(15)	0.58470(16)	0.0193(6)

O2	0.0667(2)	0.45452(15)	0.35456(16)	0.0197(7)
O3	0.1733(2)	0.58879(16)	0.50856(18)	0.0232(7)
C1	0.1810(3)	0.3493(2)	0.5454(3)	0.0176(9)
C2	0.2409(3)	0.2874(2)	0.6274(2)	0.0222(10)
C3	0.3155(3)	0.2190(2)	0.5958(3)	0.0254(10)
C3A	0.3401(3)	0.2032(2)	0.4782(3)	0.0241(10)
C4	0.4187(3)	0.1309(2)	0.4455(3)	0.0299(11)
O4	0.6567(3)	0.6083(2)	0.0888(2)	0.0553(10)
C5	0.4442(3)	0.1177(3)	0.3323(3)	0.0306(11)
C6	0.3950(3)	0.1758(3)	0.2508(3)	0.0290(10)
C6A	0.3161(3)	0.2481(2)	0.2799(3)	0.0223(10)
C7	0.2660(3)	0.3090(2)	0.1964(3)	0.0268(10)
C8	0.1864(3)	0.3770(2)	0.2240(3)	0.0238(10)
C9	0.1483(3)	0.3916(2)	0.3393(2)	0.0192(9)
C9A	0.2040(3)	0.3348(2)	0.4265(2)	0.0179(9)
C9B	0.2861(3)	0.2621(2)	0.3950(3)	0.0203(9)
C10	0.2005(4)	0.6407(3)	0.6060(3)	0.0384(11)
C11	0.3401(4)	0.6846(3)	0.5947(3)	0.0321(11)
C12	0.4019(3)	0.6377(3)	0.4943(3)	0.0310(11)
C13	0.3064(3)	0.5611(2)	0.4712(3)	0.0278(10)
C14	0.5154(5)	0.6329(3)	0.0949(5)	0.0687(18)
C15	0.4389(4)	0.5615(4)	0.1481(4)	0.0647(19)
C16	0.5458(4)	0.5197(4)	0.2195(4)	0.0518(16)
C17	0.6788(4)	0.5306(3)	0.1586(4)	0.0457(14)

Table S4 (An)isotropic Displacement Parameter for **1**

Atom	U(1,1) or U	U(2,2)	U(3,3)	U(2,3)	U(1,3)	U(1,2)

---- ------ ------ ------ ------ ------

Ni1	0.0171(4)	0.0166(4)	0.0158(4)	-0.0009(2)	0.0033(2)	-0.0007(2)
O1	0.0195(10)	0.0212(13)	0.0176(10)	-0.0005(9)	0.0056(8)	0.0030(10)
O2	0.0213(11)	0.0207(13)	0.0174(10)	-0.0015(10)	0.0042(8)	0.0009(10)
O3	0.0196(10)	0.0244(14)	0.0260(11)	-0.0053(10)	0.0037(9)	-0.0034(10)
C1	0.0150(14)	0.0153(17)	0.0230(15)	-0.0013(14)	0.0060(12)	-0.0047(13)
C2	0.0249(16)	0.024(2)	0.0177(14)	0.0058(14)	0.0016(12)	0.0017(14)
C3	0.0236(16)	0.024(2)	0.0287(17)	0.0042(15)	0.0014(13)	0.0022(15)
C3A	0.0181(15)	0.0219(18)	0.0318(17)	-0.0040(15)	-0.0019(13)	-0.0029(14)
C4	0.0230(16)	0.025(2)	0.0413(19)	-0.0021(17)	-0.0018(14)	0.0013(15)
O4	0.0388(15)	0.067(2)	0.0604(18)	0.0180(17)	0.0051(14)	-0.0084(16)
C5	0.0195(15)	0.027(2)	0.045(2)	-0.0131(17)	0.0006(15)	0.0037(15)
C6	0.0211(16)	0.034(2)	0.0327(18)	-0.0181(17)	0.0079(14)	-0.0038(16)
C6A	0.0156(15)	0.026(2)	0.0255(16)	-0.0070(14)	0.0038(12)	-0.0021(14)
C7	0.0236(16)	0.035(2)	0.0225(16)	-0.0097(15)	0.0072(13)	-0.0004(16)
C8	0.0243(16)	0.028(2)	0.0189(15)	-0.0003(14)	-0.0001(12)	-0.0044(15)
C9	0.0169(14)	0.0198(18)	0.0210(15)	-0.0044(14)	0.0029(12)	-0.0037(14)
C9A	0.0170(14)	0.0158(17)	0.0210(15)	-0.0016(13)	0.0028(11)	-0.0043(13)
C9B	0.0137(14)	0.0211(18)	0.0265(16)	-0.0057(14)	0.0048(12)	-0.0017(14)
C10	0.0280(18)	0.037(2)	0.051(2)	-0.026(2)	0.0086(16)	-0.0081(18)
C11	0.0305(18)	0.027(2)	0.0384(19)	-0.0089(17)	-0.0011(15)	-0.0060(17)
C12	0.0206(16)	0.030(2)	0.043(2)	-0.0032(17)	0.0062(14)	-0.0047(15)
C13	0.0195(16)	0.032(2)	0.0329(17)	-0.0134(16)	0.0094(13)	-0.0044(15)
C14	0.040(2)	0.044(3)	0.120(4)	0.010(3)	-0.013(3)	0.003(2)
C15	0.048(3)	0.084(4)	0.064(3)	0.024(3)	0.018(2)	0.026(3)
C16	0.039(2)	0.059(3)	0.057(3)	0.019(2)	0.001(2)	0.001(2)

C17	0.033(2)	0.052(3)	0.051(2)	-0.016(2)	-0.0050(18)	0.008(2)

13. Computational Details

Coordinates of the computationally investigated structures

1

0 3

C	3.47919700	-0.00017900	-2.48142800
C	2.69092600	-0.00007400	-1.26413900
C	3.39316200	-0.00009000	-0.00001900
C	4.82974700	-0.00019200	-0.00002600
C	5.56691400	-0.00029000	-1.22451100
C	4.83915300	-0.00027900	-2.46085000
C	2.69093800	0.00001000	1.26410700
C	5.56692500	-0.00020300	1.22445400
C	4.83917500	-0.00010100	2.46079900
C	3.47921800	-0.00000200	2.48138900
C	6.97232100	-0.00030800	1.20333600
C	7.67743800	-0.00040500	-0.00003800
C	6.97231100	-0.00039400	-1.20340500
H	7.50988300	-0.00046800	-2.14942300
H	8.76370900	-0.00048600	-0.00004200
H	2.92053200	-0.00016600	-3.41285400
H	5.40095600	-0.00035000	-3.39329500
H	2.92056200	0.00007500	3.41282000
C	-6.97230200	0.00040800	1.20342400
C	-5.56690500	0.00029800	1.22452400
C	-4.83913900	0.00027400	2.46086000
C	-7.67743400	0.00042500	0.00006000
C	-4.82974300	0.00020000	0.00003500
C	-5.56692600	0.00021500	-1.22444100
C	-6.97232200	0.00032700	-1.20331700
C	-4.83918000	0.00011000	-2.46078900
H	-5.40099500	0.00011500	-3.39322700
C	-3.47922400	0.00000500	-2.48138400

C	-2.69093800	-0.00001000	-1.26410600
C	-3.39315800	0.00009700	0.00002400
C	-2.69091800	0.00008000	1.26414100
C	-3.47918300	0.00017200	2.48143300
H	-2.92051400	0.00015200	3.41285600
H	-8.76370400	0.00051200	0.00006900
H	-7.50990600	0.00033600	-2.14932700
H	-2.92057200	-0.00007900	-3.41281800
H	5.40098600	-0.00010800	3.39323900
H	7.50990100	-0.00031500	2.14934800
H	-5.40093800	0.00034400	3.39330800
H	-7.50987000	0.00048200	2.14944400
O	-1.42024600	-0.00009700	-1.40647400
O	-1.42022300	-0.00001100	1.40648600
O	1.42023200	0.00000400	-1.40649000
O	1.42024500	0.00011100	1.40646800
Ni	0.00000200	-0.00000200	-0.00000100
C	0.00002900	3.06299800	1.10950400
C	0.00180600	4.36524600	0.72000300
H	-0.00053900	2.54548900	2.05567600
C	0.00177700	4.36520100	-0.72030800
H	0.00293100	5.22678100	1.37357200
H	0.00288000	5.22669500	-1.37393100
C	0.00002600	-3.06301900	-1.10952500
C	-0.00181800	-4.36526600	-0.72002100
H	0.00067900	-2.54551400	-2.05570000
C	-0.00181500	-4.36521600	0.72029000
H	-0.00294700	-5.22680300	-1.37358700
H	-0.00293800	-5.22670800	1.37391600
C	-0.00003500	3.06292900	-1.10972700
C	-0.00003800	-3.06294300	1.10970400
O	-0.00115000	2.25358900	-0.00008600
O	0.00115000	-2.25360600	0.00006100
H	-0.00067700	2.54536000	-2.05586700
H	0.00053600	-2.54537100	2.05584200

single -electron reduced pdt

-1 4

C	0.00000000	0.00000000	0.00000000
C	0.00000000	0.00000000	1.44320170
C	1.28461375	0.00000000	2.12741640
C	2.49805687	0.00000000	1.34313444
C	2.45034287	0.00000000	-0.09823787
C	1.16646387	0.00000000	-0.73960466
C	1.38087341	-0.00000000	3.57970143
C	3.79266279	0.00000000	1.97860426
C	3.85015975	0.00000000	3.41261908
C	2.69675046	0.00000000	4.17241512
C	4.97524109	-0.00000000	1.18871611
C	4.90753819	-0.00000000	-0.21418175
C	3.65622226	-0.00000000	-0.85207702
H	3.59433030	-0.00000000	-1.93948741
H	5.82166577	-0.00000000	-0.80500709
H	-0.97807745	-0.00000000	-0.47494444
H	1.12638999	-0.00000000	-1.82852003
H	2.72809481	0.00000000	5.25925686
C	-6.87488145	-0.00017993	8.84744587
C	-5.66897034	-0.00014163	8.09365766
C	-4.38511765	-0.00011612	8.73507955
C	-8.12617084	-0.00020520	8.20949772
C	-5.71662414	-0.00012858	6.65228454
C	-7.01120375	-0.00015539	6.01675963
C	-8.19381546	-0.00019254	6.80659792
C	-7.06864106	-0.00014267	4.58274317
H	-8.04500603	-0.00016284	4.09895253
C	-5.91519806	-0.00010565	3.82299559
C	-4.59934900	-0.00007788	4.41576547
C	-4.50314845	-0.00008979	5.86805298
C	-3.21856071	-0.00006372	6.55232140
C	-3.21862397	-0.00007897	7.99552468

H	-2.24056481	-0.00005939	8.47050715
H	-9.04032368	-0.00023520	8.80028581
H	-9.15984675	-0.00021195	6.30353120
H	-5.94650022	-0.00009570	2.73615278
H	4.82654530	0.00000000	3.89636784
H	5.94129403	0.00000000	1.69174264
H	-4.34509093	-0.00012663	9.82399564
H	-6.81303503	-0.00018974	9.93485950
O	-3.57059656	-0.00004338	3.58827282
O	-2.04151717	-0.00002771	5.95428382
O	-1.17701991	0.00000000	2.04129425
O	0.35216223	-0.00000000	4.40723766
Ni	-1.60922027	-0.00001679	3.99780845
C	-0.99499240	3.00091272	4.94899258
C	-1.21513057	4.31270423	4.60828171
H	-0.48428186	2.47656507	5.73970413
C	-2.00434844	4.31230823	3.38633743
H	-0.86400413	5.17794208	5.15202046
H	-2.35580927	5.17719539	2.84225619
C	-2.22397297	-3.00056129	3.04606100
C	-2.00417221	-4.31255277	3.38621480
H	-2.73461638	-2.47573264	2.25562281
C	-1.21483449	-4.31287978	4.60807921
H	-2.35559840	-5.17746962	2.84215780
H	-0.86356331	-5.17808691	5.15177458
C	-2.22398630	3.00029871	3.04614406
C	-0.99479455	-3.00107028	4.94878188
O	-1.60932998	2.18111621	3.99773288
O	-1.60930890	-2.18131779	3.99759309
H	-2.73450236	2.47542736	2.25565032
H	-0.48404125	-2.47669793	5.73945090

2

-2 3

C	0.00000000	0.00000000	0.00000000
C	0.00000000	0.00000000	1.42966146

C	1.28700289	0.00000000	2.11364037
C	2.50299241	0.00000000	1.32919025
C	2.45477849	0.00000000	-0.11868284
C	1.17728624	0.00000000	-0.75125198
C	1.37509411	-0.00000000	3.57243763
C	3.80109263	0.00000000	1.97035755
C	3.84889102	0.00000000	3.39659059
C	2.68305101	0.00000000	4.15918626
C	4.99284632	-0.00000000	1.17389147
C	4.92153298	-0.00000000	-0.22945471
C	3.67332956	-0.00000000	-0.87511685
H	3.61231505	-0.00000000	-1.96390977
H	5.83905110	-0.00000000	-0.82037247
H	-0.97777250	-0.00000000	-0.47943088
H	1.13044475	-0.00000000	-1.84151879
H	2.71533438	0.00000000	5.24701085
C	-6.94820389	0.00052092	8.90403834
C	-5.72972998	0.00043955	8.14807431
C	-4.45204732	0.00041763	8.78097426
C	-8.19625328	0.00054121	8.25810107
C	-5.77776815	0.00037783	6.70016807
C	-7.07571940	0.00040093	6.05858527
C	-8.26731722	0.00048770	6.85469357
C	-7.12315414	0.00034012	4.63204079
H	-8.09750437	0.00035702	4.14042987
C	-5.95697621	0.00026056	3.87019249
C	-4.64960332	0.00023471	4.45758697
C	-4.56166032	0.00029532	5.91599567
C	-3.27512700	0.00027454	6.60017977
C	-3.27501242	0.00033810	8.02968071
H	-2.29720851	0.00032110	8.50909928
H	-9.11389153	0.00060371	8.84882014
H	-9.23343847	0.00050318	6.34892476
H	-5.98875987	0.00021402	2.78233206
H	4.82344682	0.00000000	3.88788645
H	5.95902104	-0.00000000	1.67956876
H	-4.40531716	0.00046428	9.87121024
H	-6.88751230	0.00056596	9.99284643
O	-3.60625019	0.00015737	3.63562945
O	-2.08572639	0.00020048	5.98429180
O	-1.18905620	0.00000000	2.04535605
O	0.33246319	-0.00000767	4.39434804
Ni	-1.63861714	0.00009633	4.01609326
C	-1.60460318	3.01570437	5.14521766
C	-1.61600158	4.32981530	4.74029778
H	-1.58284005	2.48188222	6.08193599
C	-1.65919333	4.32828767	3.28600826
H	-1.59992693	5.19422509	5.38931729
H	-1.67446935	5.19133275	2.63515915
C	-1.67185768	-3.01334462	2.88419326
C	-1.65956595	-4.32822946	3.28656131

H	-1.69371556	-2.47744334	1.94863795
C	-1.61633284	-4.32954005	4.74085015
H	-1.67492166	-5.19137057	2.63584375
H	-1.60029430	-5.19385305	5.39000026
C	-1.67155820	3.01334479	2.88383665
C	-1.60483769	-3.01536872	5.14557135
O	-1.63842993	2.20039164	4.01540848
O	-1.63864412	-2.20022462	4.01564044
H	-1.69340678	2.47730103	1.94836177
H	-1.58301251	-2.48140492	6.08220689

14. References

1. K. Kamata, A. Suzuki, Y. Nakai and H. Nakazawa, *Organometallics*, 2012, **31**, 3825−3828.

2. L. Nielsen and T. Skrydstrup, *J. Am Chem. Soc.*, 2008, **130**, 13145–13151.

3. T. Takahashi, M. Hasegawa, N. Suzuki, M. Saburi, C. J. Rousset and E. Negishi, *J. Am. Chem. Soc.*, 1991, **113**, 8564-8566.

4. Y. Sunada, H. Tsutsumi, K. Shigeta, R. Yoshida, T. Hashimoto and H. Nagashima, *Dalton Trans.*, 2013, **42**, 16687–16692.

5. T. G. Diamant, M. L. Zanota, R. Sayah, L. Veyre, C. Nikitine, C. Bellefon, S. Marrot, V. Meille and C. Thieuleux, *Chem. Commun.*, 2015, **51**, 16194-16196.

6. D. P. Hari, P. Schroll and B. König, *J. Am. Chem. Soc.*, 2012, **134**, 2958−2961.
